# Fe(III) complexes with prolonged luminescence lifetimes via excited-state equilibration promoted by reversible intercomponent electron transfer

**DOI:** 10.1038/s41467-026-71767-4

**Published:** 2026-04-11

**Authors:** Salvatore Genovese, Ambra M. Cancelliere, Antonino Arrigo, Alessandro Auditore, Antonino Licciardello, Manuel Pedrón, Ilaria Ciofini, Fausto Puntoriero, Sebastiano Campagna

**Affiliations:** 1 Department of Chemical, Biological, Pharmaceutical, and Environmental Sciences, University of Messina, Messina, Italy; 2 Department of Chemical Sciences, University of Catania, Catania, Italy; 3 Institute of Chemistry for Life and Health Sciences, ENSCP, Chimie ParisTech, Paris, France

**Keywords:** Photochemistry, Excited states

## Abstract

Achieving long-lived luminescence in complexes of earth-abundant metals remains challenging because excited states in first-row transition-metal systems typically deactivate rapidly under ambient conditions. Strategies capable of prolonging emission lifetimes in such compounds are therefore of considerable interest. Here we show that iron(III) complexes incorporating pyrene-functionalized ligands display luminescence in fluid solution at room temperature with lifetimes up to 6.5 ns. Spectroscopic analysis indicates that excited-state equilibration occurs through reversible intramolecular electron transfer from the pyrene unit to the iron centre, generating a charge-separated state. Although the ligand-to-metal charge-transfer state can also undergo reversible energy transfer to nearby pyrene triplet states, intramolecular electron transfer dominates, leading to the formation of a pyrene + -iron(II) charge-separated state that acts as a long-lived excited-state reservoir. Equilibration involving this state produces biphasic emission from the iron centre. These findings identify reversible intercomponent electron transfer as a strategy for achieving prolonged luminescence lifetimes in complexes of earth-abundant metals.

## Abstract (web summary)

Long-lived, luminescent earth-abundant metal complexes are promising for applications based on photoinduced processes. This study shows how equilibrated excited states involving charge separation can prolong emission lifetime of Fe(III) complexes.

## Introduction

Transition metal complexes featuring photoactive properties are at the center of a large interest for application in various fields, ranging from solar energy conversion^1–3^ to light-emitting technologies^4^, bio-imaging^5,6^ and phototherapy^7^. However, such photoactive transition metal complexes are mostly made of precious and relatively less abundant metals, with ruthenium playing the major role^8^, and this represents a serious limitation for their possible use on a large scale. In recent years, many efforts have been devoted to preparing photoactive, luminescent metal complexes made of earth-abundant metals^9–23^. Within this framework, iron compounds look quite interesting, since iron is one of the most abundant metals on Earth. Elusive for years, in recent times iron compounds exhibiting relatively intense luminescence (quantum yields around 2%) have finally been synthesized^10,11,16–18,21^. Despite many efforts, however, the room temperature luminescence lifetimes of iron complexes in solution remain relatively short: in fact, an excited-state lifetime of 2 ns is reported for [Fe(phtmeimb)_2_]^+^ (**1**; phtmeimb = {phenyl[tris(3-methylimidazol-1-ylidene)]borate} anion), the iron(III) compound exhibiting the unquestioned longest-lived luminescence so far^16^ (a luminescence lifetime of about 5 ns has recently been reported for a Fe(III) compound^24^, however that luminescence has lately been questioned as due to an unidentified impurity^25^). Longer-lived luminescence is highly desirable, particularly for solar energy conversion processes involving inter-molecular photoinduced energy and/or electron transfer.

**Fig. 1:**
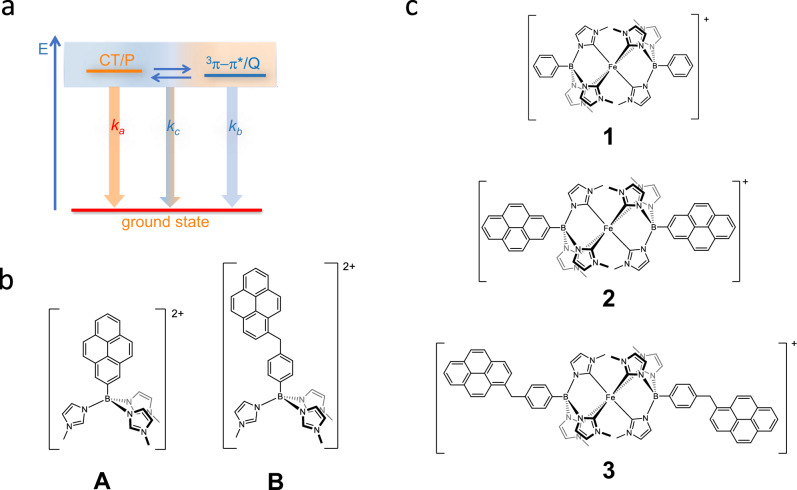
Excited-state equilibration scheme and structural formulas of the compounds. Panel **a**: schematic energy level diagram suitable for excited-state equilibration between a generic charge-transfer (CT) state (or any initially prepared state, P) and a triplet π−π* level (or generically a quencher state, Q; note that Q could also be a charge-separated state); isoenergetic excited states are represented. k_a_ and k_b_ are intrinsic decays (radiative and radiationless transitions) of the “isolated” components, and k_c_ represents the experimental decay of the equilibrated states, depending on the relative percentage of the individual excited states at the equilibrium^34^. Panel **b**: structural formulas of the free ligands **A** and **B**; Panel **c**: structural formulas of the new compounds **2** and **3** and of the model species **1**.

The excited-state equilibration approach is a method which has been used to prolong luminescence lifetime of transition metal complexes^26–34^. It relies on thermal equilibration between the emissive excited state of the metal-based luminophore and a triplet state of suitable organic chromophores, characterized by long-lived excited states. In the presence of suitable conditions (e.g., non-negligible electronic coupling between the involved excited states), vectorial energy transfer from the metal-based chromophore to the organic chromophore triplet excited state can occur. Since the intrinsic decays of the individual chromophores are usually quite different, Boltzmann distribution can allow for excited-state equilibration by reversible energy transfer (when the two involved excited states are close enough in energy), with the result of obtaining metal-based luminescence output sustained by the organic triplet state, which behaves as an energy storage element^26–34^. As a consequence, emission lifetime of the metal-based chromophore can be prolonged. Under these conditions, biexponential decay can be obtained: the shorter component is due to the prompt “quenched” metal-based emission, whereas the longer component (the delayed emission) coincides with the equilibrated state decay^34^. The excited-state equilibration approach is exemplified in Fig. 1, in which the similitude with the thermally-activated delayed fluorescence also appears. In brief, the lifetime of the thermally-equilibrated excited state (1/k_c_, referring to Fig. 1) depends on the intrinsic decay rates k_a_ and k_b_ of the “isolated” subunits P and Q and on the percentage of each excited state in the equilibrated state, in its turn depending on the energy difference between the involved excited states, provided that the rate constants for forward and backward excited-state interconversion are much faster than intrinsic decays k_a_ and k_b_, so that Boltzmann equilibration is effective^34^. For species in which luminescence only originates from P, when *Q is much longer lived than *P (that is, when k_b_ is smaller than k_a_), the lowering of the energy level of Q with respect to the energy level of P leads to a longer-lived equilibrated state, that is a longer-lived P emission. This method has been successfully employed for prolonging luminescence lifetimes of several metal complexes, allowing to reach luminescence lifetimes even longer than 100 μs for Ru(bpy)_3_^2+^-type (bpy = 2,2’-bipyridine) compounds^29,30,34^, but it had never been applied to earth-abundant metal complexes (with the rare exception of semiprecious copper complexes^35^), to the best of our knowledge. In fact, pyrene moieties have been added to Cr(0) species for improving luminescence^14^, but the effect was due to excited-state delocalization^36,37^, and excited-state equilibration was not found. An Fe(II) compound has been decorated with anthracene groups for obtaining excited-state equilibration, but unsuccessfully, probably due to the very short intrinsic lifetime of the Fe(II) subunit (13 ps)^38^, which made inefficient energy transfer to anthracene. Noticeably, during the preparation of the revised version of the present article, an article reporting the use of the excited-state equilibration approach involving anthracene triplet states for obtaining iron(III) complexes with emission lifetimes up to 100 ns has been published^39^. In rare cases, excited-state equilibration has also been obtained when the initially-prepared luminophore was very close in energy with a charge-separated state produced by electron transfer^40,41^. In these cases, reversible electron transfer, and not energy transfer, is responsible for the equilibration process (and a charge-separated state, and not a triplet state of an organic chromophore, acts as the energy reservoir), but the above discussion on prompt and delayed components of the luminescence decays is unchanged.

To obtain luminescent (formally, fluorescent) Fe(III) compounds exhibiting excited-state equilibration, we synthesized two derivatives of **1** containing pyrene subunits, i.e. compounds **2** and **3** (see structural formulas in Fig. 1). The selection of pyrene chromophores was based on the close energy between the fluorescent doublet ligand-to-metal charge transfer (^2^LMCT) state of **1**, assumed to lie at 582 nm^16^, and the lower-lying triplet state of pyrene (^3^π−π*), known to be around 598 nm^33,42^.

The ^2^LMCT emissions of both **2** and **3** exhibit biphasic decays, with shorter-lived components corresponding to excited-state equilibration and longer-lived components corresponding to the lifetime of the equilibrated state. The lifetimes of **2** and **3** are increased with respect to that of **1** (at least for the longer-lived component) reaching 3.1 and 6.5 ns in **2** and **3**, respectively, in dichloromethane fluid solution at room temperature.

Here, we show a rather unusual behavior, not yet reported for earth-abundant metal complexes: the prolongation of the luminescence lifetime of the studied Fe(III) complexes is due to excited-state equilibration involving the emissive ^2^LMCT and a charge-separated state, with electron transfer rather than energy transfer driving the equilibrium.

## Results and discussion

**Fig. 2:**
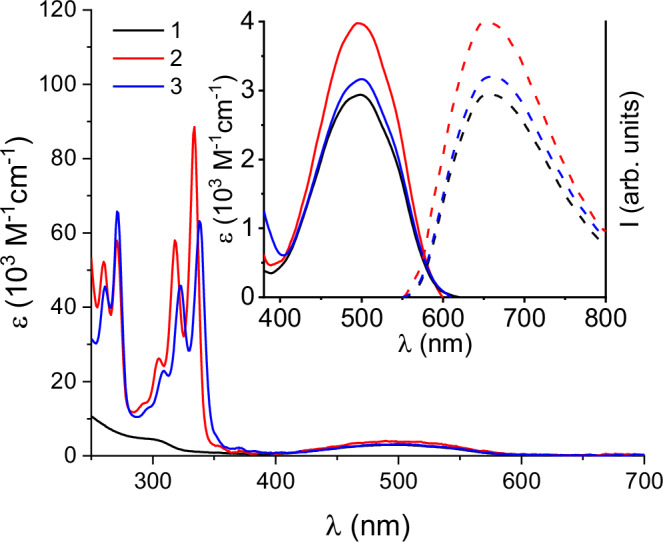
Absorption and emission spectra of 1–3 in acetonitrile at room temperature. Absorption spectra of **1–3** in acetonitrile fluid solution at room temperature and (inset) magnified absorption spectra in the visible (solid line) and emission spectra (dashed, differentially scaled for better visualization) of **1–3**. Colors identify the various compounds, as indicated in figure. Source data are provided as a Source Data file.

**Fig. 3:**
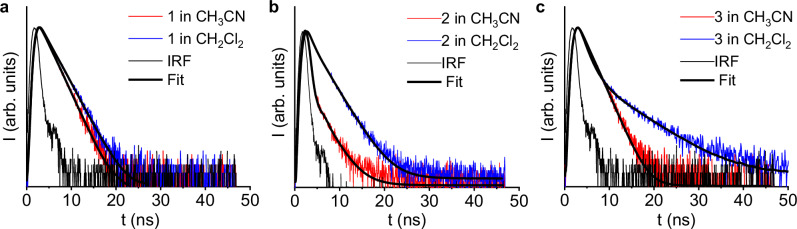
Luminescence decays of 1–3 in CH_2_Cl_2_ and CH_3_CN in fluid solution. Luminescence decays and fittings of the investigated complexes in dichloromethane (blue) and acetonitrile (red) at room temperature. Panels **a**, **b**, and **c** refer to compounds **1**, **2**, and **3**, respectively. Complex **2** in both solvents and complex **3** in dichloromethane are fitted by a biexponential, data in Table 1. Excitation wavelength, 456 nm. Emission wavelength, 670 nm. IRF is the instrumental response function. Source data are provided as a Source Data file.

Detailed synthesis and characterization of **2** and **3** are reported in the Supplementary Figs. 1–33. The absorption and emission spectra of **2** and **3** (and of **1**, for comparison purposes) in acetonitrile (AN) at room temperature (RT) are shown in Fig. 2. The absorption spectra in the visible are dominated by spin-allowed LMCT transitions; for **2** and **3**, intense structured absorption features in the UV region are due to spin-allowed transitions centered in the pyrene subunits^42^. At any excitation wavelength, only a single emission is present for **1–3**, which is identical in shape for all the compounds. Emission is slightly blue shifted on passing from CH_3_CN to CH_2_Cl_2_ solution for all the compounds, as usually expected for CT emissions (see Supplementary Fig. 34). The luminescence decays of **2** and **3** in CH_2_Cl_2_ at RT are biphasic (Fig. 3), whereas in CH_3_CN only **2** exhibits a biexponential decay. In EtOH/MEOH 4:1 (v/v) rigid matrix at 77 K, **2** and **3** exhibit a somewhat structured emission, still very similar in shape one another (Supplementary Fig. 35). Luminescence quantum yields of **3** in both CH_3_CN and CH_2_Cl_2_ at RT are independent of excitation wavelength within the range 390–520 nm and are about 0.02, whereas for **2** are two orders of magnitude smaller, in both solvents. Comparison between absorption and excitation spectra of **2** and **3** in both acetonitrile and dichloromethane shows that a fraction of the light absorbed by the pyrene subunits at energy higher than 350 nm does not contribute to ^2^LMCT emission (Supplementary Fig. 36), particularly in acetonitrile, and quantum yields are correspondingly lower when the pyrene singlet state is directly excited. Table 1 collects all the relevant spectroscopic and photophysical data of **2** and **3**, as well as some relevant data of **1**. Redox data of **1–3** in AN and 1,2-dichloroethane (DCE) are reported in Supplementary Tables 1, 2, cyclic and differential pulse voltammetry experiments of **2** and **3** in acetonitrile are shown in Supplementary Figs. 37, 38 and differential pulse voltammetry of **2** and **3** in DCE are shown in Supplementary Fig. 39. In particular, the reduction process (Supplementary Tables 1, 2) around −0.78 V vs SCE is assigned to the Fe(III)/Fe(II) process, the oxidation process in the range +0.64–+0.73 V vs SCE is assigned to the Fe(IV)/Fe(III) oxidation, the oxidation processes occurring in the range +1.2–1.6 V vs SCE are attributed to pyrene-based oxidation and the process around +2.28 V (only visible in AN, because of limitation in the potential window of DCE) is assigned to oxidation of the methylimidazole subunits.

**Table 1:** Absorption and luminescence data

	absorption	Luminescence, CH_3_CN			Luminescence, CH_2_Cl_2_		
	λ_max_, nm (ε, M^−1^cm^−1^)^a^	λ_max_, nm	τ, ns	Φ	λ_max_, nm	τ, ns	Φ
**2**	501 (3800)	655	0.3 (86%) 2.8 (14%)^b^	0.0004	645	0.8 (22%) 3.1 (78%)^b^	0.0007
**3**	501 (3200)	655	2.0	0.022	645	1.5 (79%) 6.5 (21%)^b^	0.019
**1**	501 (2950)	655	2.0	0.020	645	2.4	0.021

Absorption and luminescence data of **1–3** in fluid solution at room temperature. Air-equilibrated and nitrogen-saturated samples exhibit the same emission lifetimes and quantum yields. Emission data are independent of excitation wavelength in the excitation range 390–520 nm.

^a^Only the lowest-energy band maximum is reported.

^b^Biexponential decay; the percentage of each lifetime in the biexponential decays is given in parenthesis.

The absorption spectra and redox behavior of **2** and **3** clearly indicate that only weak interaction occurs between the pyrene and the Fe(III) complex subunits, with each subunit exhibiting localized excited and redox states. The ^2^LMCT excited state level of **2** and **3** in acetonitrile at RT is estimated to be similar to that of **1**, reported at 582 nm (2.13 eV^16^), whereas the energy of the ^3^π−π* pyrene state in **2** and **3** is approximated by the highest-energy features of the 77 K emission of **A** and **B**, that is 591 (2.10 eV) and 601 nm (2.06 eV), respectively (Supplementary Fig. 35). This picture is qualitatively consistent with the vertical excited states computed for compounds **1,** **2** and **3** at TD-DFT level (Supplementary Discussion – Part 2). Therefore, both experimental and computational data suggest that excitation of the ^2^LMCT state of **2** and **3** could lead to population of the almost isoenergetic triplet state of pyrene by energy transfer (by the above-mentioned values, energy transfer driving forces in acetonitrile are estimated to be 0.03 and 0.07 eV for **2** and **3**, respectively). At TD-DFT level the presence of low-lying quartet states is also evidenced, but their contribution to the doublet excited state dynamics is expected to be small due to their significant energy gap. Indeed, the intrinsic decay rate constant of the pyrene triplet state in solution is quite slow, even when accelerated by the proximity of a heavy atom (of the order of 10^6 ^s^−1^ or slower^43,44^), so back energy transfer can compete with decay to the ground state for pyrene triplet states, for small energy difference between the involved states, as in the present case. Noteworthy, the ^2^LMCT state in **2** and **3** can also be quenched by reductive electron transfer by the pyrene subunits, since electron transfer from pyrene (E_ox_ at +1.35 V and +1.32 V for **2** and **3** in AN, respectively, see Supplementary Table 1) to the excited iron subunit is thermodynamically allowed by ca. 0.01 eV (in **2**) and 0.02 eV (in **3**) in AN. Due to the practically isoenergetic ^2^LMCT state and the charge-separated pyrene^+^-Fe(II) species, reversible electron transfer is also possible and an electron transfer-driven equilibrated state could be formed^40,41^. The energy of the ^2^LMCT state of **2** and **3** in CH_2_Cl_2_ or DCE increases to 2.16 eV (see Supplementary Fig. 40) and, assuming the energy of the triplet state of the pyrene subunits unaffected by the solvent, the driving forces for energy transfer processes increase accordingly, however, remaining relatively low (0.06 eV for **2** and 0.10 eV for **3**) to allow excited-state equilibration. Redox data in DCE exhibit pronounced differences with respect to AN data, particularly for the pyrene-based oxidation (E_ox_ at +1.33 V and +1.23 V for **2** and **3**, respectively, Supplementary Table 2) and this leads to a larger driving force for electron transfer quenching in **2** (0.06 eV, to be compared to 0.01 eV in AN) and particularly in **3** (0.13 eV, to be compared to 0.02 eV in AN). According to the above considerations, the faster component of the luminescence decay in AN of **2**, having a lifetime of 0.3 ns (Table 1), can be assigned to the residual emission from the initially populated ^2^LMCT state (the “prompt” emission). Comparison with the 2 ns lifetime of **1** in the same experimental conditions^16^ suggests that the ^2^LMCT state in **2** is partially quenched, with a quenching rate constant of 2.8 ×10^9^ s^−1^, by: (i) reversible energy transfer to the closely-lying triplet state of pyrene in **2**, lying at 591 nm, or (ii) reductive electron transfer from pyrene (details on calculation are reported in Supplementary Discussion). The longest-lived component of the emission of **2** (2.8 ns, Table 1) is attributed to the lifetime of the equilibrated state (the delayed emission). In AN solution, the luminescence of **3** is monoexponential (Table 1): the larger distance between the pyrene subunits and the metal complex in **3** with respect to **2** (see structural formulas in Fig. 1) – implying a smaller electronic coupling between subunits - apparently makes the quenching process too slow to compete with the direct decay of the ^2^LMCT state of **3** to the ground state (5 ×10^8^ s^−1^ in AN, identical to the decay of **1**), although driving force in **3** could be slightly more favorable, for both energy and electron transfer quenching processes.

The situation is different in CH_2_Cl_2_, where the luminescence decay of ^2^LMCT state is biexponential for both **2** and **3** (see Table 1 and Fig. 3), probably due to thermodynamic reasons (together with, for electron transfer, reduced reorganization energy in apolar solvents, which influences the kinetics). The more interesting results are exhibited by **3**: the shorter component, assigned to the prompt emission from the initially populated ^2^LMCT state, has a lifetime of 1.5 ns, indicating that the quenching (equilibration) rate constant is about 3 ×10^8^ s^−1^ (see Supplementary Discussion), competitive with the intrinsic decay of **3** in this solvent (4.2 ×10^8^ s^−1^, from the luminescence lifetime of **1**, that is 2.4 ns). Luminescence lifetime of the equilibrated state is 6.5 ns, so showing that the luminescence of **3** in dichloromethane is about 2.7 times prolonged with respect to **1**, by taking advantage of the excited-state equilibration approach; therefore, this approach can efficiently be used to prolong Fe(III) complexes emission, without modifying the coordination environment of the metal chromophore or significantly losing emission quantum yield (Φ in **1** and **3** are 0.021 and 0.019, respectively, Table 1). It is here useful to clarify the meaning of “prolonged emission” we used in this paper. When weighted values of shorter- and longer-lived emission components are considered, the average lifetimes of the emission do not appear to be longer than that of **1**: however, the longer-lived emission component clearly shows that the emission of the ^2^LMCT state can be prolonged due to the excited-state equilibration. Ratio between prompt and delayed emissions must be optimized (for example, by increasing the energy gap between the states so favoring the quenching process versus prompt emission, but care is needed to still allow for thermal equilibration between the relevant states) for obtaining a larger effect.

As far as compound **2** in CH_2_Cl_2_ is concerned, the equilibration rate constant – related to the lifetime of the shorter component of the luminescence – is 8.0 ×10^8^ s^−1^. However, the emission lifetime of the longer-lived component, assigned to the equilibrated state, is only increased to 3.1 ns.

TDDFT computational studies have been performed on **1–3** and on the free ligands phtmeimb, **A**, and **B** (see Supplementary Discussion, Computational Study section, Supplementary Tables 3–11, Supplementary Figs. 45–61). Due to the complexity of the systems, more detailed TDDFT computational studies have focused only on compound **2** and in the identification of a possible equilibration between a ^2^LMCT state and a triplet pyrene-centered state. For compound **2**, it was possible to identify two relaxed excited states corresponding respectively to a ^2^LMCT state and a triplet pyrene differing by only 0.1 eV (Supplementary Fig. 47). Though the energy of both these states is overestimated with respect to experimental emission energies (consistently with the overestimation of the absorption and emission energies normally obtained with the level of theory chosen), overall the calculations confirm that an equilibration between these two excited states could be correlated with the observed prolonged luminescence. Noteworthy, bimolecular doublet-triplet energy transfer (DTET) from earth-abundant metal complexes to organic chromophores (although not generating equilibrated states) has been reported^45,46^.

A significative difference between **2** and **3** (and **1**) is the emission quantum yield of **2**, which is reduced by two orders of magnitude both in diluted CH_3_CN or CH_2_Cl_2_ solution (concentration about 1 ×10^−5 ^M in both cases) with respect to those of **3** and **1** (Table 1). Concentration dependence of the luminescence of **2** and **3** shines light on this difference: actually, the quantum yield of **2** strongly decreases on increasing concentration in the range 1–170 μM (Supplementary Fig. 41). Interestingly, luminescence lifetimes are not affected by concentration changes of **2**. These results suggest static quenching and aggregation of **2** even at low concentration, with the aggregated form of **2** essentially not emissive, whereas LMCT emission only comes from the non-aggregate species. On the contrary, the luminescence quantum yield of **3** is not affected by concentration in the range 1–220 μM (Supplementary Fig. 42).

**Fig. 4:**
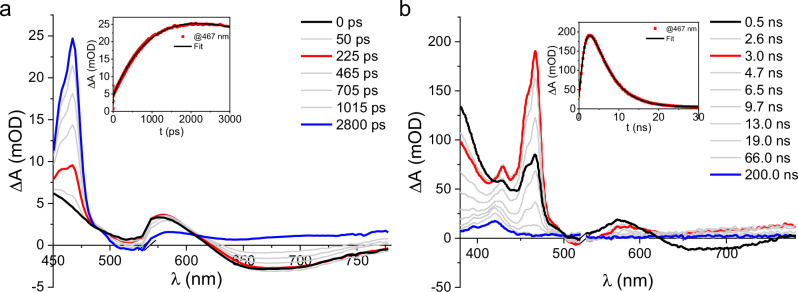
Transient absorption spectra and decay fittings of 3 in DCE. Panel **a**: Pump-probe experiment at shorter timescale (pump: 550 nm). In the inset, fitting of the rise at 467 nm (time constant, 1.2 ns). Panel **b**: Experiment on longer timescale (excitation, 532 nm) with an extended wavelength region. In the inset, the decay at 467 nm is shown (decay time constant: 5.9 ns). Source data are provided as a Source Data file.

The transient absorption spectra (TAS) of **3** in AN (pump at 550 nm, Supplementary Fig. 62) are essentially identical to the TAS of the ^2^LMCT state of **1**, exhibiting a transient absorption in the range 520–620 nm (which hides the LMCT bleaching) and stimulated emission in the 620–800 nm range^16^. According to luminescence data, TAS shows that no quenching of the ^2^LMCT state occurs in this experimental condition, and the ^2^LMCT state directly decays to the ground state, as also indicated by the isosbestic point at ΔA zero (decay time constant, 1.8 ns, in good agreement with the 2.0 ns emission lifetime). In DCE (pump-probe spectroscopy in CH_2_Cl_2_ is complicated by technical problems, so we used DCE as the solvent for pump-probe experiments), the initial LMCT spectrum of **3** evolves with a time constant of 1.2 ns, exhibiting an intense band peaking at 467 nm and a less intense, broad absorption in the 580–800 nm range (Fig. 4a); both features are characteristic of the pyrene radical cation^47^. Nanosecond flash photolysis (excitation, 532 nm) allowed us to investigate a larger spectral region, where also an intense transient band appears at λ < 400 nm, which reminds the typical Fe(II) absorption, known to peak around 370 nm (Fig. 4b)^16^. Therefore, TAS changes strongly indicate that the ^2^LMCT state evolves towards the formation of a pyrene^+^-Fe(II) charge-separated (CS) state by reductive electron transfer. This charge-separated state decays with a rate constant of about 5.9 ns (Fig. 4b). Noteworthy, the time constant of the formation of the charge-separated state (1.2 ns) is in fair agreement with the shorter component of the luminescence decay (1.5 ns), so formation of the charge-separated state evidenced by TAS is assumed to correspond to the prompt LMCT emission decay of **3**; the decay of the pyrene^+^-Fe(II) state (5.9 ns) is in fair agreement with the longer component of the LMCT emission (6.5 ns), supporting the longer-lived LMCT emission as powered by the CS state, with equilibration between the two excited states. These results indicate that electron transfer and not energy transfer leads to excited state equilibration in **3**. This is the first reported example showing that reversible electron transfer can effectively prolong luminescence lifetime of earth-abundant metal complexes, so opening alternative strategies for long-lived luminescence of such complexes. To the best of our knowledge, it is also one of the rare cases of excited-state equilibration driven by electron transfer in metal complexes, when also precious metal complexes are considered^40^. Our results also imply a relatively slow charge recombination to the ground state of the charge-separated state, not surprising when the energy level of this latter state is considered (over 2.00 eV, so that the charge recombination process should occur in a strongly inverted region).

It could be noted that in nanosecond flash photolysis spectra of **3** in DCE (Fig. 4b), a transient absorption peak at about 415 nm is also present upon CS decay; this peak is typical of the transient absorption of pyrene triplet states^48^ (see also Supplementary Fig. 64, in which the transient absorption spectrum of **B** ligand is shown). More details will be discussed by global kinetic analysis (see later). However, the decay of the 415 nm peak is long lived (about 400 ns, see later), so apparently it is not involved in the excited state equilibration leading to the 6.5 ns prolonged LMCT emission.

As far as the pyrene-to-*Fe(III) electron transfer in DCE is concerned, a reorganization energy of 0.92 eV can be estimated (Supplementary Discussion - Part 1), leading to an activation energy of 0.17 eV, by applying the Marcus quadratic equation^49^. When the nonadiabatic (weak coupling) limit is assumed^50^, a value of 28 cm^−1^ is estimated for H_AB_, the matrix element for the donor-acceptor electronic coupling (Supplementary Discussion – Part 1), a reasonable value for moderate electron transfer rates (of the order of 10^8 ^s^−1^) with relatively small driving forces (0.13 eV).

**Fig. 5:**
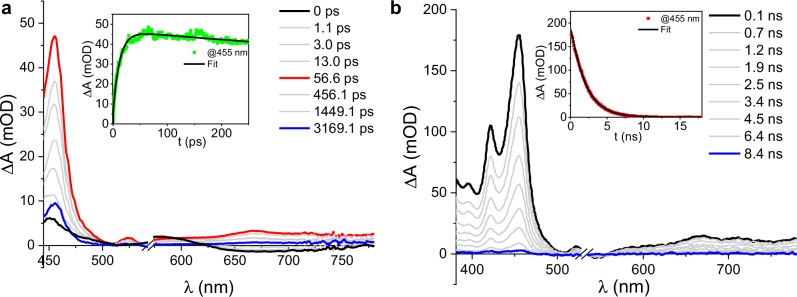
Transient absorption spectra and decay fittings of 2 in DCE. Panel **a**: Pump-probe experiment at shorter timescale (pump: 550 nm). In the inset, fitting of the rise and successive decay at 455 nm (time constant of the rise: 13 ps; time constant of the decay: see panel (**b**). Panel **b**: Experiment on longer timescale (excitation, 532 nm) with an extended wavelength region. In the inset, the decay at 455 nm is shown (decay time constant: 2.0 ns). Source data are provided as a Source Data file.

A note of caution is needed on studying the transient absorption spectroscopy of **2**: in fact, most of **2** is present in an aggregate form in solution (assuming the reduction of the emission quantum yield is due to formation of the non-emissive aggregate form of **2**, at least 90% of this species is already aggregated in dilute solution used for luminescence experiments, and this percentage is even larger at the higher concentrations needed for transient absorption spectroscopy). Therefore, the results obtained by transient absorption spectroscopy for **2** should be attributed to the (non-luminescent) aggregate form and could offer only limited comparison with the luminescence data, which are due to the isolated species. Nevertheless, fs pump-probe experiment of **2** in DCE (pump at 550 nm) indicates that the initially prepared ^2^LMCT state evolves similarly than in **3**, with formation of pyrene radical cation, which successively decays to the ground state (Fig. 5a). Nanosecond flash photolysis evidences also a transient absorption at λ < 400 nm, typical of the Fe(II) absorption, so indicating the formation of the pyrene^+^-Fe(II) charge-separated state upon reductive electron transfer (Fig. 5b), recalling what occurs in **3**. However, time constants of the processes are different than those that could be expected based on luminescence data: formation of CS occurs with a time constant of 13 ps and this state decays with a rate constant of 2.0 ns. Both processes are therefore faster than the respective analogous processes inferred by luminescence of **2**, which are assigned to the isolated species. In fact, in isolated **2** the reductive electron transfer leading to formation the pyrene^+^-Fe(II) CS state would correspond to the time constant of the prompt emission, that is about 800 ps (Table 1), and the decay of the charge-separated state should correspond to the longer-lived emission component due to the equilibrated state, that is 3.1 ns. Dependence of intramolecular electron transfer rate constants on aggregation is common and is usually attributed to structural modification of the systems affecting electronic coupling and/or increased delocalization of cation or anion subunits, even in absence of any spectroscopic signatures^51,52^. This is suggested to be the case for **2**. The faster formation of the charge-separated state of **2** in DCE would probably eliminate the “prompt” emission from the LMCT state and the reduced lifetime of the charge-separated state in the aggregate state could make reversible electron transfer less efficient, so favoring direct radiationless decay to the ground state from the charge-separated state and justifying the absence of emission of the aggregate species.

The situation is qualitatively similar for **2** in AN (Supplementary Fig. 63). Pyrene radical cation is formed from ^2^LMCT with a time constant of 23 ps, and the pyrene radical cation disappears with a time constant of 138 ps. Because of the quite short decay processes, nanosecond flash photolysis is useless in this case, so formation of Fe(II) cannot be registered, however it can be reasonably assumed that pyrene^+^-Fe(II) CS species is also formed in these experimental conditions. As in DCE experiments, also in AN the time constants of the various decay processes recorded by transient absorption spectroscopy are significantly faster with respect to the corresponding ones which could be inferred by luminescence data (300 ps for the formation of the equilibrated state and 2.8 ns for its decay, see Table 1), since the transient absorption spectroscopy would be dominated by data referring to aggregate species and the luminescence data would refer to isolated **2**. The non-luminescent nature of the aggregated **2**, reflected in the reduced emission quantum yield of **2** in AN, can be explained as discussed above for **2** in DCE.

Since both electron and energy transfer can quench the ^2^LMCT state in **2** and **3** with comparable and relatively small driving forces (e.g., 0.10 eV and 0.13 eV for energy and electron transfer in DCE in **3**, respectively, whereas the same driving force, 0.06 eV, is calculated for energy and electron transfer in **2** in the same solvent), it should be discussed why the electron transfer route dominates the quenching process leading to excited-state equilibration. In fact, energy transfer to pyrene triplet state in **2** and **3** can only occurs by Dexter mechanism, and Dexter energy transfer electronic coupling is expected to be significantly smaller than the electronic coupling for electron transfer^53,54^. Therefore, we attribute the dominating role of the electron transfer quenching with respect to the energy transfer quenching as mainly due to electronic factors. Interestingly, a recently reported ^2^LMCT to triplet anthracene process, published during the revision of the present paper and formerly attributed to doublet-to-triplet energy transfer^55^, has later been clarified to occur by two sequential intramolecular electron transfer processes^56^.

**Fig. 6:**
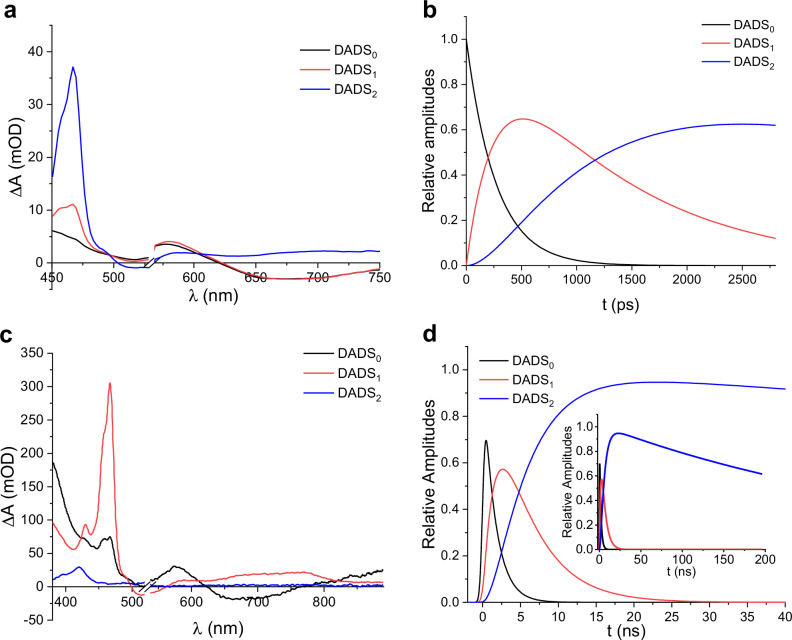
Global kinetic analysis of 3 in DCE. Panel **a**: Decay-associated difference spectra (DADS) of **3** in DCE, obtained from the pump-probe experiment shown in Fig.4a. DADS_0_ is the initial spectrum, having a decay time constant of 238 ± 53 ps; DADS_1_ has a decay time constant of 1.29 ± 0.20 ns; DADS_2_, the final spectrum, has a decay time constant of 5.52 ± 0.90 ns. Panel **b**: Relative populations of the individual excited states reported in panel (**a**). Panel **c**: DADS of **3** in DCE, obtained from nanosecond flash photolysis experiment at longer times shown in Fig. 4b. DADS_0_ is the initial spectrum (decay time constant, 1.59 ± 0.57 ns); DADS_1_ corresponds to the fully-developed CS state (decay time constant, 5.62 ± 0.70 ns); DADS_2_ is the final spectrum (decay time constant, 391 ± 147 ns). Panel **d**: Relative populations of the individual excited states reported in panel (**c**). Source data are provided as a Source Data file.

**Fig. 7:**
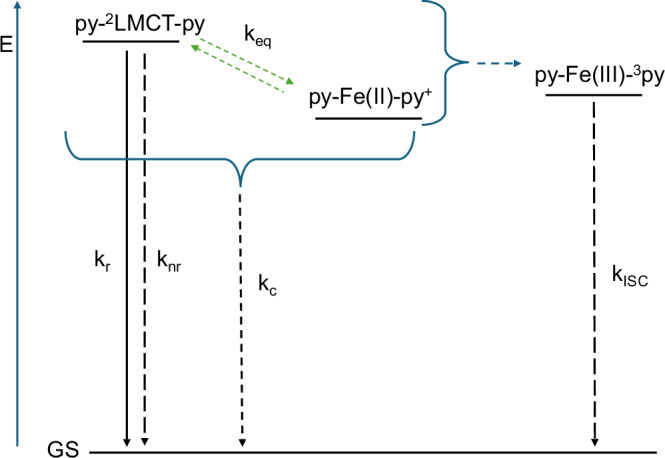
Representation of the excited state levels and decay processes occurring in 3 in DCE solution. Schematic representation according to luminescence, transient absorption spectroscopy (TAS) experiments and global kinetic analysis. ^2^LMCT is reported as py-^2^LMCT-py to evidence the presence of the pyrene (py) groups. Once excited, the ^2^LMCT level decays by its intrinsic radiative and radiationless routes to the ground state (GS) yielding emission (the “prompt” emission) and by electron transfer to the py-Fe(II)-py^+^ CS state (the biphasic nature of the electron transfer is neglected for simplicity reasons), giving arise to an equilibrated ^2^LMCT/CS level. Such an equilibrated state decays via thermally-activated emission from the ^2^LMCT level (the “delayed” emission, with a lifetime of about 6.5 ns), by charge recombination to GS and (in a small percentage) by formation of pyrene triplet state. The pyrene triplet state decays by intersystem crossing to the ground state with a time constant of about 400 ns.

Global kinetic analysis can uncover some details that are hidden (or not clearly evidenced) in single wavelength kinetics, so we performed global kinetic analysis (GKA) on the transient absorption spectra of **3** in DCE and of **2** in acetonitrile and DCE. Indeed, GKA of **3** in DCE (Fig. 6) indicates two interesting features that were not easily revealed by single wavelength kinetics. First, evidenced by GKA in the ultrafast time regime, electron transfer from the pyrene to *Fe(III) subunit is biphasic (Fig. 6a, b): a decay-associated differential spectrum (DADS_1_) exhibiting an initial growth of the transient absorption typical of pyrene radical cation at 467 nm is obtained from DADS_0_ (the initial spectrum) with a time constant of 238 ± 53 ps; DADS_1_ evolves to the fully-developed transient absorption spectrum of the py^+^-Fe(II) CS state in 1.29 ± 0.20 ns (in fair agreement with the time constants of 1.2 and 1.5 ns obtained by single wavelength kinetics of transient spectrum and with the prompt emission component, respectively). The DADS_2_ finally obtained has a decay time constant of 5.52 ± 0.90 ns (in fair agreement with the single wavelength kinetics, 5.9 ns, and the delayed emission lifetime, 6.5 ns). Second, revealed by GKA in the ns timescale (Fig. 6c, d), the decay of the py^+^-Fe(II) CS decay (time constant, 5.62 ± 0.70 ns), formed from the initial transient spectrum with a decay time constant of 1.59 ± 0.57 ns in the flash photolysis experiments, leads to a transient spectrum having an absorption peak at about 415 nm, typical of the pyrene triplet state^48^ (see also Supplementary Fig. 64), which finally decays to the ground state on a much longer timescale (391 ± 147 ns, see Fig. 6c, d). This suggests that the equilibrated (^2^LMCT/CS) state, responsible for the delayed emission output of **3**, deactivates also via population of the triplet pyrene state. The delayed emission lifetime, therefore, is not due only to decay of the equilibrated (^2^LMCT/CS) state to the ground state, but also includes the decay process populating the pyrene triplet state. Whether the pyrene triplet state yields a further equilibration process with the (^2^LMCT/CS) state (so contributing to the emission output) cannot be excluded, but it would be hard to investigate since the percentage of such a three-level equilibrated state to the luminescence output would be extremely low and almost negligible (and in fact the luminescence decay is fitted by two components). A schematic representation of the excited-state decay processes of **3** in DCE is shown in Fig. 7.

**Fig. 8:**
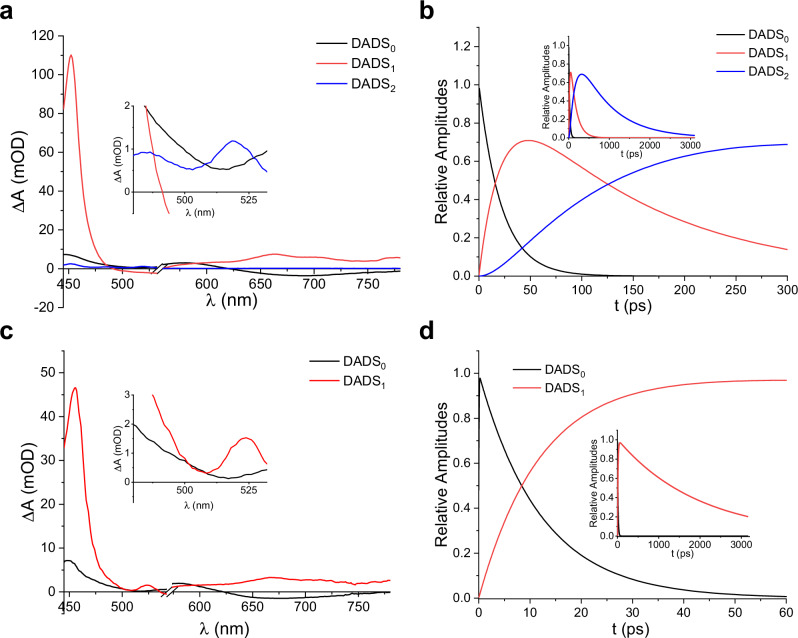
Global kinetic analysis of 2 in acetonitrile and in DCE. Panel **a**: decay-associated difference spectra (DADS) of **2** in AN, obtained from the pump-probe experiment shown in Supplementary Fig. S53. DADS_0_ is the initial spectrum, with a decay time constant of 22 ± 1 ps; DADS_1_ has a decay time constant of 140 ± 2 ps; DADS_2_, the final spectrum, has a decay time constant of 800 ± 113 ps. Panel **b**: relative populations of the individual excited states reported in panel (**a**). Panel **c**: DADS of **2** in DCE, obtained from data shown in Fig. 5a. DADS_0_ is the initial spectrum (decay time constant,12 ± 4 ps); DADS_1_ is the final spectrum (decay time constant, 1.97 ± 0.17 ns). Panel **d**: relative populations of the individual excited states reported in panel (**c**). Source data are provided as a Source Data file.

GKA of **2** in AN reveals a similar qualitative behavior (Fig. 8a, b): the initial DADS_0_, assigned to the initially formed ^2^LMCT state, decays with a time constant of 22 ± 1 ps to DADS_1_, which is attributed to the CS state. This latter state decays with a time constant of 140 ± 2 ps leading to DADS_2_, which shows characteristic features of the triplet pyrene state, in particular an absorption feature around 520 nm, which is a side band of the main 415 nm peak (see Supplementary Fig. 64)^48^. So, even for the aggregate **2** in AN, the triplet pyrene state contributes (at least in part) to the CS state decay. The pyrene triplet state finally decays to the ground state with a time constant of 800 ± 113 ps. This is by far a very short lifetime for a triplet pyrene, but it should be considered that (i) it involves an aggregate species and (ii) the closely lying CS state could play the role of a sink for the triplet pyrene decay. Supplementary Fig. 65 shows a schematic representation of the excited-state decay process of **2** in AN.

In DCE, the initial DADS_0_ of **2** evidenced by GKA decays with a time constant of 12 ± 4 ps and the DADS_1_ formed from DADS_0_ contains spectral features of both the charge-separated CS state (the peak around 467 nm) and of the pyrene triplet state (the peak around 520 nm) (Fig. 8c, d). Formation of pyrene triplet state, together with the CS state, from the initial ^2^LMCT of **2** in DCE was also suggested by the absorption peak at about 420 nm observed in nanosecond flash photolysis experiments (Fig. 5b). So, both states are formed from the initial ^2^LMCT state. The CS and pyrene triplet states decay together to the ground state, indicating that they are equilibrated, with a time constant of 1.97 ± 0.17 ns. A schematic representation of the excited-state decay processes of **2** in DCE is shown in Supplementary Fig. 66.

Summarizing, the photophysical properties of the new species **2** and **3** indicate the potential of the excited-state equilibration approach to achieve long-lived luminescence involving iron complexes. Differently from the usually reported excited-state equilibration, based on energy transfer between closely-lying excited states, in **2** and **3** it is reversible electron transfer, coupling the emissive ^2^LMCT state and a Fe(II)-pyrene^+^ charge-separated level, which drives the equilibration process leading to delayed emission. Besides being one of the very few examples of this behavior in general, this result shows that electron transfer-driven excited state equilibration can be successfully applied to earth-abundant metal complexes and introduces a still unexplored strategy to prolong luminescence lifetimes of earth-abundant metal complexes.

## Methods

### Synthesis and characterization

All the reactions, except metathesis reactions, have been carried out under an inert nitrogen atmosphere. Chromium(0) hexacarbonyl, boron trifluoride diethyl etherate, 2,2,6,6-Tetramethylpiperidine, n-butyllithium, chlorotrimethylsilane solution (2 M in cyclohexane), boron trichloride solution (1.0 M in methylene chloride), 1-methylimidazole, trimethylsilyl trifluoromethanesulfonate, lithium bis(trimethylsilyl)amide, 4-(trimethylsilyl)phenylboronic acid, 1,3-dicyclohexylimidazolium chloride and all dry solvents were purchased from Sigma Aldrich. Iron(II) bromide (ultra dry, 99.995%) was purchased from Thermo Scientific and bis(1,5-cyclooctadiene)nickel(0) (wax encapsulated) was purchased from TCI. The synthesis of 2-(trimethylsilyl)-pyrene (**2-tmspy**) and tri(methylsilyl)phenylboronic acid neopentyl ester (**tmspbanpe)** were performed following literature procedures^57,58^. Detailed synthetic procedures are provided in the SI.

### NMR spectra and elemental analyses

NMR spectra were recorded on a Varian 500 spectrometer, using deuterated solvents (purchased from Sigma-Aldrich) at 295 K and calibrated with the residual NMR solvent: CD_3_CN (1.94 ppm for ^1^H NMR spectra and 1.32 and 118.26 ppm for ^13^C NMR spectra) and DMSO-d_6_ (2.50 ppm for ^1^H NMR spectra and 39.52 ppm for ^13^C NMR spectra). Elemental analyses were performed with a FLASH 2000 Series CHNS/O analyzer (ThermoFisher Scientific).

### High-resolution mass spectra

Time-of-Flight Secondary Ion Mass Spectrometry (ToF-SIMS) analysis was performed by a IONTOF ToF-SIMS IV instrument (IONTOF GmbH) using a Bi^+^ analysis beam (25 keV, 0.7 pA), rastered over an area of 300 × 300 µm². The spectra were recorded in both positive and negative polarity. Mass resolution (m/Δm) @m/z = 28 (Si^+^ ion) was 8500.

### Absorption, emission and lifetimes measurements

UV/Vis absorption spectra were taken on a Jasco V-560 spectrophotometer. For steady-state luminescence measurements, a HORIBA Jobin Yvon Fluorolog-3 spectrofluorimeter was used, equipped with a Hamamatsu R928P photomultiplier tube. The spectra were corrected for photomultiplier response using a program purchased with the fluorimeter. For the luminescence lifetimes, the same instrument was used equipped with a HORIBA Delta-Hub module and HORIBA NanoLED solid-state pulsed diodes (<1.4 ns pulse width at 456 nm), using Horiba native software, DAS6 v.6.8, to fit decays. Fittings were made by using default parameters. Emission quantum yields for deaerated solutions were determined using the optically diluted method^59^. As luminescence quantum yield standard, we used [Ru(bpy)_3_]^2+^ (bpy = 2,2’-bipyridine)^60^.

### Electrochemical measurements

Cyclic voltammetry (CV) and differential pulse voltammetry (DPV) were made using an Autolab PGSTAT 12 potentiostat/galvanostat instrument controlled with GPES software (v. 4.9) and connected to an electrochemical cell with a three-electrode setup. The measurement apparatus is composed of a glassy carbon working electrode, a platinum wire counter electrode and an Ag/Ag^+^ electrode as reference electrode, using the Fc/Fc+ couple (+0.395 V vs. SCE) as internal reference. Measurements were performed in degassed (Ar) acetonitrile (purchased from Merck, spectroscopic grade) solutions of the desired species (0.5 mM) using 0.05 M tetrabutylammonium hexafluorophosphate (TBAPF_6_, Sigma Aldrich, electrochemical grade, ≥99.0%) as the supporting electrolyte.

### Transient absorption measurements

Time-resolved transient absorption experiments were performed using a pump-probe setup based on the Spectra-Physics MAI-TAI Ti:sapphire system as the laser source and the Ultrafast Systems Helios spectrometer as the detector. The output of laser beam was splitted to generate pump and probe beam pulses with a beam splitter (85 and 15%). The pump pulse (400 nm, 1–2 μJ) was generated with a Spectra-Physics 800 FP OPA and focused in the 2 mm quartz cuvette containing the sample. The probe beam was delayed with a computer-controlled motion controller and then focused into a 2-mm sapphire plate to generate a white light continuum (spectral range 400–800 nm). The white pulse is then overlapped with the pump beam in the cuvette containing the sample. The effective time resolution was about 200 fs, and the temporal chirp over the white-light 450–750 nm was ca. 150 fs; the temporal window of the optical delay stage was 0–3200 ps. To cancel out orientation effects on the dynamics, the polarization direction of the linearly polarized probe pulse was set at a magic angle of 54.7° with respect to that of the pump pulse. All the transient spectra shown in the present paper are chirp corrected. The time-resolved data were analyzed with the Ultrafast Systems Surface Explorer Pro software v. 4.3.

Nanosecond transient absorption experiments were performed with an EOS broadband pump-probe nanosecond Transient Absorption Spectrometer (Ultrafast Systems LLC). The pump pulses at λ = 532 nm (7 μJ) is generated with an actively Q-switched Nd:YAG laser with an integrated harmonics generator. The probe pulse (1 kHz, 0.5 ns pulse width), which was generated in a photonic fiber, was synchronized with the femtosecond amplifier. The measurements were carried out in-situ using 2 mm quartz cuvettes. The transient absorption spectra were analyzed using the CarpetView software v. 2.0 (Light Conversion).

Experimental uncertainties are as follows (unless otherwise stated): absorption maxima, 2 nm; molar absorption, 15%; luminescence maxima, 4 nm; luminescence lifetimes, 10%; luminescence quantum yields, 20%; transient absorption decay and rise rates, 10%; redox potentials, 10 mV.

### Computational methods

All DFT calculations were performed with the Gaussian16 program^61^. The LC-PBE functional^62^ was used in conjunction with the 6-311 + G basis set^63,64^, including d polarization functions on the nitrogen and boron atoms. Iron was described with the LANL2TZ+ basis set and the corresponding effective core potential (ECP)^65,66^. Implicit solvent effects (dichloromethane) were modeled using the polarizable continuum model (PCM)^67,68^. Further computational details are provided in the SI. The Cartesian coordinates of all optimized structures are collected in Supplementary Data 1. Supplementary Tables 4–11 are reported in Supplementary Data 2.

## Supplementary information

Supplementary Information

Description of Additional Supplementary Information

Supplementary Data 1

Supplementary Data 2

Transparent Peer Review file

## Source data

Source Data

## Data Availability

Source data are provided with this paper. All the data that support the findings of this paper are available via Figshare at 10.6084/m9.figshare.31833724. All data are available from the corresponding author upon request. Source data are provided with this paper.
