# Supplementary material for: Fe(III) complexes with prolonged luminescence lifetimes via excited-state equilibration promoted by reversible intercomponent electron transfer
Source: Nat Commun. 2026 Apr 11;17:5090. doi: 10.1038/s41467-026-71767-4 (PMC13247219; doi:10.1038/s41467-026-71767-4)
Supplement: Supplementary file 1 — Supplementary Information [file 41467_2026_71767_MOESM1_ESM.pdf]

Supplementary Information for

Fe(III) complexes with prolonged luminescence lifetimes via excited-state equilibration promoted by reversible intercomponent electron transfer

Salvatore Genovese, Ambra M. Cancelliere, Antonino Arrigo, Alessandro Auditore, Antonino Licciardello, Manuel Pedrón, Ilaria Ciofini, Fausto Puntoriero, Sebastiano Campagna

Corresponding authors: campagna@unime.it (SC); fpuntoriero@unime.it (FP)

Table of Contents

Synthetic procedures 3
NMR Spectra 10
High-resolution Mass Spectra 26
Additional spectra and data 36
Supplementary Discussion – Part 1: Luminescence and Photophysical Data 44
Supplementary Discussion - Part 2. Computational Study 47
Supplementary Transient Absorption Spectra 78
Supplementary References 82

Synthetic procedures

- Triaminechromium tricarbonyl, **$Cr(CO)_3(NH3)_3$**

Chromium hexacarbonyl (10 g, 45,44 mmol), potassium hydroxide (18 g, 320.80 mmol) and a degassed solvent mixture (ethanol/ n-butanol/ water, 35/5/2 v/v) and heated under reflux for 4 hours. The reaction mixture was cooled to room temperature and placed in an ice bath. A degassed aqueous solution of ammonia (85 mL) was added and after 2 hours of stirring, a yellow solid was isolated through a Schlenk frit (87 %).

- (Pyrene)chromium tricarbonyl, **$pyCr(CO)_3$**

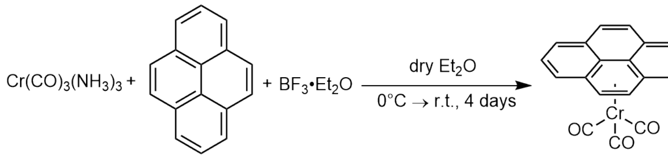

Supplementary Fig. 1. Synthetic scheme of **$pyCr(CO)_3$**.

In a two-neck flask charged with **$Cr(CO)_3(NH_3)_3$** (2.08 g, 11.11 mmol), pyrene (2.4 g, 11.87 mmol) and 60 mL of dry diethyl ether, boron trifluoride diethyl etherate (BF_3Et_2O, 45.4 mmol, 5.6 mL, 1.15 g/ml) was added dropwise at 0 °C. The reaction mixture was stirred at room temperature for 4 days and filtered with a Schlenk frit. The precipitate was dissolved in dichloromethane and extracted with an aqueous solution of HCl 1 M. The organic phase was dried over anhydrous magnesium sulfate, rotary evaporated and recrystallized in diethyl ether (45 %).

- Lithium 2,2,6,6-tetramethylpiperidide, **LiTMP**

To a solution of 2,2,6,6-Tetramethylpiperidine (3 mL, 17.6 mmol, 0.83 g/mL) in 25 mL of dry tetrahydrofuran was added a solution of n-butyllithium in cyclohexane 2 M (9 mL, 18 mmol) at -78°C. The resulting solution was stirred at room temperature for 30 minutes, obtaining a dark orange **LiTMP** solution.

- 2-(trimethylsilyl)-pyrene, **2-tmspy**

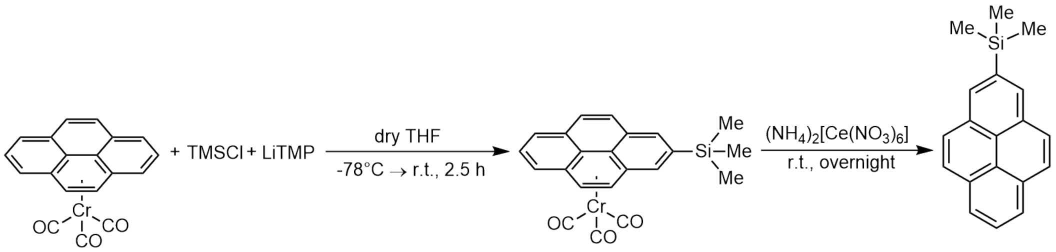

Supplementary Fig. 2. Synthesis of **2-tmspy**.

In a two-neck flask containing **$pyCr(CO)_3$** (1 g, 2.96 mmol) in 30 mL of dry tetrahydrofuran was added, at -78°C, a solution of chlorotrimethylsilane (**TMSCl**, 1.2 mL, 9.44 mmol, 0.856 g/mL) and then the freshly prepared solution of **LiTMP**. After stirring at -78°C for 1.5 hours, it was quenched with 50 mL of HCl 0.3 M, allowed to warm to room temperature, and then after 1 hour diluted with 50 mL of diethyl ether. To decomplex the desired product, a solution of cerium ammonium nitrate, 0.2 M in 25 mL of HCl 0.3 M were added and the resulting mixture was left under constant magnetic stirring overnight. The reaction mixture was

acidified with HCl 1 M until pH 1 and extracted with petroleum ether (3 x 100 mL). The resulting organic phase was washed with brine, dried over magnesium sulfate, and concentrated under reduced pressure. The pure compound was isolated by column chromatography on alumina using a hexane/ethyl acetate mixture (95:5 V/V) as eluent, with a yield of 50 %.

- 2-pyrenyl[tris(3-methylimidazol-1-ylidene)]borate bis(hexafluorophosphate), **pytmeimb, A**

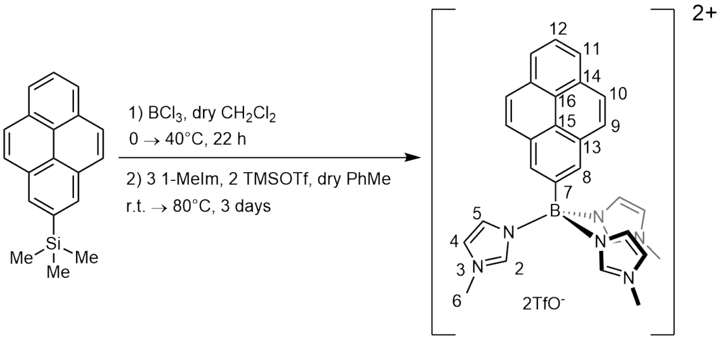

Supplementary Fig. 3. Synthesis of **A**.

To a solution of **2-tmspy** (400 mg, 1.46 mmol) in 3 mL of dry dichloromethane was added at 0°C a solution of boron trichloride 1 M in dichloromethane (4,5 mmol, 4.5 mL). After 1 h, the ice bath was removed, and the reaction mixture was stirred for 19 h, then the temperature was increased to 40°C and the reaction mixture furtherly reacted for 3 h. The solvent was removed under reduced pressure and then 4 mL of toluene and 600 µL of 1-methylimidazole (**1-MeIm**, 7.5 mmol, 1.03 g/mL) were added. After 15 minutes of stirring, a solution of trimethylsilyl trifluoromethanesulfonate (**TMSOTf**, 800 µL, 4.4 mmol, 1.23 g/mL) in 4.5 mL toluene was added dropwise and the reaction mixture was heated to 80°C. After 3 days, the flask contents were concentrated, solubilized in dichloromethane and extracted three times with water. The desired compound, which precipitates, was collected through filtration as trifluoromethanesulfonate salt. It was solubilized in the minimum amount of ethanol and ammonium hexafluorophosphate (233 mg, 1.43 mmol) was added. A white solid was isolated through filtration (61%).

^{1}H NMR (500 MHz, DMSO): δ (ppm) 8.66 (t, 3H, 1.8 Hz, H_2), 8.32 (d, J = 7.7 Hz, 2H, H_{11}), 8.22 (d, J = 9.0 Hz, 2H, H_{10}), 8.13 (d, J = 9.0 Hz, 2H, H_9), 8.10 (t, J = 7.7 Hz, 1H, H_{12}), 8.00 (s, 2H, H_8), 7.87 (t, J = 1.8 Hz, 3H, H_5), 7.55 (t, J = 1.8 Hz , 3H, H_4), 3.86 (s, 9H, H_6); 13C NMR (126 MHz, DMSO) δ (ppm) 139.78 (C_2), 130.79 (C_{14}), 130.34 (C_{15}), 129.38 (C_8), 127.75 (C_9), 127.39 (C_{10}), 126.54 (C_{12}), 125.17 (C_{11}), 124.69 (C_5), 124.15 (C_4), 123.57 ($C_{16,17}$), 35.5 (C_6).

Absorption spectra in acetonitrile solution (λ/nm , ε/$M^{-1}cm^{-1}$): 334 (39400), 318 (26600), 306 (11700), 292 (5700), 272 (32000), 261 (24300), 242 (75800).

HR-MS (ToF-SIMS, positive mode): m/z [**A** · CF_3SO_3]$^+$ = 607.1849 (Calculated: 607.1910).

HR-MS (ToF-SIMS, negative mode): m/z [**A** · 3CF_3SO_3]$^-$ = 905.0895 (Calculated: 905.0951).

- Bis{2-pyrenyl[tris(3-methylimidazol-1-ylidene)]borate}Fe(III) hexafluorophosphate, **[Fe(pytmeimb)$_2$](PF$_6$), 2**

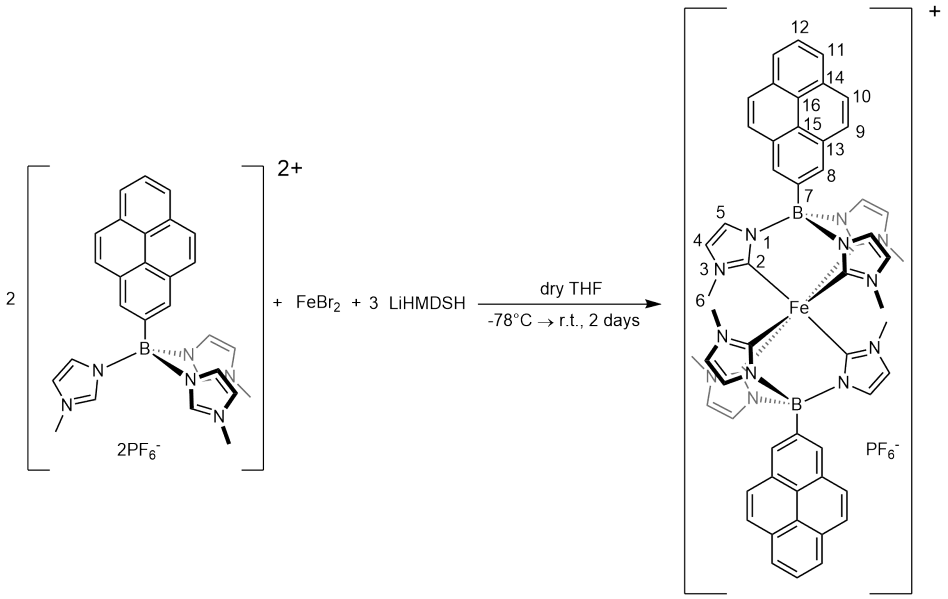

Supplementary Fig. 4. Synthetic scheme of **2**.

(pytmeimb)(PF$_6$)$_2$ (390 mg, 0.52 mmol) was dried under vacuum at 80°C overnight. Dry THF (10 mL) was added at room temperature and then the mixture was cooled at -78°C. A solution of lithium bis(trimethylsilyl)amide 1 M in THF (**LiHMDS**, 2 mL, 2 mmol) was added dropwise and after 40 min of constant magnetic stirring, an anhydrous, freshly prepared, iron(II) bromide solution (prepared sonicating iron(II) bromide (54 mg, 0.25 mmol) in 5 mL of dry THF for 30 minutes in a Schlenk flash) was introduced via cannula. The reaction mixture was allowed to slowly reach room temperature and then was stirred at room temperature for 2 days. Solvents were removed under reduced pressure and the residue was solubilized in acetonitrile and filtered. The resulting solid, solubilized in ethanol, was treated with an excess of ammonium hexafluorophosphate inducing the formation of a red precipitate. Then it was eluted through a size-exclusion column using Sephadex G-10 as stationary phase and dichloromethane as eluent. The compound, solubilized in dichloromethane, was purified on alumina PLC (1.2 mm thickness) using toluene/acetonitrile, 3:1 v/v, as eluent. The product of interest was eluted as a second band, recovered with dichloromethane extractions, concentrated and reprecipitated in diethyl ether. The pure compound was obtained as a dark pink solid with a 15 % yield (43.5 mg).

^{1}H NMR (500 MHz, CD_3CN): δ (ppm) 15.66 (s, 4H, H_8), 10.41 (d, J = 8.1 Hz, 4H, H_9), 9.38 (d, J = 8.1 Hz, 4H, H_{10}), 9.13 (d, J = 7.8 Hz, 4H, H_{11}), 8.77 (t, J = 7.8 Hz, 2H, H_{12}), 5.14 (s, 18H, H_6), 1.66 (s, 6H, H_5), -12.31 (s, 6H, H_4); ^{13}C NMR (126 MHz, CD_3CN): δ (ppm) 137.34 (C_8), 135.22 (C_{13}), 133.86 (C_{14}), 131.44 (C_9), 129.82 (C_{10}), 128.25 (C_{12}), 126.97 (C_{11}), 49.23 (C_4), 14.76 (C_5), -28.65 (C_6).

Absorption spectra in acetonitrile solution (l/nm , e/$M^{-1}cm^{-1}$): 501 (3800), 334 (88500), 318 (58000), 305 (26200), 292 (14000), 271 (57900), 260 (52200).

Elemental analysis calculated (%) for $C_{56}H_{48}B_2F_6FeN_{12}P \cdot H_2O \cdot CH_3CN \cdot CH_2Cl_2$: C 56.44, H 4.42, N 14.50, found (%): C 56.08, H 4.15, N 14.41.

HR-MS (ToF-SIMS, positive mode): m/z [**2**]$^+$ = 966.3546 (Expected: 966.3660).

HR-MS (ToF-SIMS, negative mode): m/z [**2** · 2PF$_6$]$^-$ = 1256.3021 (Expected: 1256.2943).

- <u>2-methoxymethylpyrene, **2-meomepy**</u>

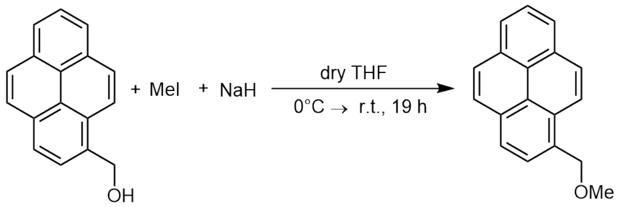

Supplementary Fig. 5. Synthetic scheme of **2-meomepy**.

Sodium hydride 60 % w/w in mineral oil (260 mg, 6.50 mmol) was added in 20 mL of dry tetrahydrofuran and then 1-pyrenemethanol (1 g, 4.31 mmol) was introduced at 0°C. The bath was removed and after 1 h of stirring, methyl iodide (400 µL, 2.28 g/mL, 6.4 mmol) was poured to the dispersion. After 19 hours, the reaction was quenched with about 10 mL of methanol and concentrated under reduced pressure. The residue was solubilized in acetone and filtered. The dried filtrate, which forms an orange oil, was chromatographed on silica using petroleum ether/ethyl acetate (95:5) as eluent to isolate the desired product (95 % yield).

- Tri(methylsilyl)phenylboronic acid neopentyl ester, **tmspbanpe**

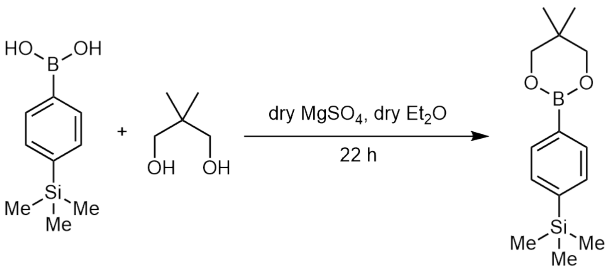

Supplementary Fig. 6. Synthesis of **tmspbanpe**.

Three freeze-pump-thraw cycles were performed in a two-neck flask containing an excess anhydrous magnesium sulfate (1.3 g, 10.80 mmol), 4-(trimethylsilyl)phenylboronic acid (1 g, 5.15 mmol), neopentyl glycol (542 mg, 5.20 mmol) and 15 mL of dry diethyl ether. After 22 h, the reaction mixture was loaded onto a plug of silica gel, washed with copious amounts of diethyl ether and concentrated under reduced pressure, obtaining an off-white solid (1.33 g, 99 % yield).

- 4-[(1-pyrenyl)methyl]phenyl[tris(3-methylimidazol-1-ylidene)]borate} bis(hexafluorophosphate), **pyphtmesi**

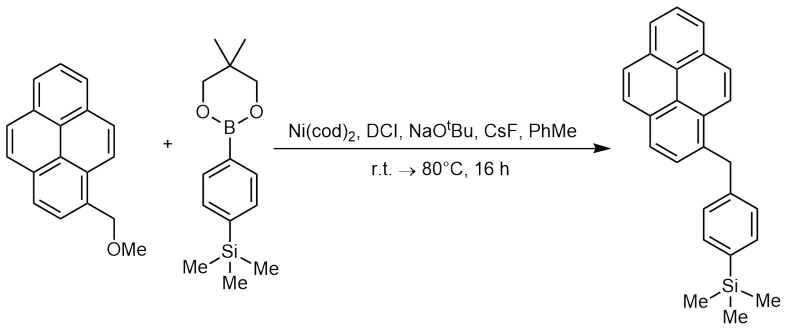

Supplementary Fig. 7. Synthesis of **pyphtmesi**.

In glove box, a two-neck flask was charged with 5 paraffin capsules of bis(1,5-cyclooctadiene)nickel(0) (about 0.25 mmol), 1,3-dicyclohexylimidazolium chloride (**DCI**,

0.50 mmol, 134 mg), sodium tert-butoxide (0.63 mmol, 60 mg) and 4.5 mL toluene. After about 3 min, cesium fluoride (5.00 mmol, 760 mg), **tmspbanpe** (3.75 mmol, 984 mg) and **2-meomepy** (2.47 mmol, 610 mg) were added sequentially. The flask was removed from the glove box and heated under reflux for 16 h. The reaction crude was placed in a Soxhlet extractor and washed with methanol MeOH for 24 h. The desired product was isolated through PLC on silica using hexane/dichloromethane (3:1 v/v) as mobile phase and diethyl ether for the extraction (260 mg, 32%).

- 4-(pyrenylmethyl)phenyl[tris(3-methylimidazol-1-ylidene)]borate} bis(hexafluorophosphate), **pyphtmeimb, B**

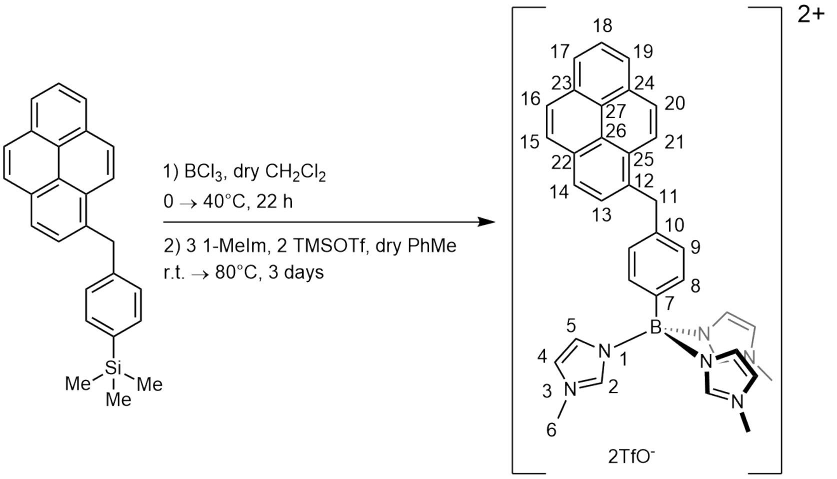

Supplementary Fig. 8. Synthetic scheme of **B**.

The synthetic procedure was the same as that of **pytmeimb,** using 250 mg of **pyphtmesi** (0.714 mmol) in 1.5 mL of dichloromethane, 2,5 mL of boron trichloride 1 M in dichloromethane (2,5 mL), 2 mL of toluene, 300 µL of 1-methylimidazole (**1-MeIm**, 3.8 mmol, 1.03 g/mL) were added. After 15 minutes of stirring, a solution of trimethylsilyl trifluoromethanesulfonate (**TMSOTf**, 400 µL, 2.2 mmol, 1.23 g/mL) in 2 mL toluene was added dropwise and the reaction mixture was heated to 80°C. After 4 days, the solvent was removed. The residue was solubilized in dichloromethane and extracted 3 times with water (3x30 mL). The organic phase was anhydrified with magnesium sulfate and brought to dryness, obtaining a dark brownish oil. This was solubilized in the minimal amount of dichloromethane and allowed to precipitate in diethyl ether. The precipitate was filtered and washed with plenty of diethyl ether, obtaining the ligand as trifluoromethanesulfonate. The solid was solubilized in ethanol and treated with ammonium hexafluorophosphate (174 mg). The desired product, as hexafluorophosphate salt, was isolated through filtration and washed with ethanol and diethyl ether (45%).

^{1}H NMR (500 MHz, DMSO) δ (ppm) 8.51 (t, J = 1.6 Hz, 3H, H_{2}), 8.44 (d, J = 9.3 Hz, 1H, H_{13}), 8.29 (d, J = 7.8 Hz, 1H, H_{21}), 8.28 (d, J = 7.6 Hz, 2H, $H_{17,19}$), 8.21 (d, J = 9.3 Hz, 1H, H_{14}), 8.16 (dd, J = 9.0 Hz, 2H, $H_{15,16}$), 8.08 (t, J = 7.6 Hz, 2H, H_{18}), 8.06 (d, J = 7.8 Hz, 2H, H_{20}), 7.75 (t, J = 1.6 Hz, 3H, H_{5}), 7.36 (d, J = 8.2 Hz, 2H, H_{8}), 7.32 (t, J = 1.6 Hz, 3H, H_{4}), 7.01 (d, J = 8.2 Hz, 2H, H_{9}), 4.77 (s, 2H, H_{11}), 3.79 (s, 9H, H_{6}); ^{13}C NMR (126 MHz, DMSO) δ (ppm) 141.94 (C_{2}), 139.37(C_{10}), 134.84 (C_{12}), 132.88 (C_{9}), 130.83 (C_{27}), 130.32 (C_{15}), 129.64 (C_{26}), 128.53 (C_{8}), 128.43 (C_{20}), 127.48 (C_{14}), 127.40 (C_{15}), 126.84 (C_{16}), 126.31 (C_{18}), 125.21 (C_{21}), 125.05 (C_{19}), 124.95 (C_{17}), 124.46 (C_{5}), 124.31 ($C_{22,23}$), 123.97 (C_{13}), 123.85 ($C_{24,25}$), 123.82 (C_{4}), 37.99 (C_{11}), 35.41 (C_{6}).

Absorption spectra in acetonitrile solution (λ/nm , $\varepsilon/M^{-1}cm^{-1}$): 338 (40400), 323 (28400), 309 (12000), 297 (4600), 272 (38900), 261 (23000), 238 (63400), 230 (43100).

HR-MS (ToF-SIMS, positive mode): m/z [**B** · $PF_6]^+$ = 693.2422 (Expected: 693.2501).

HR-MS (ToF-SIMS, negative mode): m/z [**B** · $3PF_6]^-$ = 983.1774 (Expected: 983.1785).

- Bis{4-(pyrenylmethyl)phenyl[tris(3-methylimidazol-1-ylidene)]borate} Fe(III) hexafluorophosphate, **[Fe(pyphtmeimb)$_2$](PF$_6$), 3**

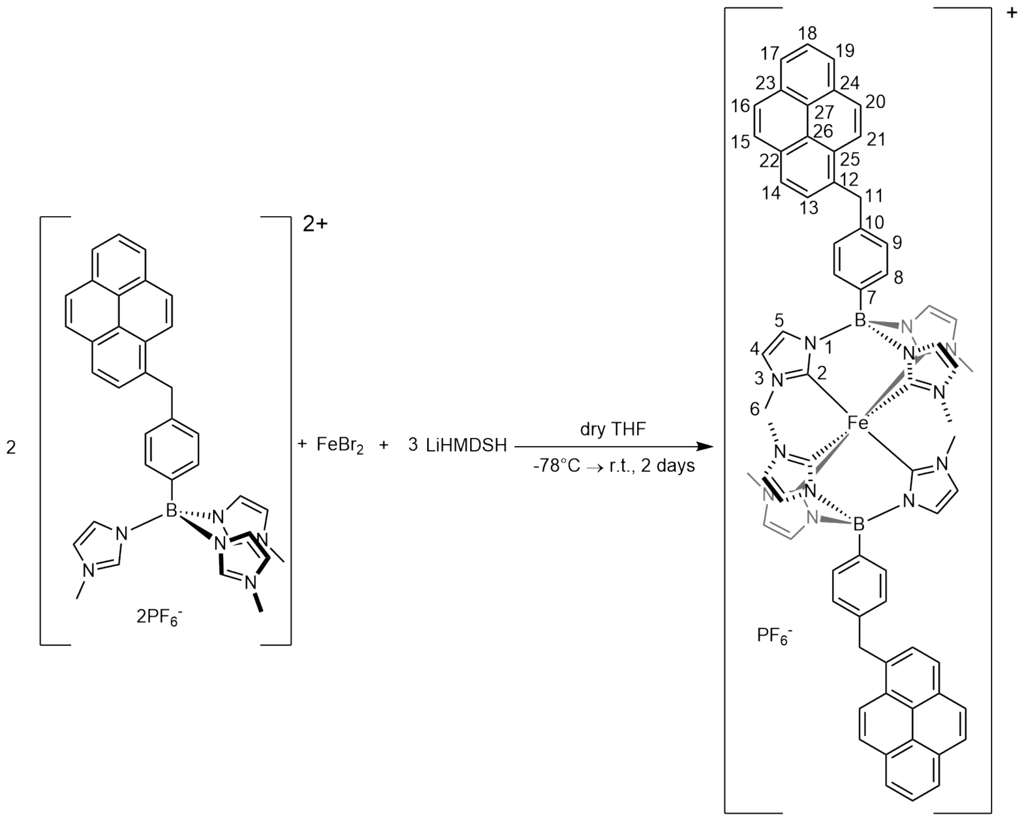

Supplementary Fig. 9. Synthetic scheme of **3**.

The synthetic procedure was the same as that of [Fe(pytmeimb)$_2$](PF$_6$) using 250 mg of **pyphtmeimb** (0.3 mmol), 7 mL of THF, 1.4 mL of LiHMDS and a solution of iron(II) bromide (32.4 mg, 0.15 mmol in 3.5 mL of dry THF). After 48 h of stirring, the reaction mixture was concentrated under reduced pressure, solubilized in dichloromethane and filtered. The solid was solubilized in ethanol and after the addition of an excess of NH_4PF_6, the formation of a red solid occurred. The solid was dissolved in the minimum amount of dichloromethane and eluted through a size-exclusion column using Sephadex G10 as stationary phase. Then, it was purified through PLC (1.2 mm thickness) on alumina using toluene/acetonitrile (3:1 V/V) as eluent and dichloromethane. The product of interest was recovered with dichloromethane extractions, concentrated and reprecipitated in diethyl ether, with a yield of 15 % (29 mg).

^{1}H NMR (500 MHz, CD_3CN) δ (ppm) 14.55 (d, J = 5.5 Hz, 4H, H_8), 10.27 (d, J = 5.5 Hz, 4H, H_9), 9.89 (d, J = 9.5 Hz, 2H, H_{13}), 9.42 (d, J = 8 Hz, 2H, H_{21}), 9.00 (d, J = 8.0 Hz, 2H, H_{20}), 8.93 (d, J = 9.5 Hz, 2H, H_{14}), 8.61 (dd, 7.9 Hz, 1.1 Hz, 2H, H_{19}), 8.58 (d, 9.1 Hz, 2H, H_{15}), 8.51 (dd, J = 7.9 Hz, 1.1 Hz, 2H, H_{17}), 8.43 (d, J = 9.1 Hz, 2H, H_{16}), 8.28 (t, J = 7.9 Hz, 2H, H_{18}), 6.49 (s, 4H, H_{11}), 4.88 (s, 18H, H_6), 1.57 (s, 6H, H_5), -12.37 (s, 6H, H_4); ^{13}C NMR (126 MHz, CD_3CN) δ (ppm) 145.21 (C_{10}), 140.68 (C_8), 137.77 (C_{12}), 132.95 (C_9), 130.70 (C_{21}), 129.26 (C_{14}), 129.01 (C_{15}), 128.29 (C_{16}), 127.55 (C_{18}), 126.97 (C_{20}), 126.49 (C_{19}), 126.42 (C_{17}), 126.16 (C_{13}), 49.59 (C_4), 41.54 (C_{11}), 13.28 (C_5), -28.61 (C_6).

Absorption spectra in acetonitrile solution (λ/nm , ε/$M^{-1}cm^{-1}$): 501 (3200), 338 (63100), 322 (45800), 309 (22900), 295 (12500), 271 (65700), 261 (45600).

Elemental analysis calculated (%) for $C_{70}H_{60}B_2F_6FeN_{12}P$: C 65.09, H 4.68, N 13.01, found (%): C 65.03, H 4.64, N 13.72.

HR-MS (ToF-SIMS, positive mode): m/z [**3**]$^+$ = 1146.4485 (Expected: 1146.4599).

HR-MS (ToF-SIMS, negative mode): m/z [**3** · 2PF_6]$^-$ = 1436.3949 (Expected: 1436.3882).

NMR Spectra

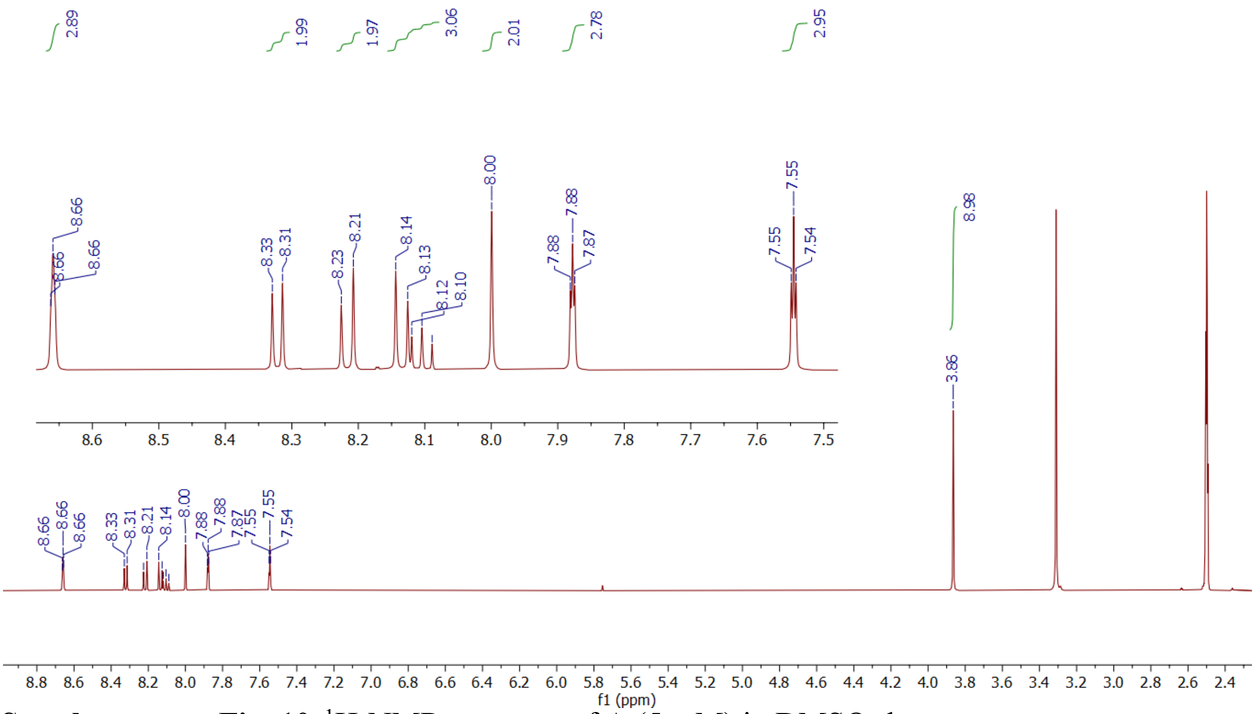

Supplementary Fig. 10. ^{1}H-NMR spectrum of **A** (5 mM) in DMSO-d_6.

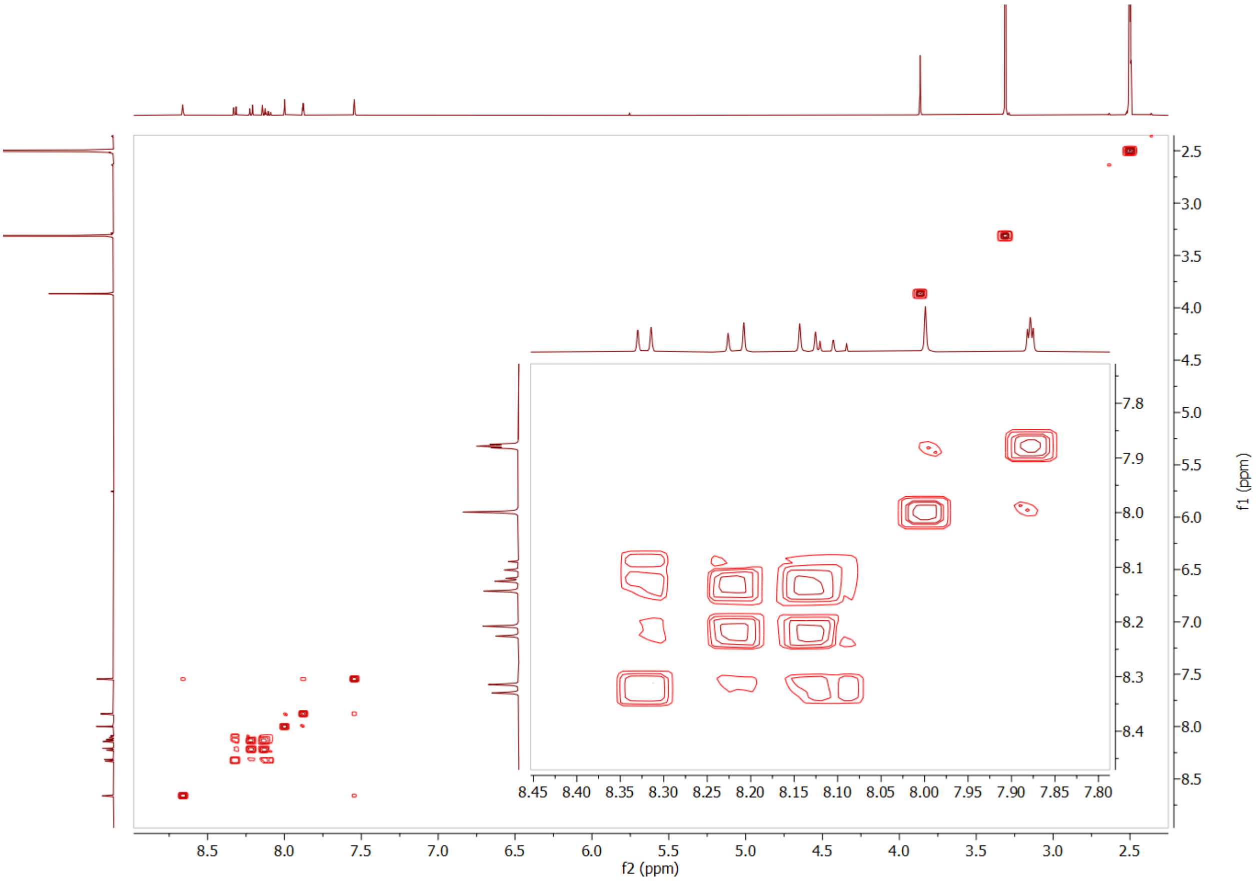

Supplementary Fig. 11. gCOSY spectrum of **A** (5 mM) in DMSO-d_6.

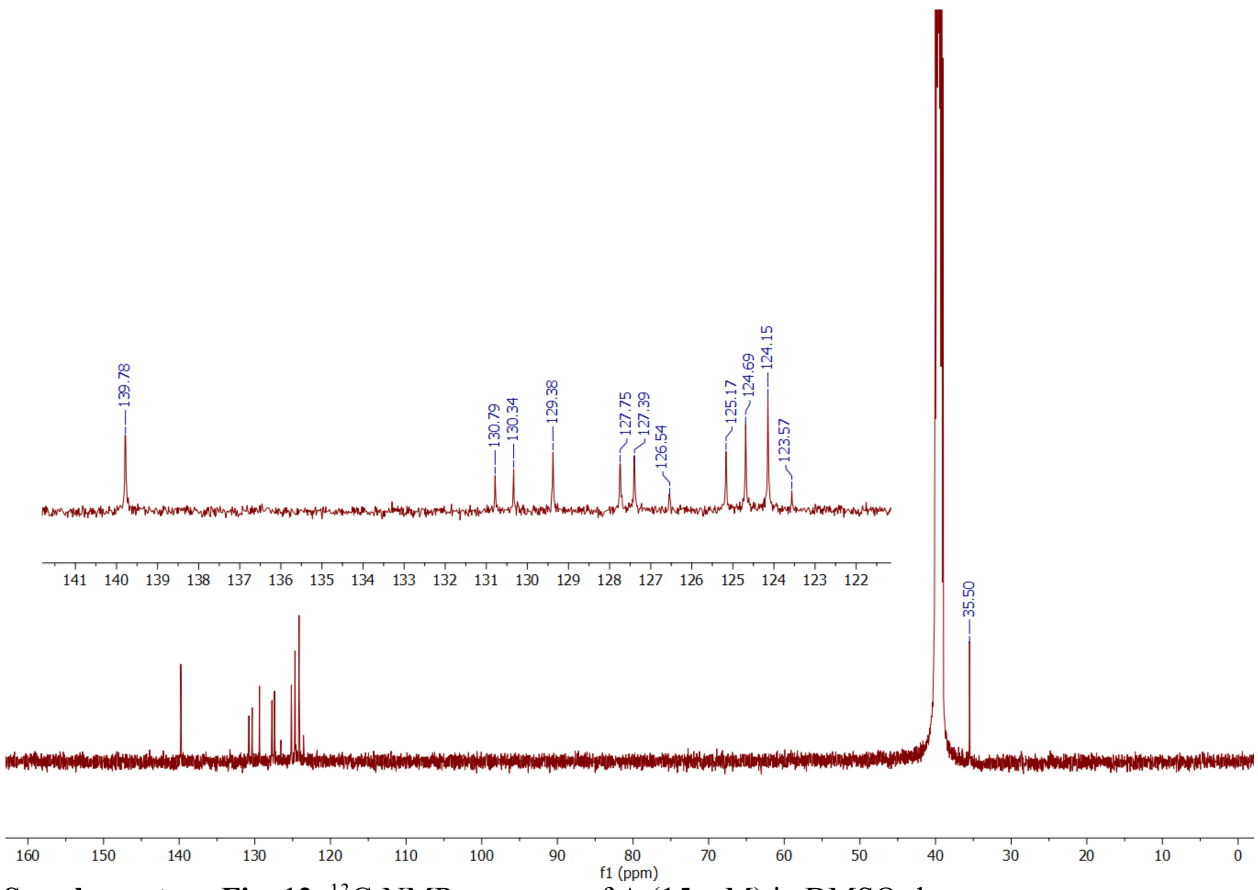

Supplementary Fig. 12. ^{13}C-NMR spectrum of **A** (15 mM) in DMSO-d_6.

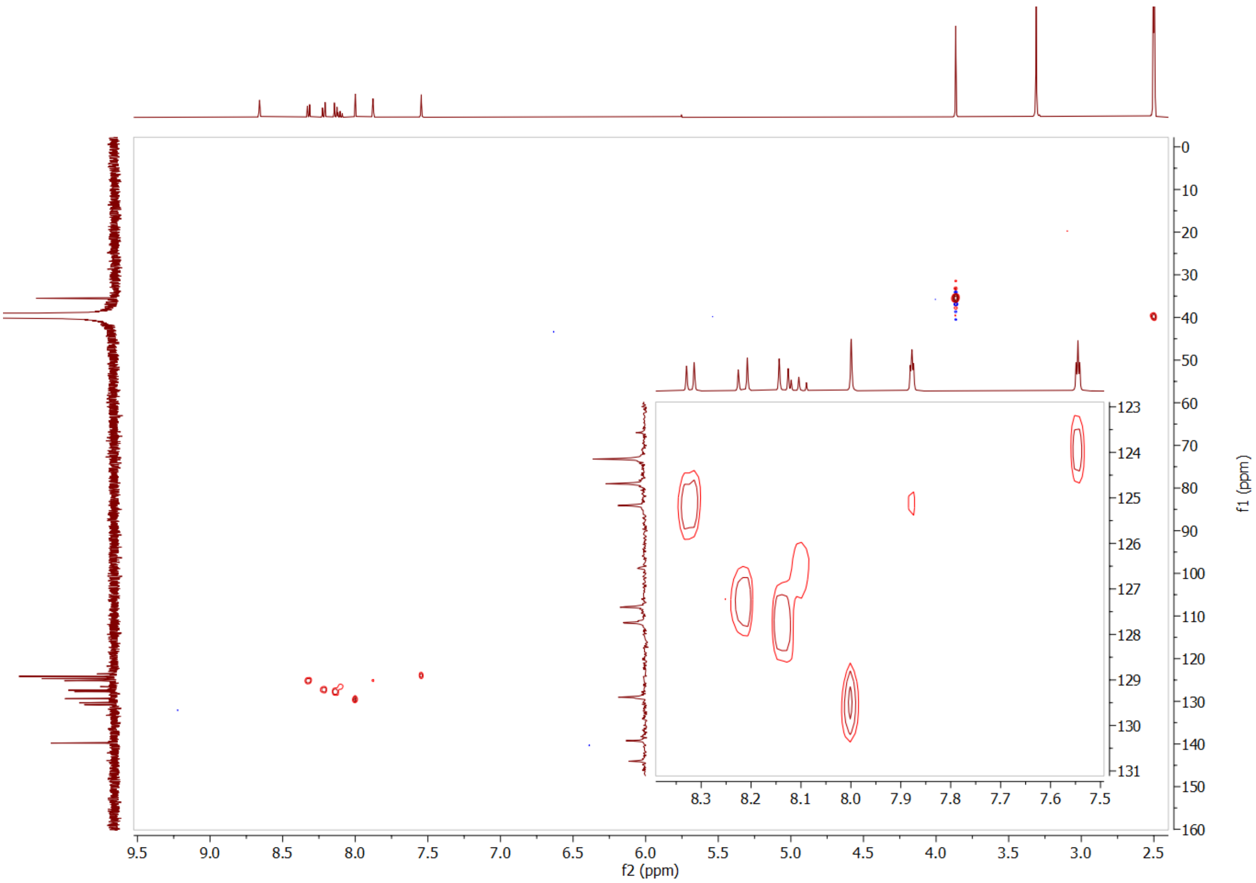

Supplementary Fig. 13. gHSQC spectrum of **A** (15 mM) in DMSO-d6.

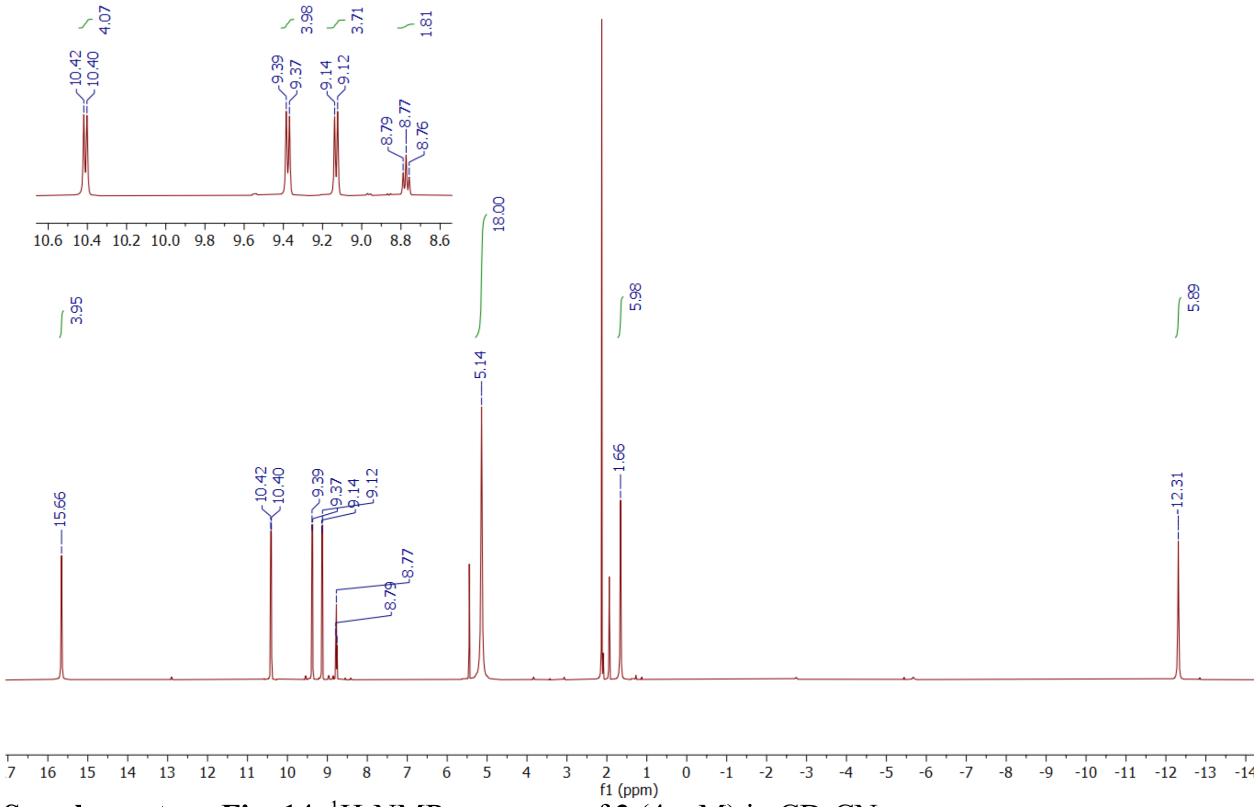

Supplementary Fig. 14. ^{1}H-NMR spectrum of **2** (4 mM) in CD_3CN.

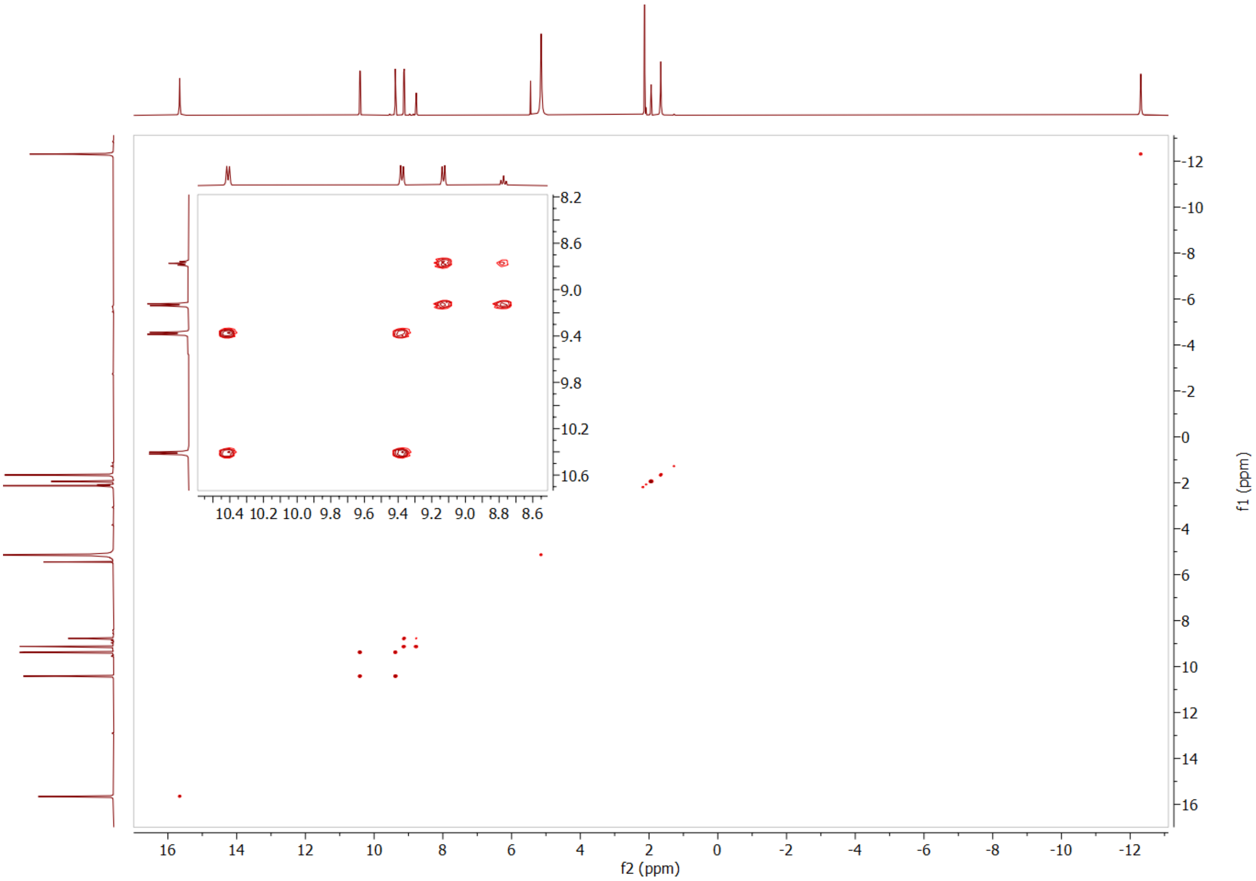

Supplementary Fig. 15. gCOSY spectrum of **2** (5 mM) in CD_3CN.

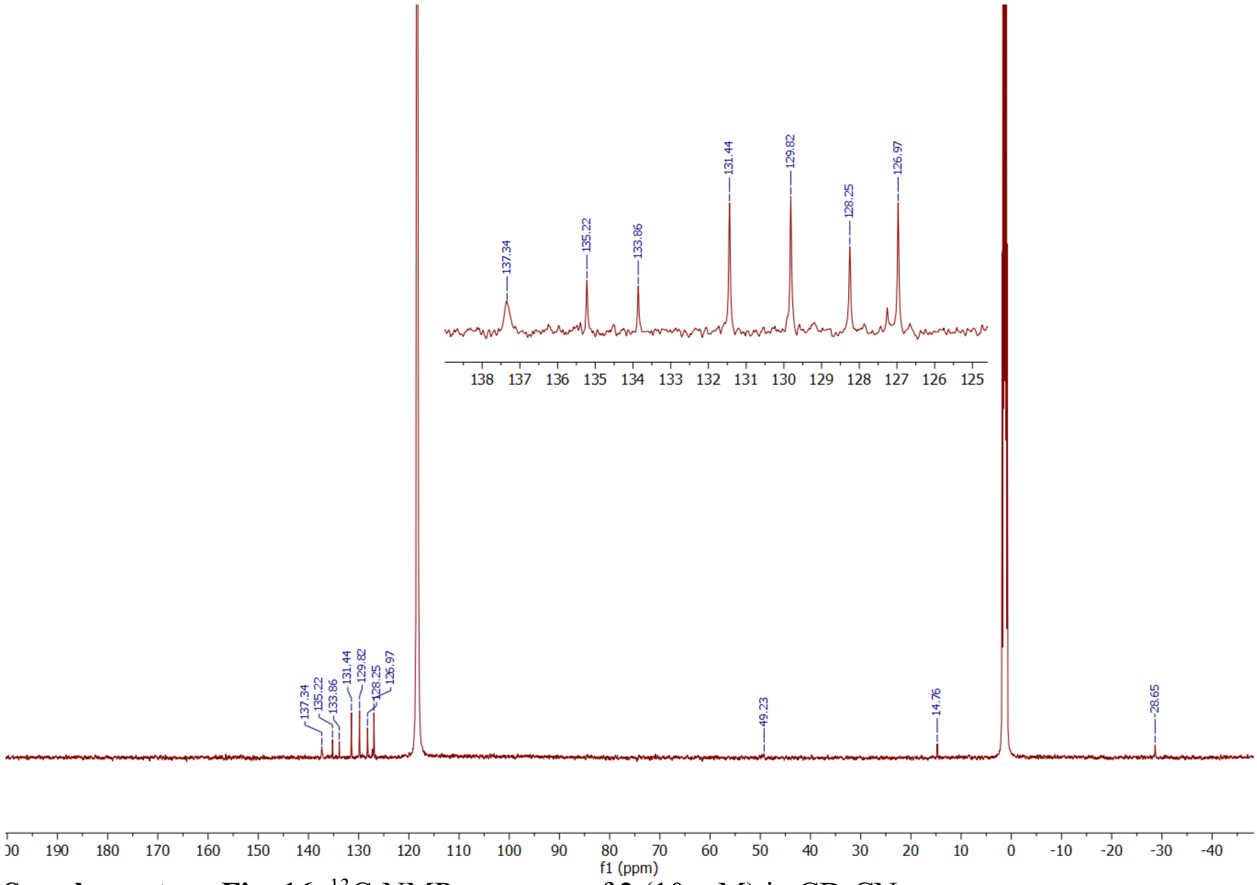

Supplementary Fig. 16. ^{13}C-NMR spectrum of **2** (10 mM) in CD_3CN.

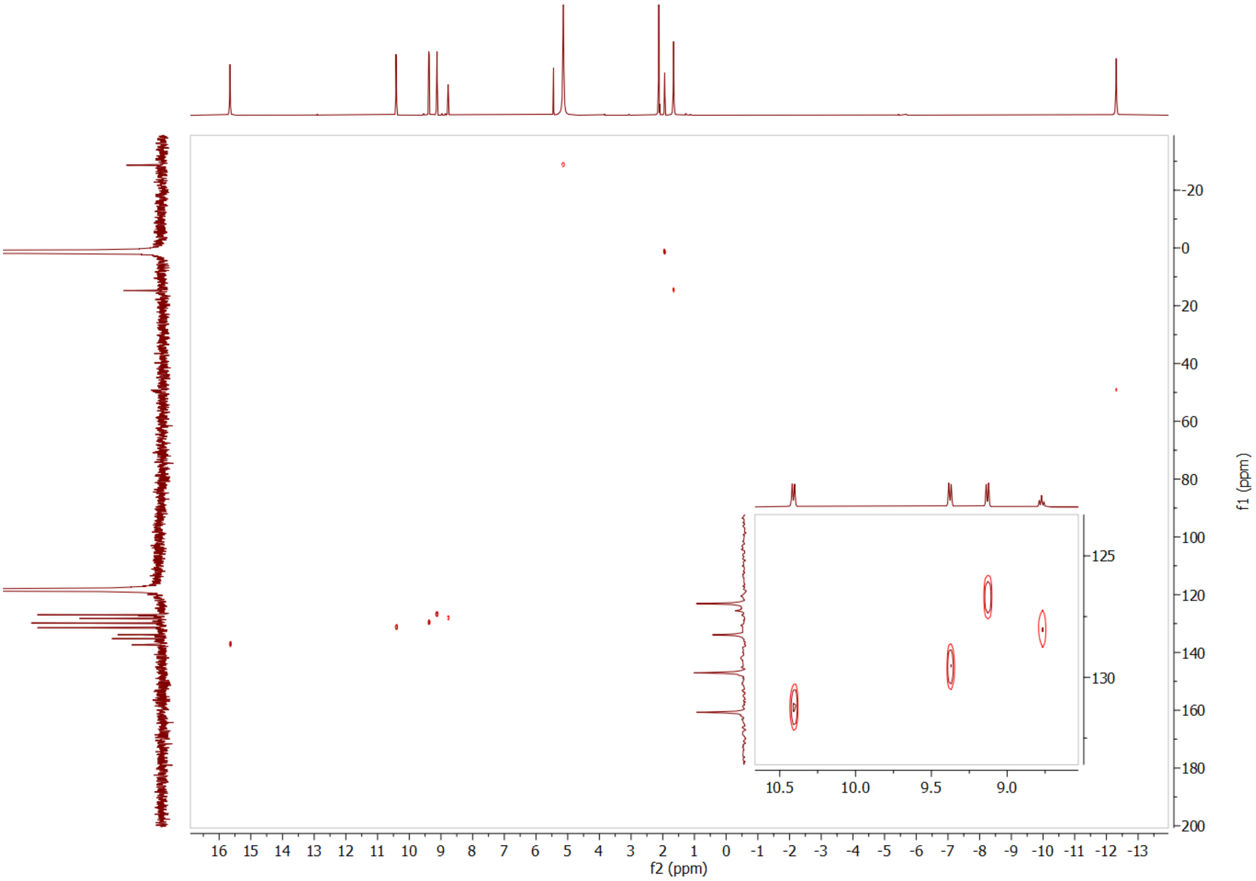

Supplementary Fig. 17. HMQC spectrum of **2** (10 mM) in CD_3CN.

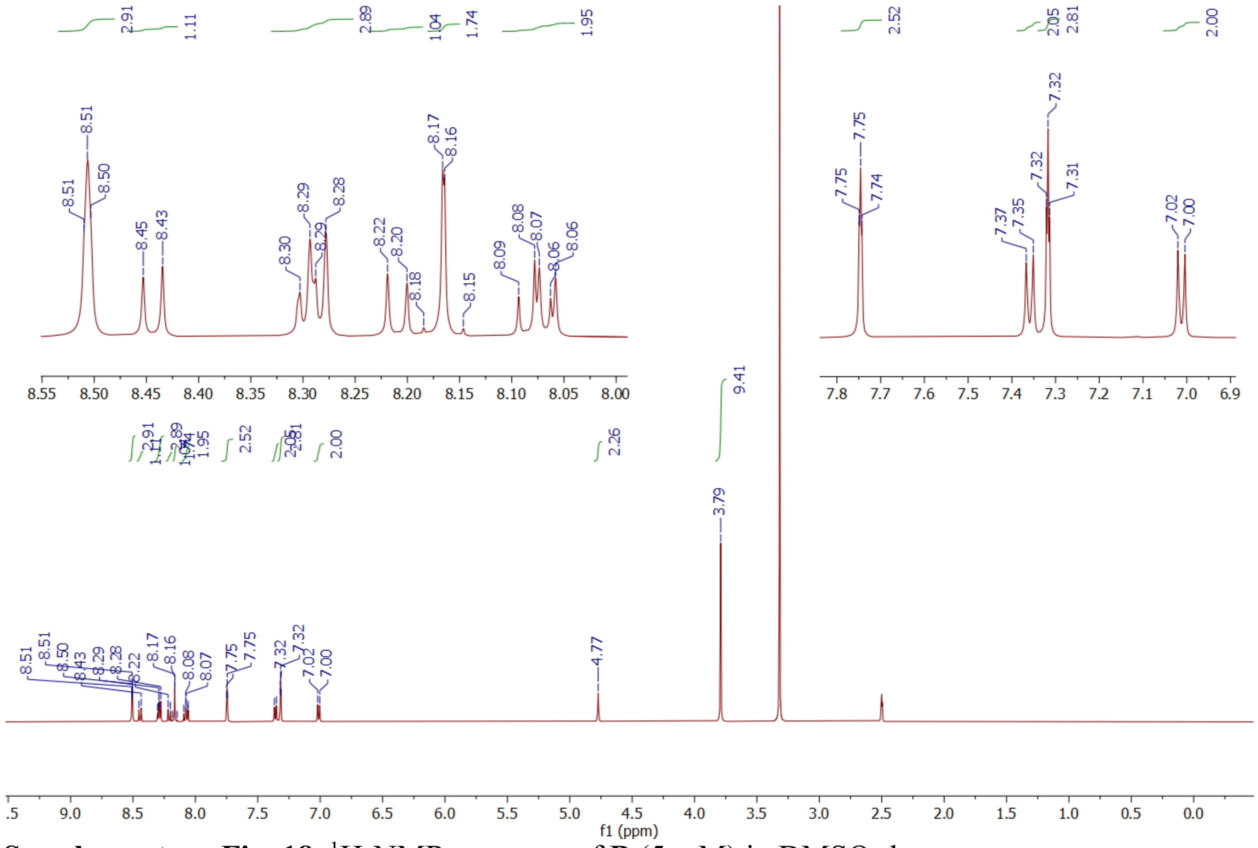

Supplementary Fig. 18. ^{1}H-NMR spectrum of **B** (5 mM) in DMSO-d$_6$.

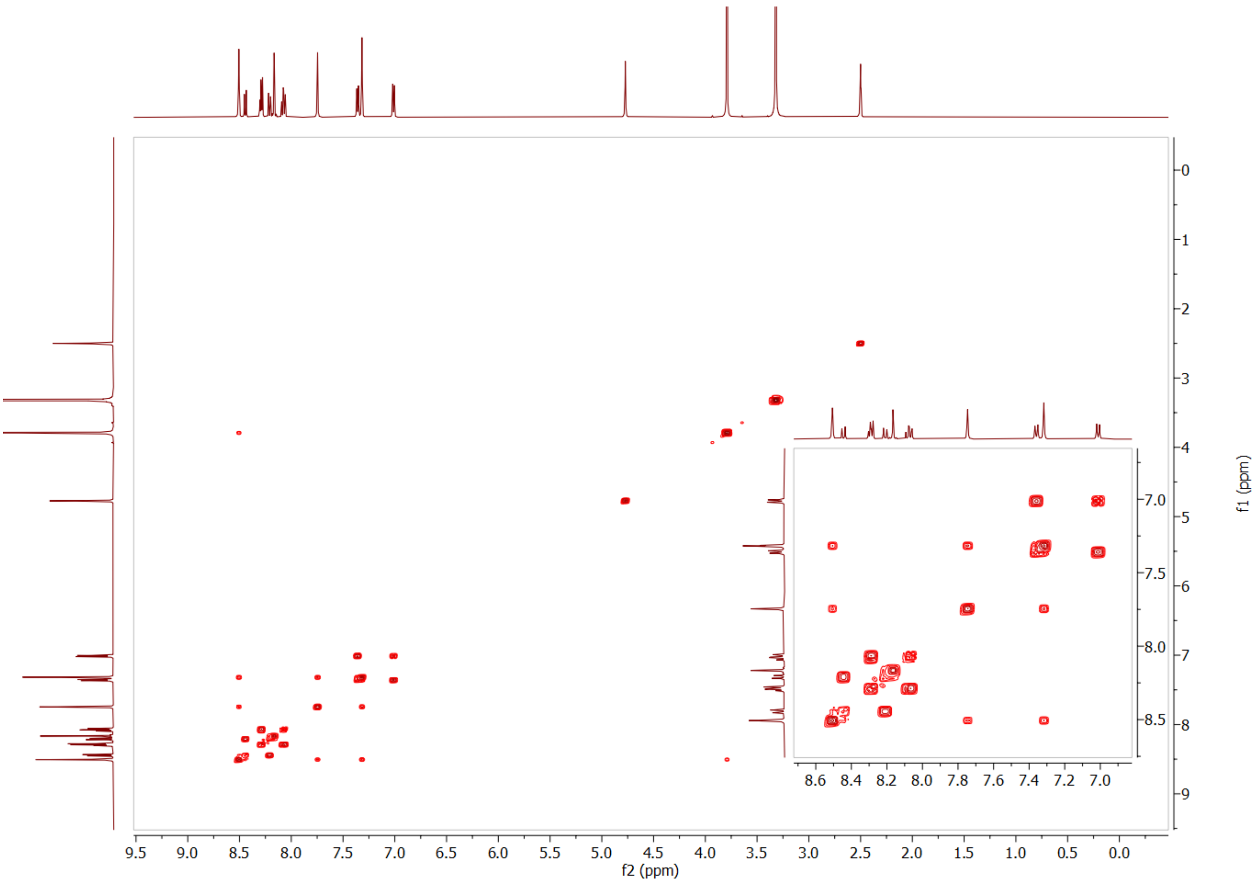

Supplementary Fig. 19. gCOSY spectrum of **B** (5 mM) in DMSO-d6.

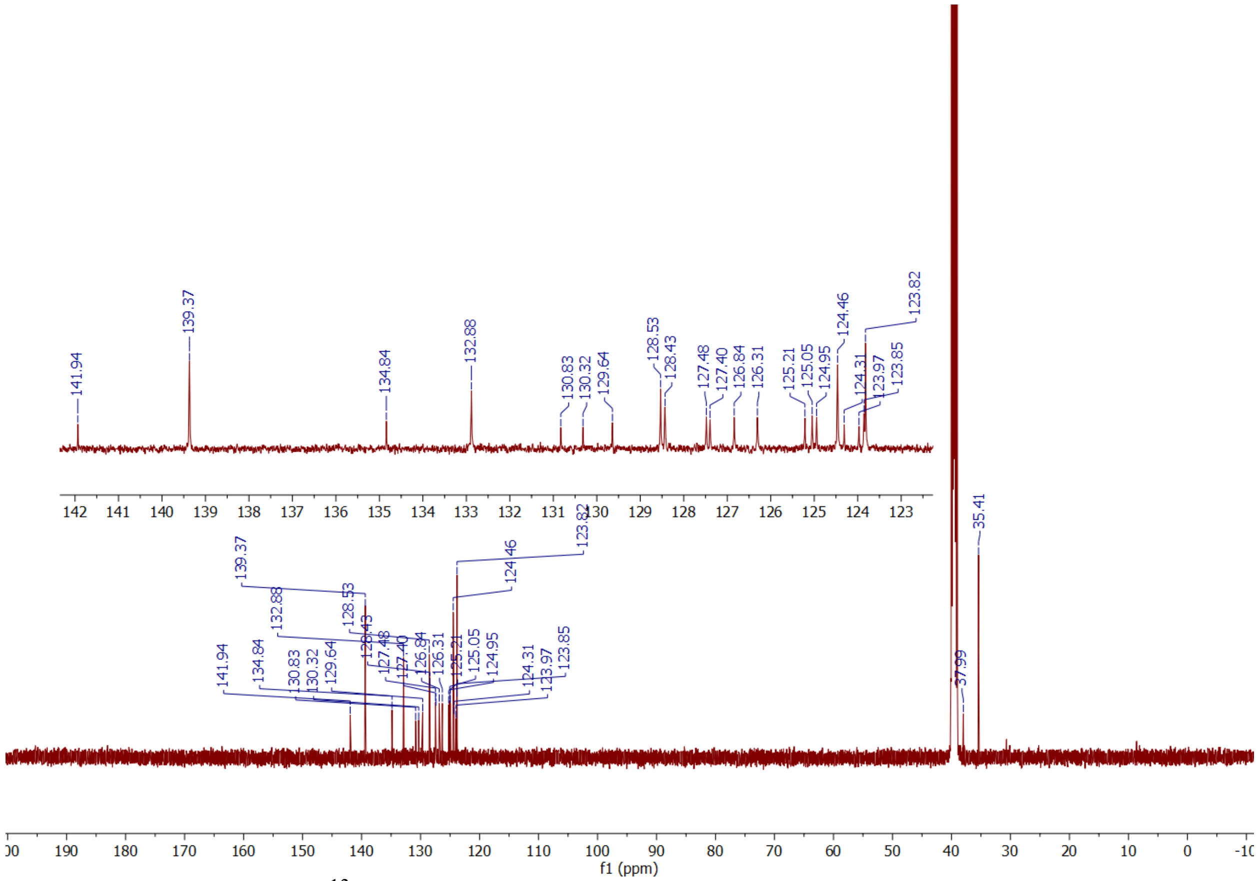

Supplementary Fig. 20. ^{13}C-NMR spectrum of **B** (15 mM) in DMSO-d_6.

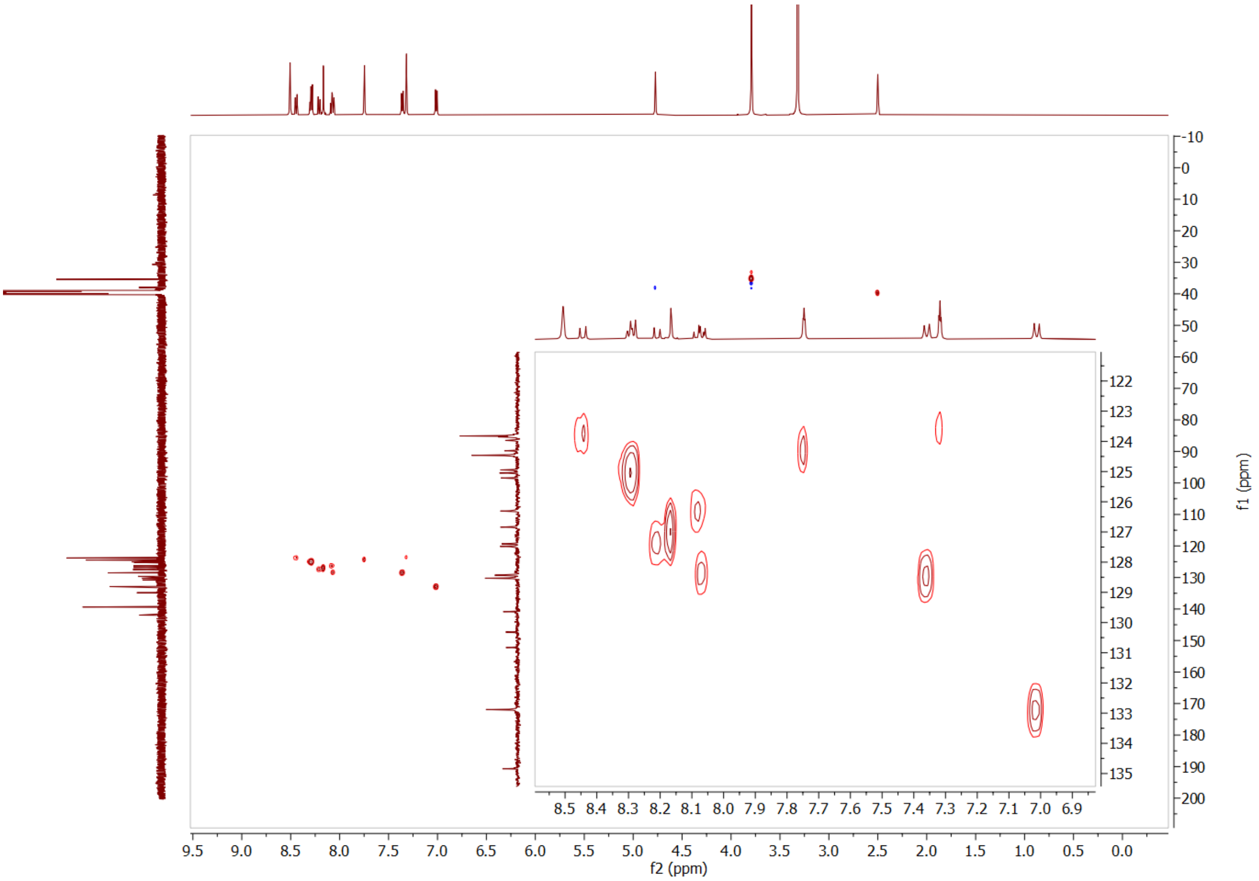

Supplementary Fig. 21. gHSQC spectrum of **B** (15 mM) in DMSO-d$_6$.

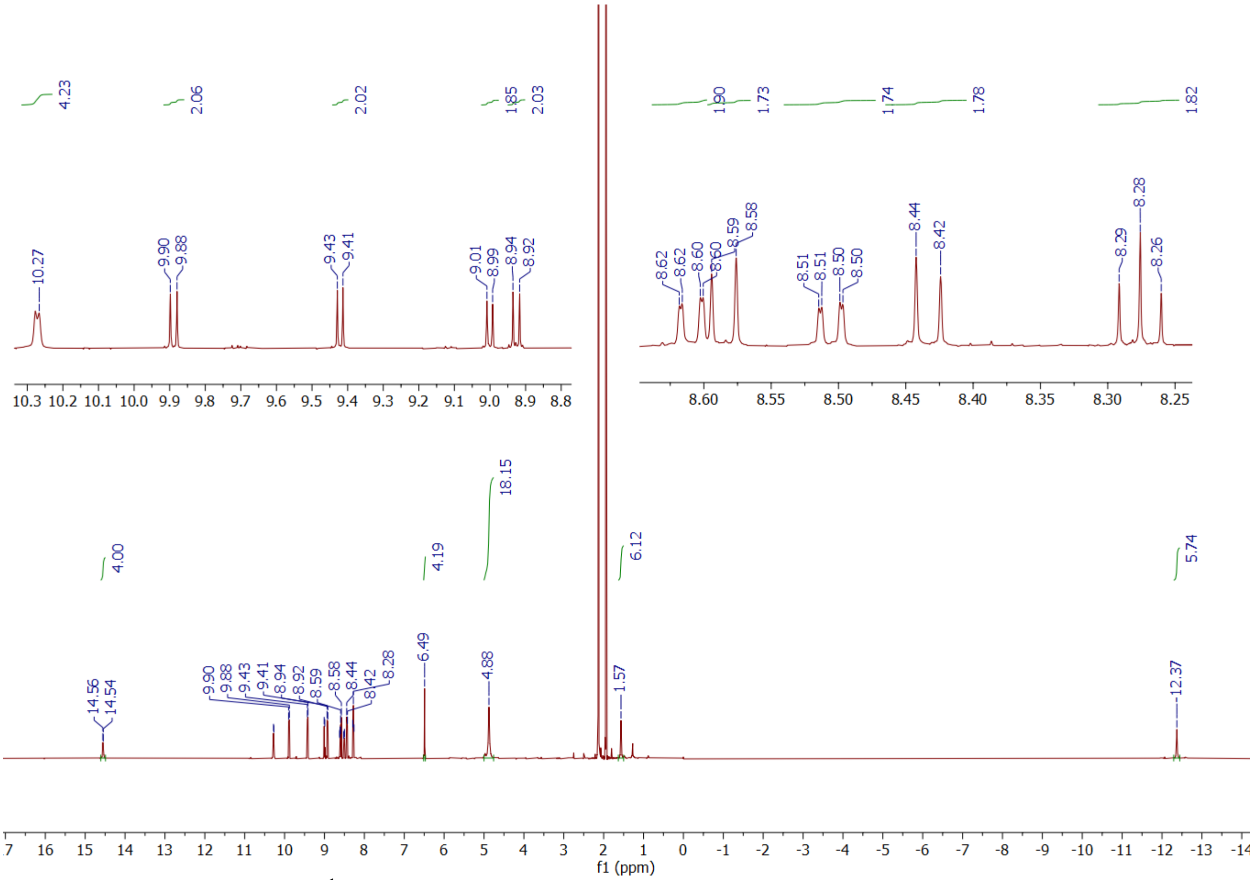

Supplementary Fig. 22. ^{1}H-NMR spectrum of **3** (4 mM) in CD_3CN.

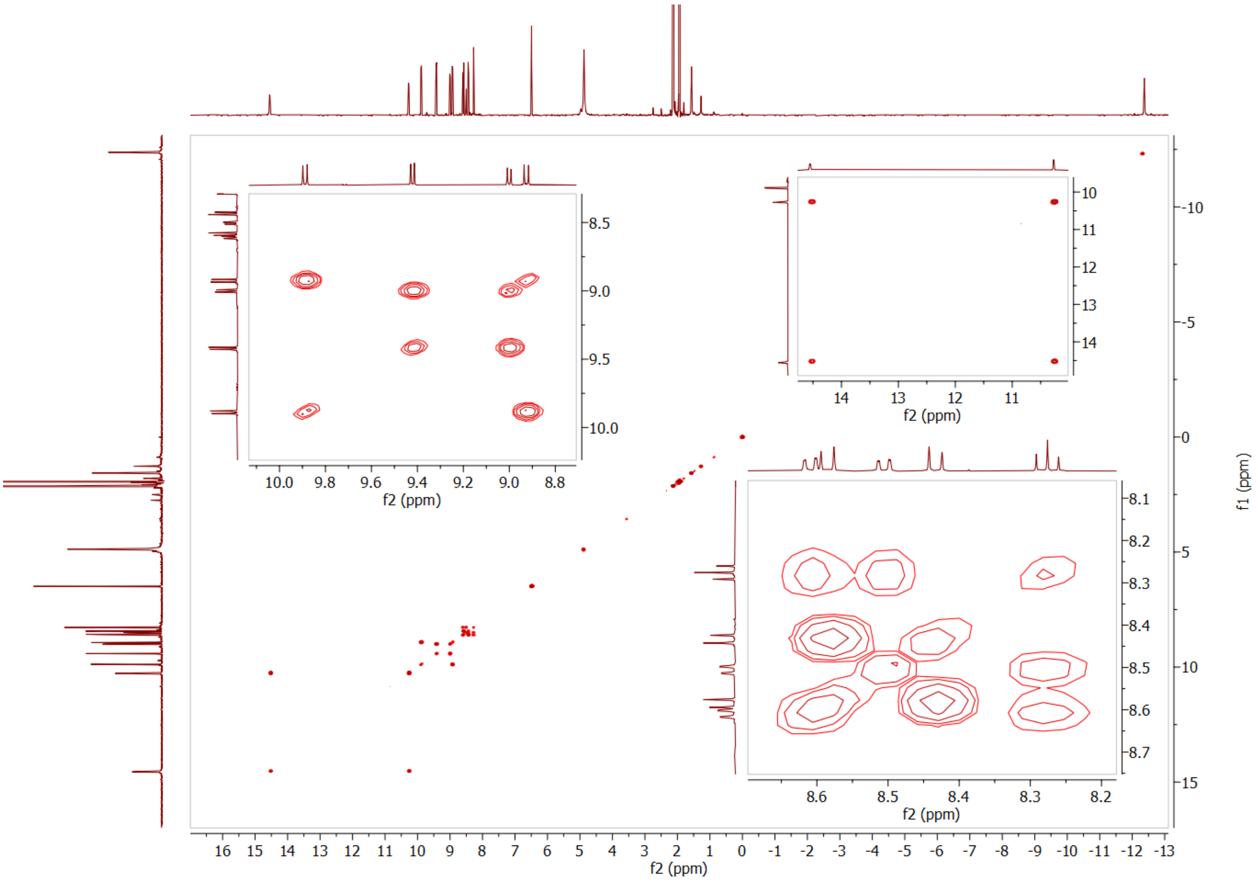

Supplementary Fig. 23. gCOSY spectrum of **3** (5 mM) in CD_3CN.

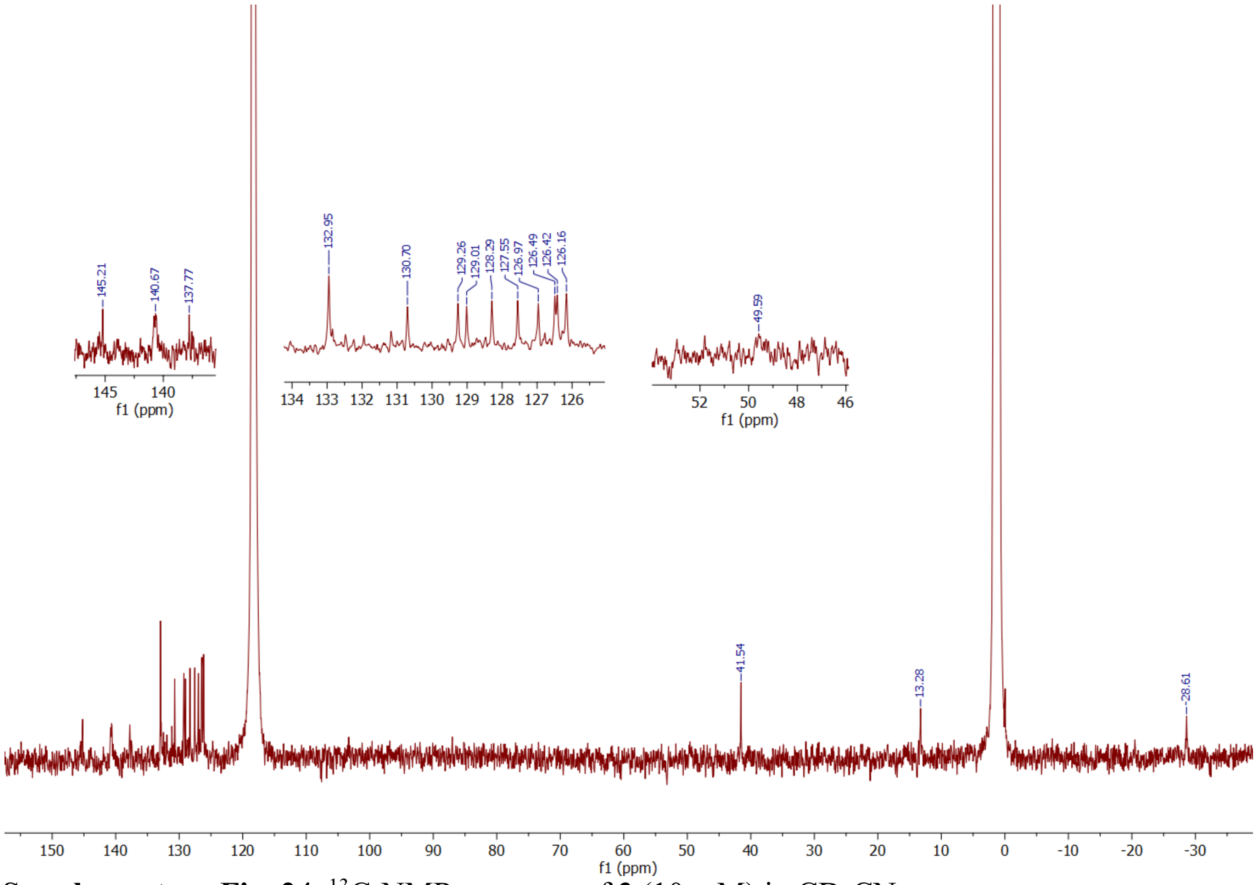

Supplementary Fig. 24. ^{13}C-NMR spectrum of **3** (10 mM) in CD_3CN.

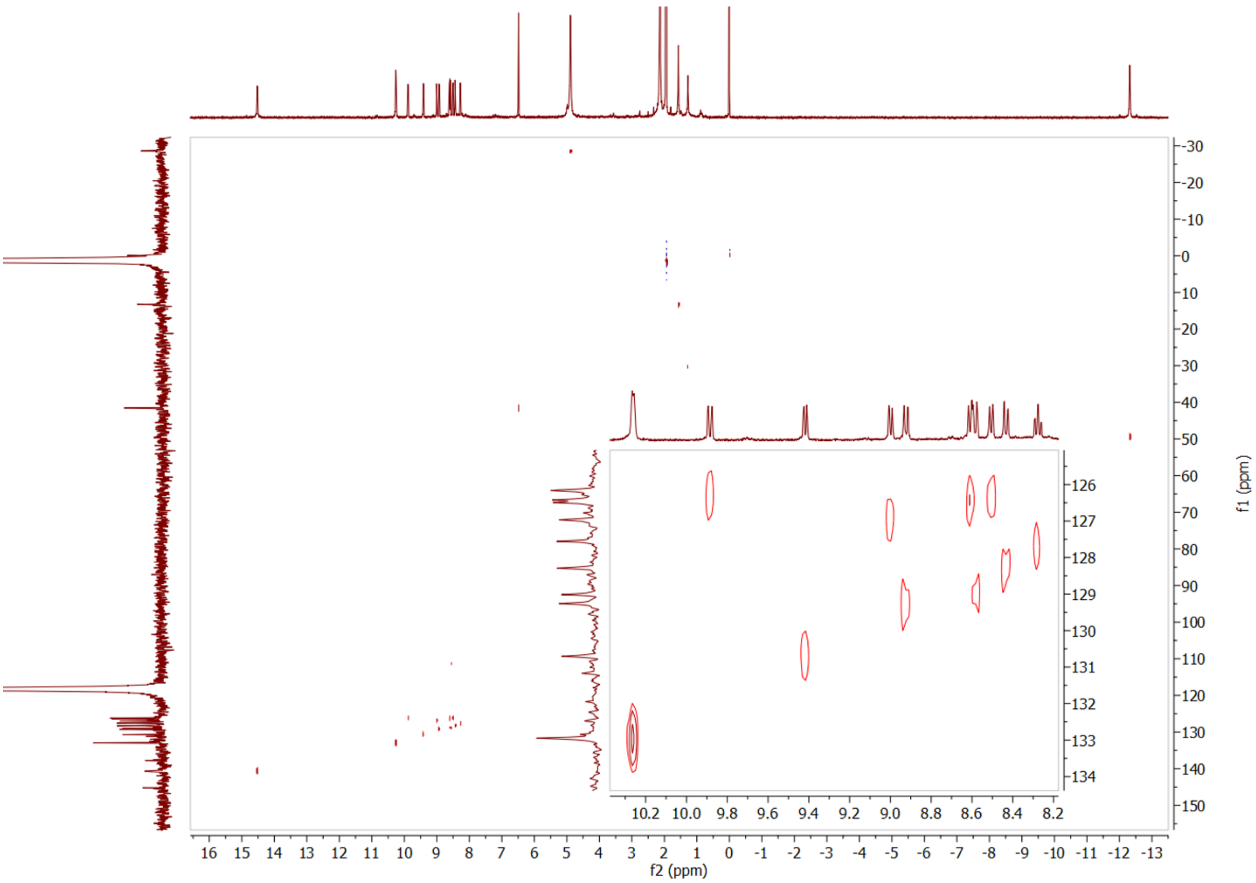

Supplementary Fig. 25. HMQC spectrum of **3** (10 mM) in CD_3CN.

High-resolution Mass Spectra

Sample preparation

A small aliquot of each compound was dissolved in acetonitrile (> 99.9%, Aldrich) to obtain a very dilute solution, which concentration is in the micromolar range.

A few microliters of each solution were drop cast either on a oxygen plasma cleaned flat silicon substrate (March Instrument Plasmod – 75W, 0.4 torr, 60 s) and freshly etched Ag substrate (HNO_3 10%, 50°C) and the solvent was allowed to evaporate.

ToF-SIMS measurements

Time-of-Flight Secondary Ion Mass Spectrometry (ToF-SIMS) analysis was performed by a IONTOF ToF-SIMS IV instrument (IONTOF GmbH) using a Bi^+ analysis beam (25 keV, 0.7 pA), rastered over an area of 300 x 300 µm². The primary ion fluence was maintained below $1x10^{12}$ ions/cm², in order to ensure static conditions. The spectra were recorded in both positive and negative polarity. Mass resolution (m/Δm) @m/z = 28 (Si^+ ion) was 8500. The spectra were internally mass calibrated using CH_3^+, $C_2H_3^+$, $C_4H_7^+$ ions for the positive polarity and C^-, C_2^- and C_4^- ions for negative polarity. The peak assignment was conducted on the basis of the exact mass and of the isotopic distribution. The choice of two different substrates is motivated by the need to exclude possible mass interferences of substrate signals (e.g. silver clusters) and/or to check the isotope distributions, which can be modified to a different degree of protonation/cationization on the two substrates.

**A**	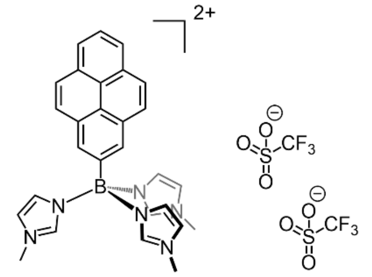<br>Chemical Formula: $C_{30}H_{27}BF_6N_6O_6S_2$<br>Molecular Weight: 756.50
**B**	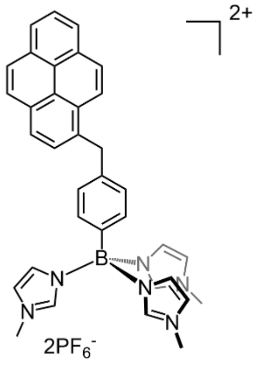<br>Chemical Formula: $C_{35}H_{33}BF_{12}N_6P_2$<br>Molecular Weight: 838.42
**2**	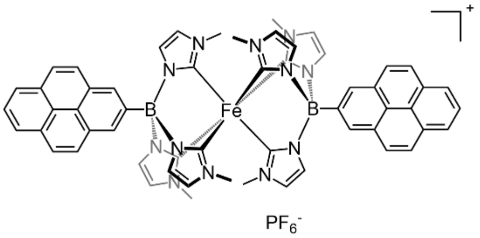<br>Chemical Formula: $C_{56}H_{48}B_2F_6FeN_{12}P$<br>Molecular Weight: 1111.49

**3**	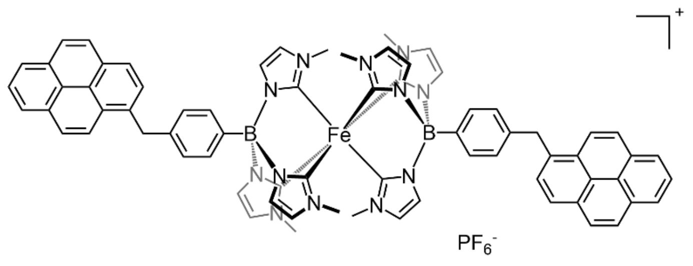<br>Chemical Formula: $C_{70}H_{60}B_2F_6FeN_{12}P$<br>Molecular Weight: 1291.74

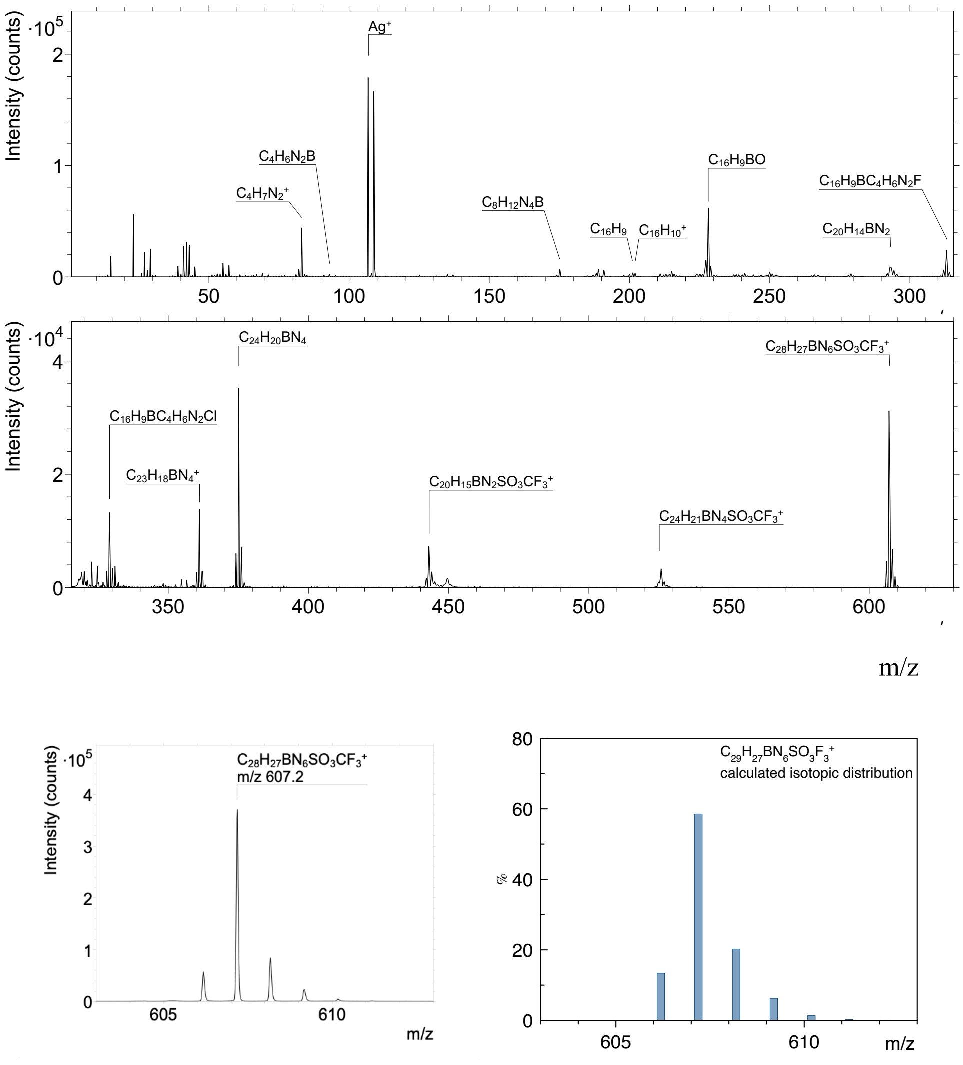

Ion	Mass	Description (ion charge omitted)
$C_4H_7N_2^+$	83.0691	methylimidazolium
$C_4H_6N_2B^+$	93.0631	
$C_8H_{12}N_4B^+$	175.1145	
$C_{16}H_{10}^+$	202.0645	pyrene
$C_{16}H_9BO^+$	228.0759	
$C_{20}H_{14}BN_2^+$	293.1017	$[A – (C_4H_7N_2)_2]$
$C_{16}H_9BC_4H_6N_2F^+$	313.1183	$[A – (C_4H_7N_2)_2] \cdot F$
$C_{16}H_9BC_4H_6N_2Cl^+$	329.0931	$[A – (C_4H_7N_2)_2] \cdot Cl$
$C_{23}H_{18}BN_4^+$	361.1505	$[A – (C_4H_7N_2) – CH_2]$
$C_{24}H_{20}BN_4^+$	375.1730	$[A – (C_4H_7N_2)]$
$C_{21}H_{15}BN_2SO_3F_3^+$	443.0565	$[A – (C_4H_7N_2)_2] \cdot CF_3SO_3$
$C_{25}H_{21}BN_4SO_3F_3^+$	525.1241	$[A – (C_4H_7N_2)] \cdot CF_3SO_3$
$C_{29}H_{27}BN_6SO_3F_3^+$	607.1849	$[A] \cdot CF_3SO_3$

Supplementary Fig. 26. Mass spectra of **A** and table of assignment. Positive polarity, substrate: Ag.

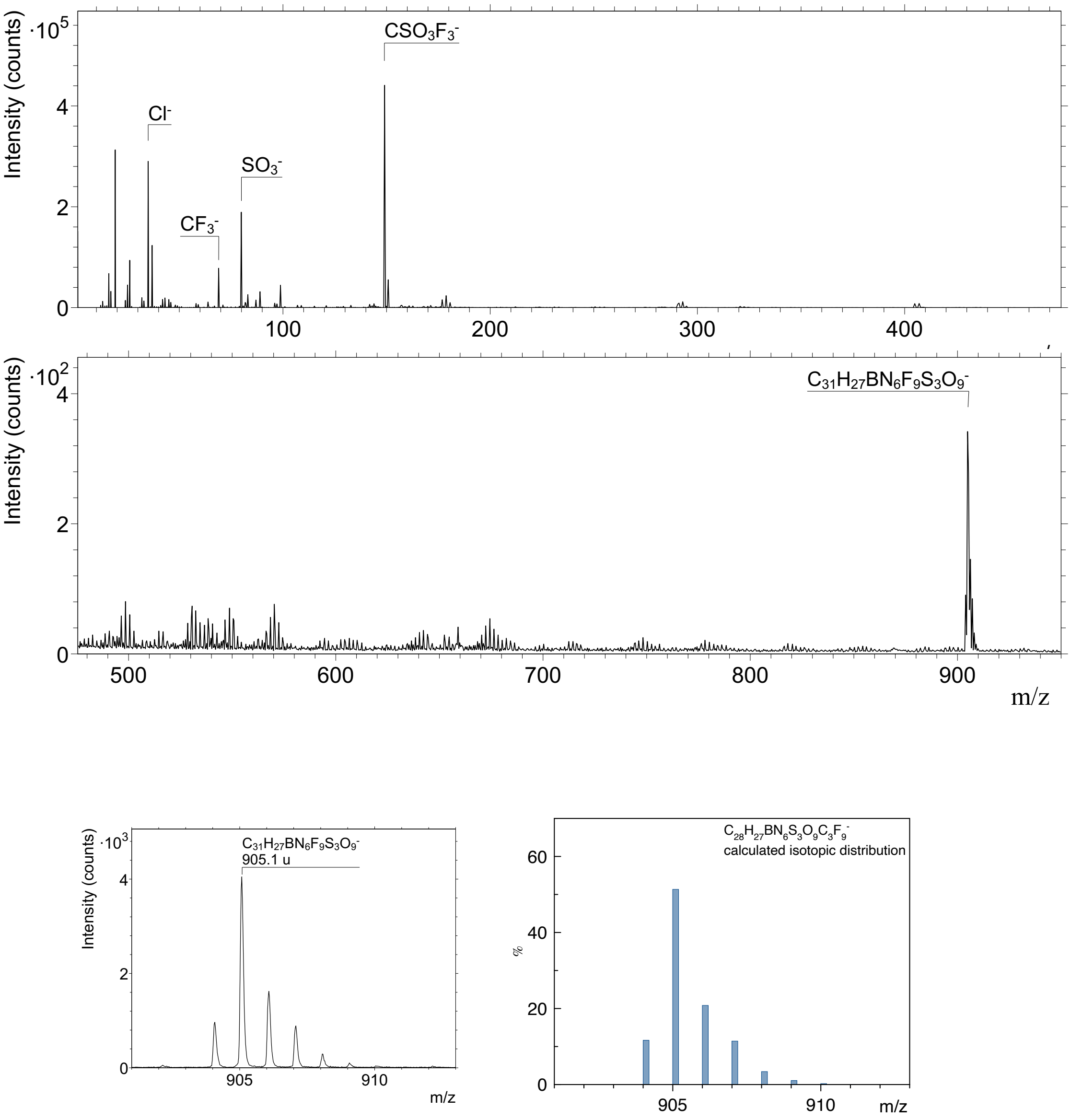

Ion	Mass (u)	Description (ion charges omitted)
$CSO_3F_3^-$	148.9538	counter-ion
$C_{31}H_{27}BN_6S_3O_9F_9^-$	905.0895	[A] · $3CF_3SO_3$

Supplementary Fig. 27. Mass spectra of **A** and table of assignment. Negative polarity, substrate: Ag.

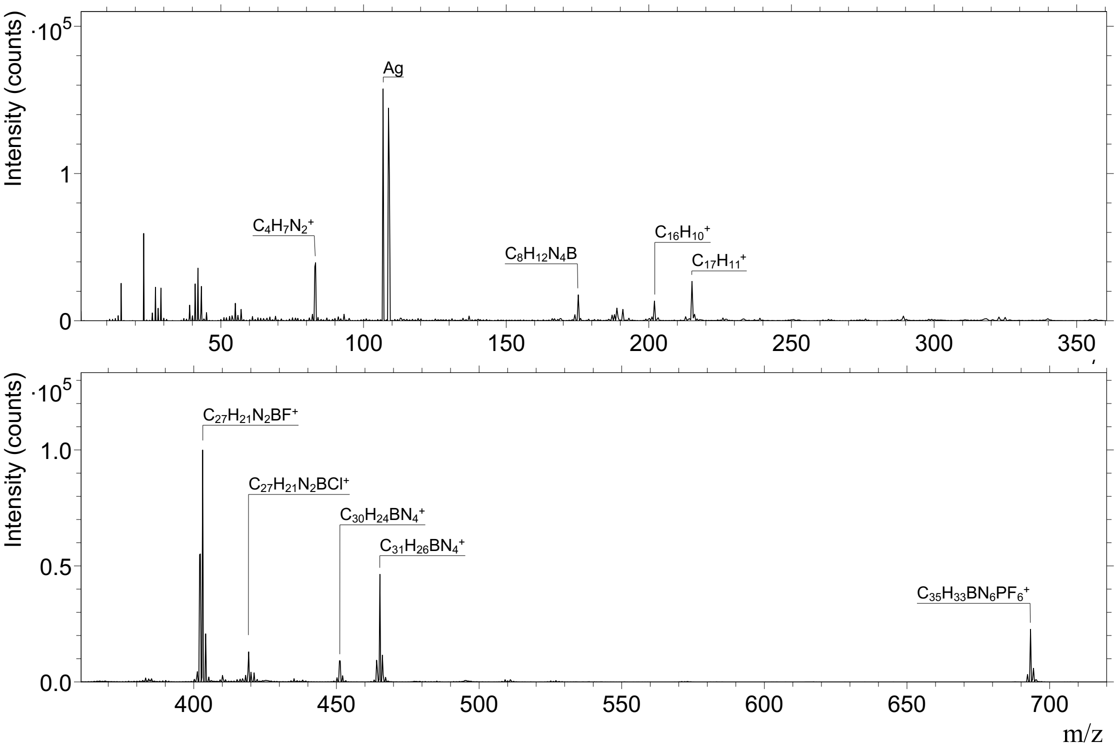

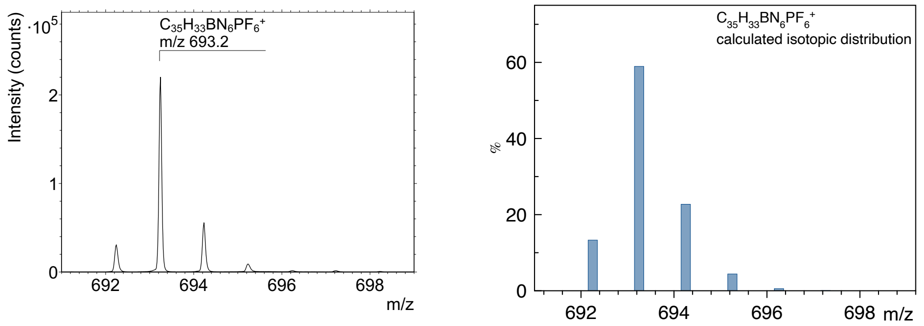

Ion	Mass (u)	Description (ion charges omitted)
$C_4H_7N_2^+$	83.0692	methylimidazolium
$C_8H_{12}N_4B^+$	175.1214	
$C_{16}H_{10}^+$	202.0928	pyrene
$C_{17}H_{11}^+$	215.0854	pyrene + CH
$C_{27}H_{21}N_2BF^+$	403.1715	$[(B) - (C_4H_7N_2)_2]\cdot F$
$C_{27}H_{21}N_2BCl^+$	419.141	$[(B) - (C_4H_7N_2)_2]\cdot Cl$
$C_{31}H_{26}BN_4^+$	465.2196	$[(B) - (C_4H_7N_2)]$
$C_{35}H_{33}BN_6PF_6^+$	693.2422	$[(B)]\cdot PF_6$

Supplementary Fig. 28. Mass spectra of **B** and table of assignment. Positive polarity, substrate: Ag.

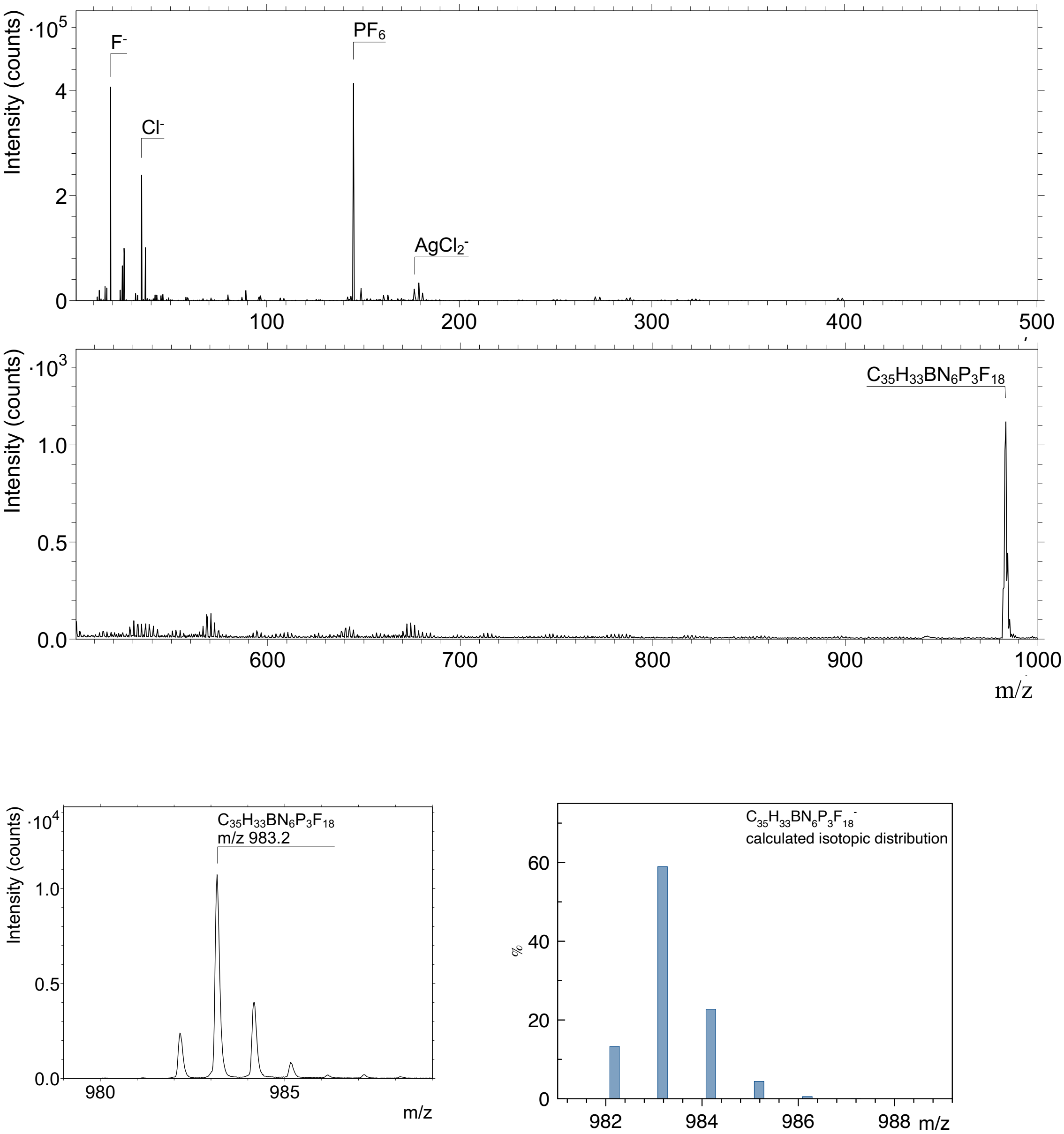

	Mass (u)	Description (ion charges omitted)
PF_6^-	144.9635	counter-ion
$C_{35}H_{33}BN_6P_3F_{18}^-$	983.1774	[L3] · $3PF_6$

Supplementary Fig. 29. Mass spectra of **B** and table of assignment. Negative polarity, substrate: Ag.

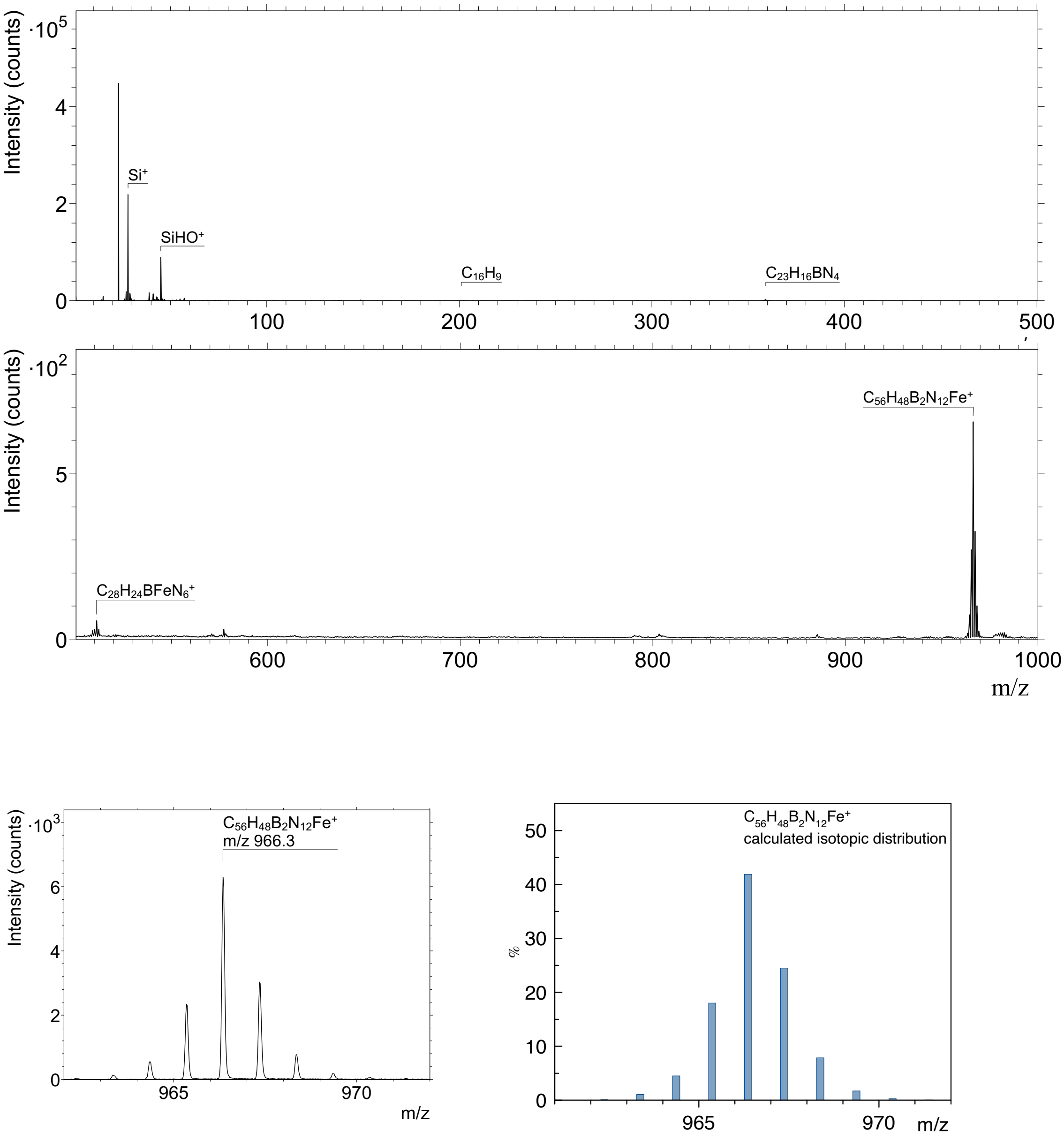

Ion	Mass (u)	Description[a]
$C_{16}H_9^+$	201.0649	pyrene
$C_{28}H_{24}BN_6Fe^+$	511.1490	FeA
$C_{56}H_{48}B_2N_{12}Fe^+$	966.3546	$Fe(A)_2$

(a) ion charges are omitted in the description

Supplementary Fig. 30. Mass spectra of complex **2** and table of assignment. Positive polarity, substrate: Si).

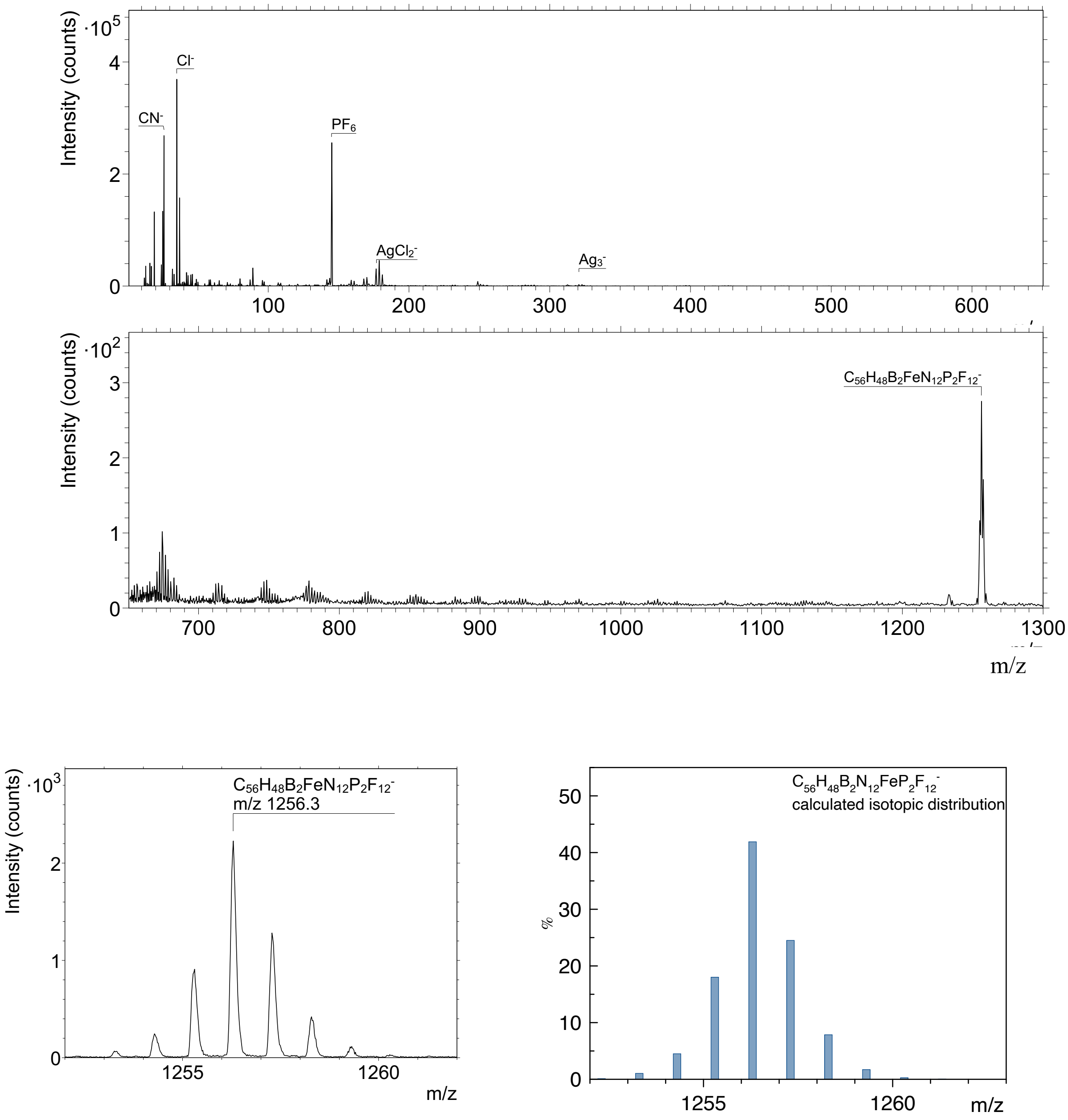

	Mass (u)	Description (ion charges omitted)
PF_6^-	144.9713	counter-ion
$C_{56}H_{48}B_2N_{12}FeP_2F_{12}^-$	1256.3021	$Fe(A)_2 \cdot 2PF_6$

Supplementary Fig. 31. Mass spectra of **2** and table of assignment. Negative polarity, substrate: Ag.

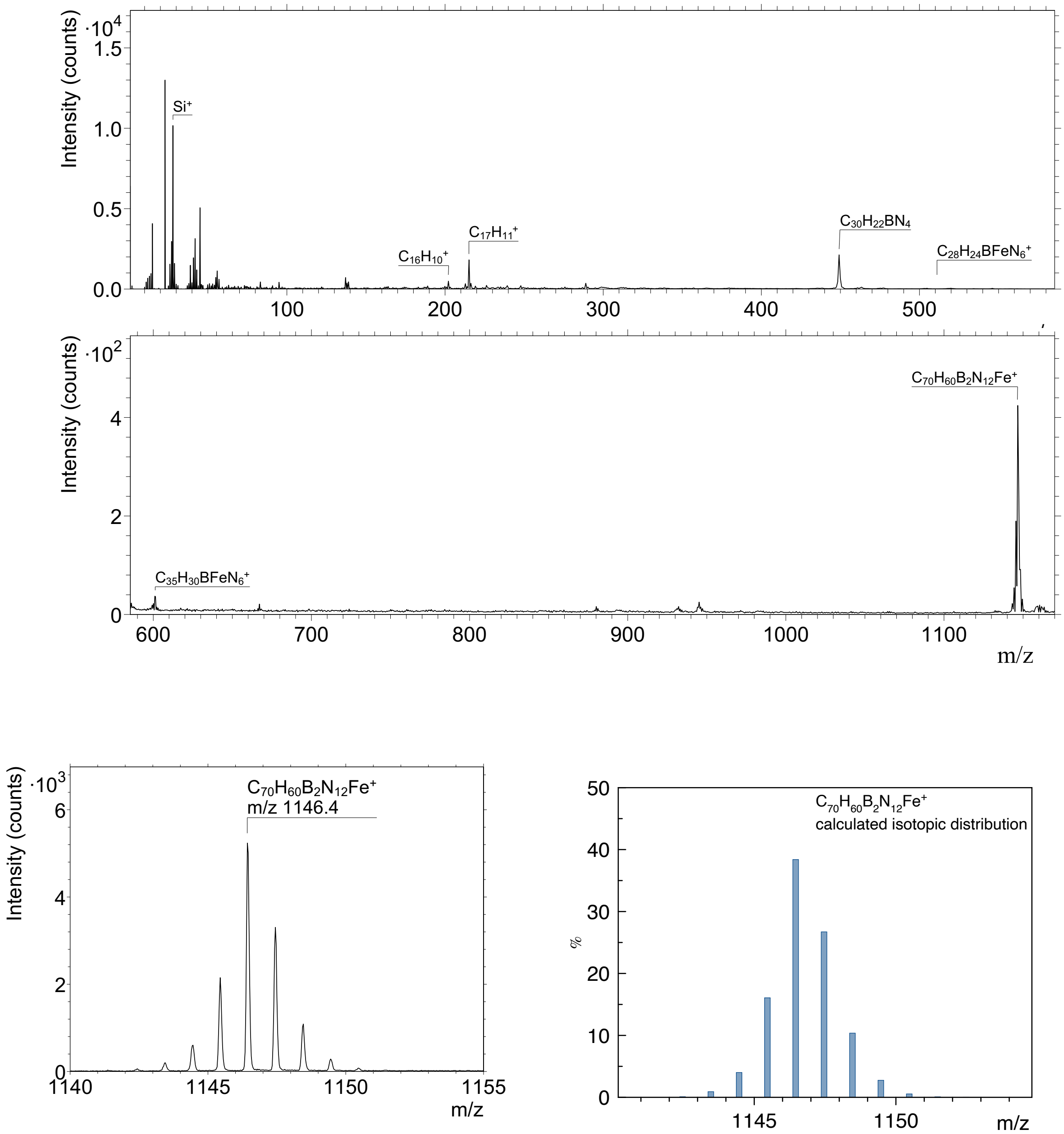

Ion	Mass (u)	Description (ion charges omitted)
$C_{16}H_{10}^+$	202.0774	pyrene
$C_{17}H_{11}^+$	215.0898	pyrene + CH
$C_{35}H_{30}BFeN_6^+$	601.2016	Fe(B)
$C_{70}H_{60}B_2N_{12}Fe^+$	1146.4485	$Fe(B)_2$

Supplementary Fig. 32. Mass spectra of complex **3** and table of assignment. Positive polarity, substrate: Si.

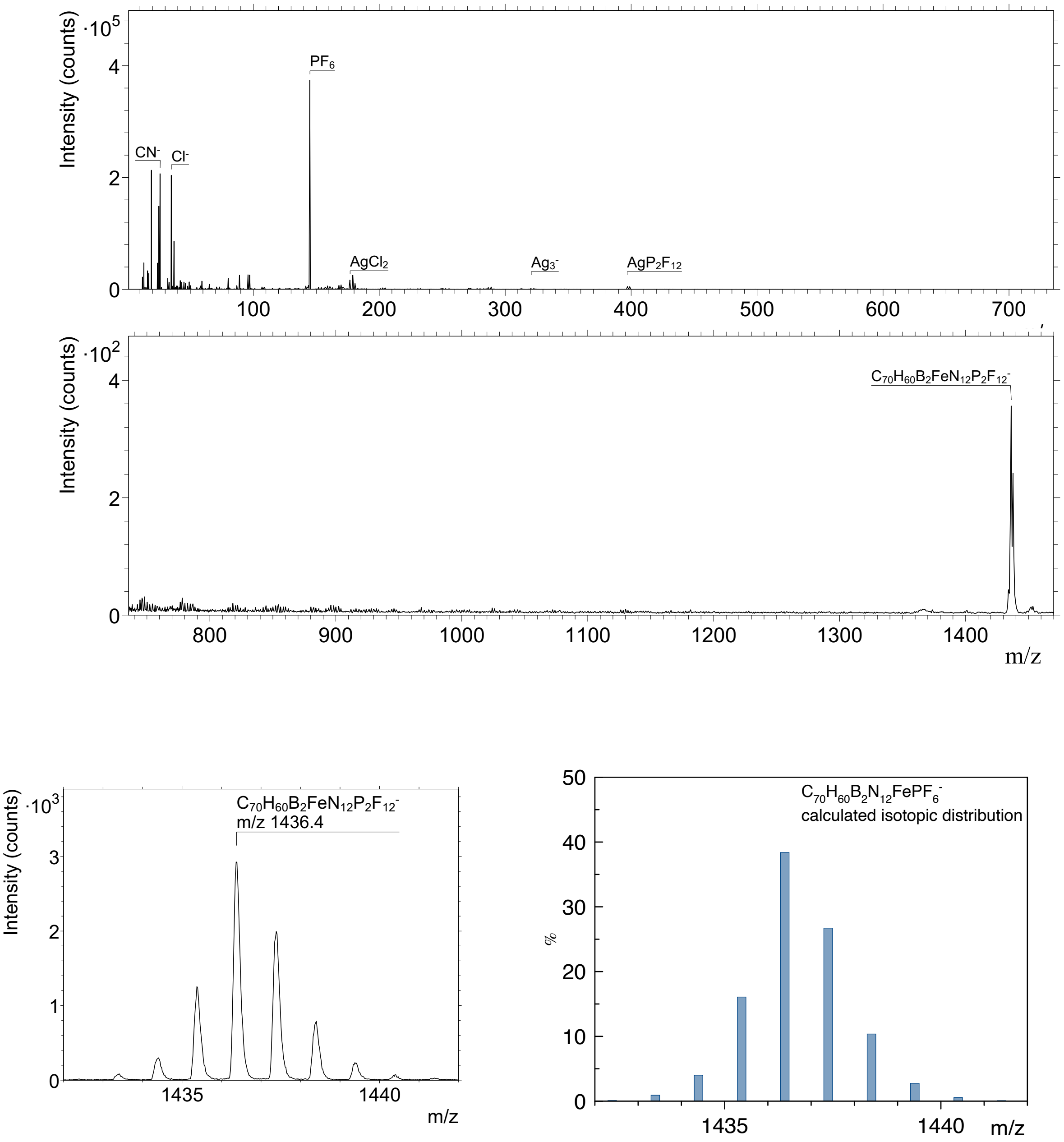

	Mass (u)	Description (ion charges omitted)
PF_6	144.9682	
$C_{70}H_{60}B_2$N12FePF6-	1436.3949	Fe(B2)·2PF_6

Supplementary Fig. 33. Mass spectra of complex **3** and table of assignment. Negative polarity, substrate: Ag.

Additional spectra and data

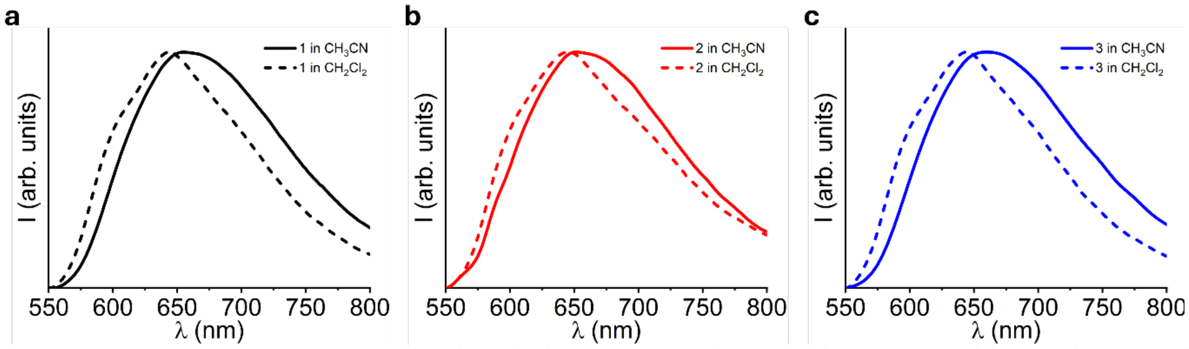

Supplementary Fig. 34. Normalized emission spectra of **1** (panel a), **2** (panel b), and **3** (panel c) in acetonitrile and dichloromethane fluid solution at room temperature. All spectra are independent of excitation wavelength. The spectra indicate positive solvatochromism.

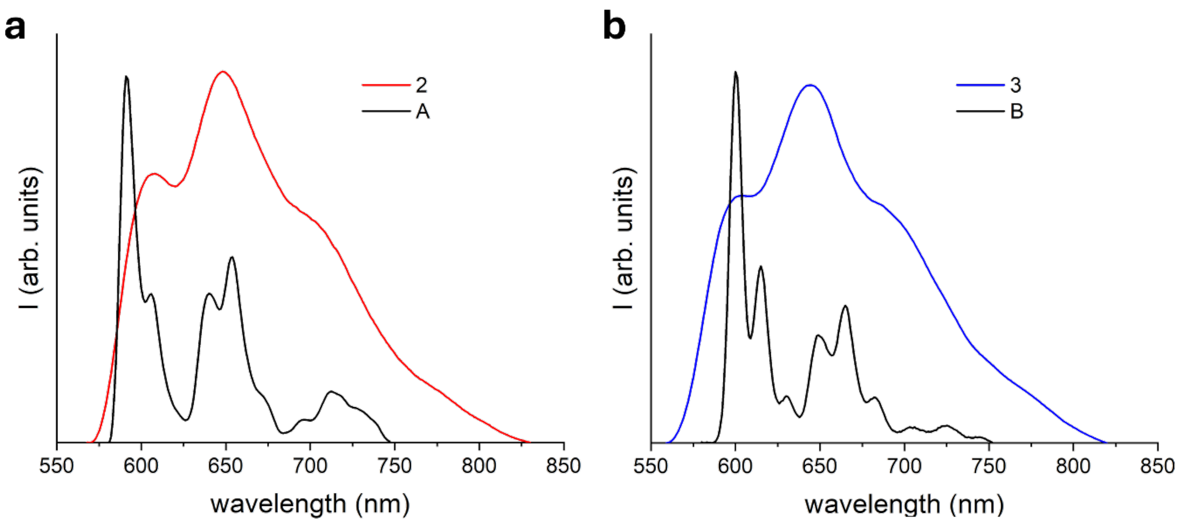

Supplementary Fig. 35. Emission spectra of **2** and of the free ligand **A** (panel a) and of **3** and of the free ligand **B** (panel b) in EtOH/MeOH 4:1 (v/v) at 77 K. 10 % of iodomethane has been added to **A** and **B** solutions to enhance intersystem crossing and phosphorescence.

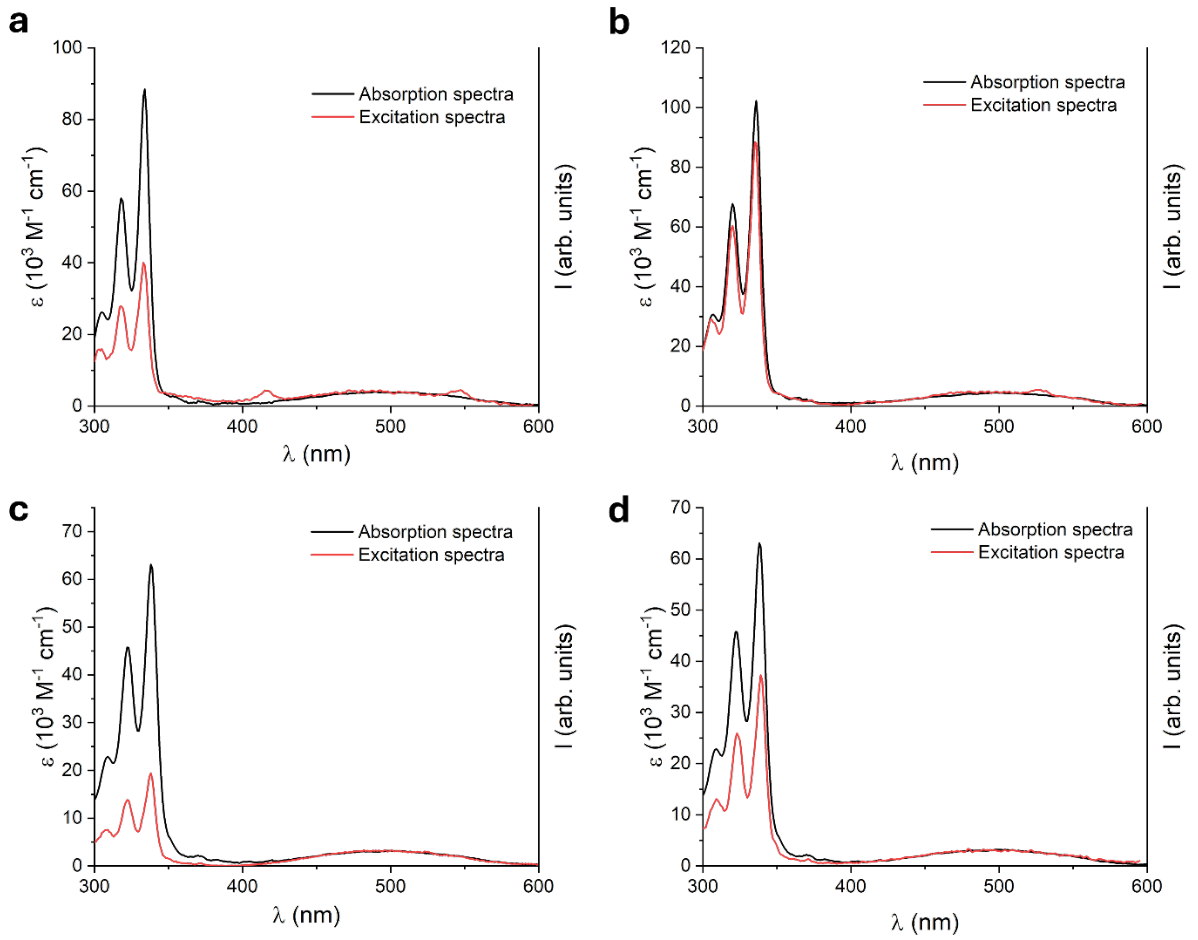

Supplementary Fig. 36. Excitation spectra (red) vs absorption spectra (black) of the complexes **2** in acetonitrile (panel a) and dichloromethane (panel b) and of complex **3** in acetonitrile (panel c) and dichloromethane (panel d). Emission wavelength, 670 nm.

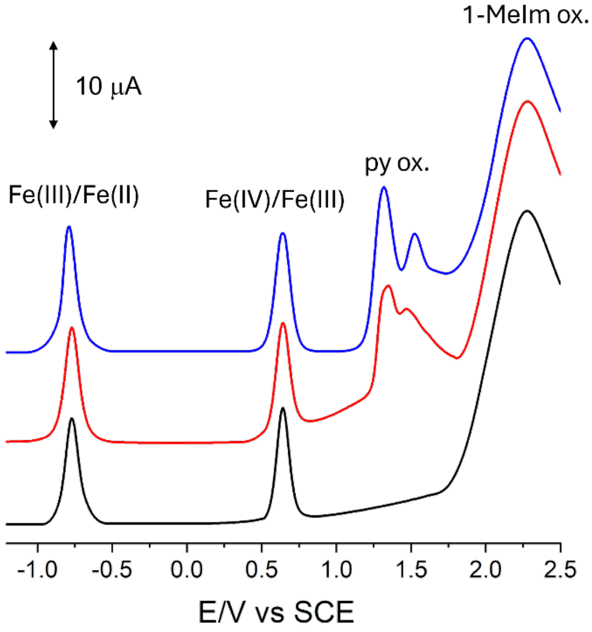

Supplementary Fig. 37. Differential pulse voltammogram of **1** (black), **2** (red), and **3** (blue) in acetonitrile (conc.: 0.5 mM; $TBAPF_6$: 0.05 M, scan rate 20 mV/s). Attribution of the various processes are indicated in figure.

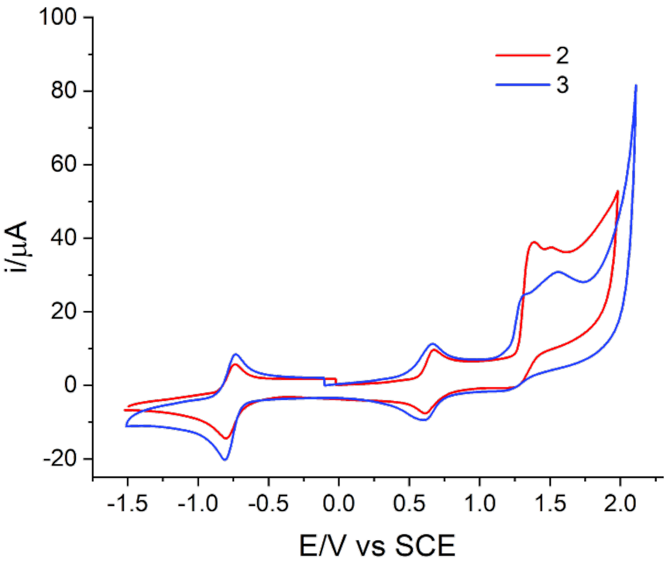

Supplementary Fig. 38. Cyclic voltammograms of **2** and **3** in acetonitrile (conc.: 0.5 mM; $TBAPF_6$: 0.05 M, scan rate 200 mV/s).

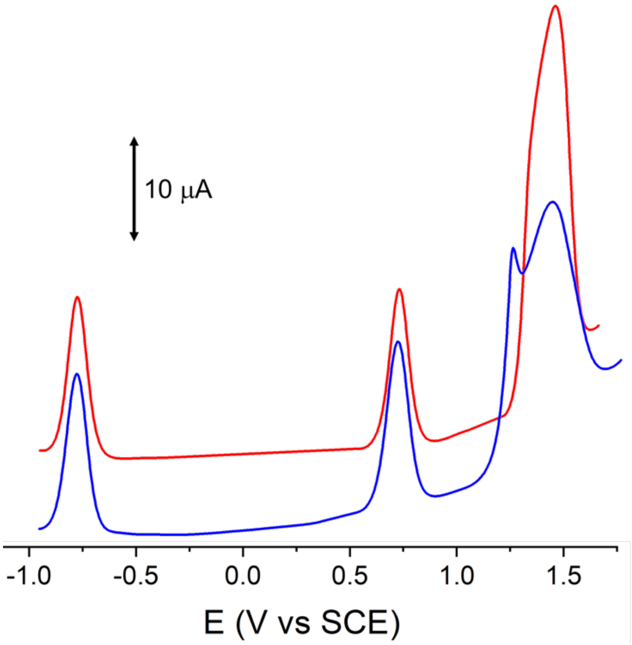

Supplementary Fig. 39. Differential pulse voltammogram of **2** (red) and **3** (blue) in 1,2-dichloroethane (conc.: 0.5 mM; TBAPF$_6$: 0.05 M, scan rate 20 mV/s).

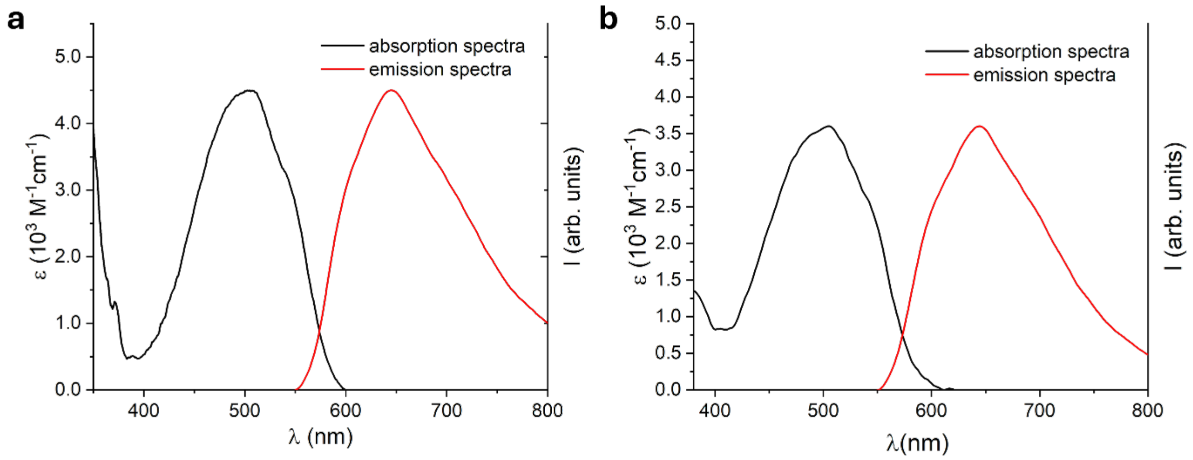

Supplementary Fig. 40. Absorption (black line) and emission (red line) spectra of **2** (panel a) and **3** (panel b) in dichloromethane. Crossing point allows to estimate the E_{00} (E^{00}) energy of the ^{2}LMCT state in this solvent, that is 2.16 eV for both complexes.

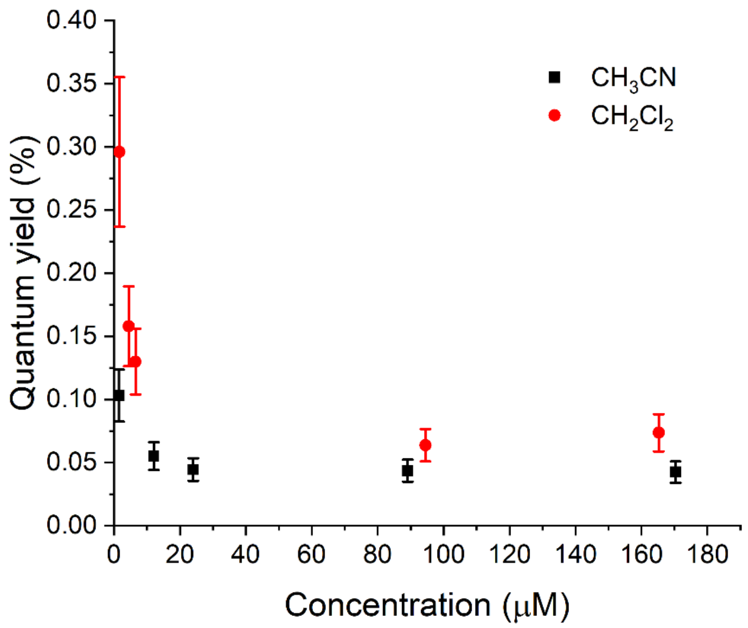

Supplementary Fig. 41. Concentration dependence of emission quantum yield of **2** in acetonitrile (black) and dichloromethane (red). Excitation wavelength, 456 nm.

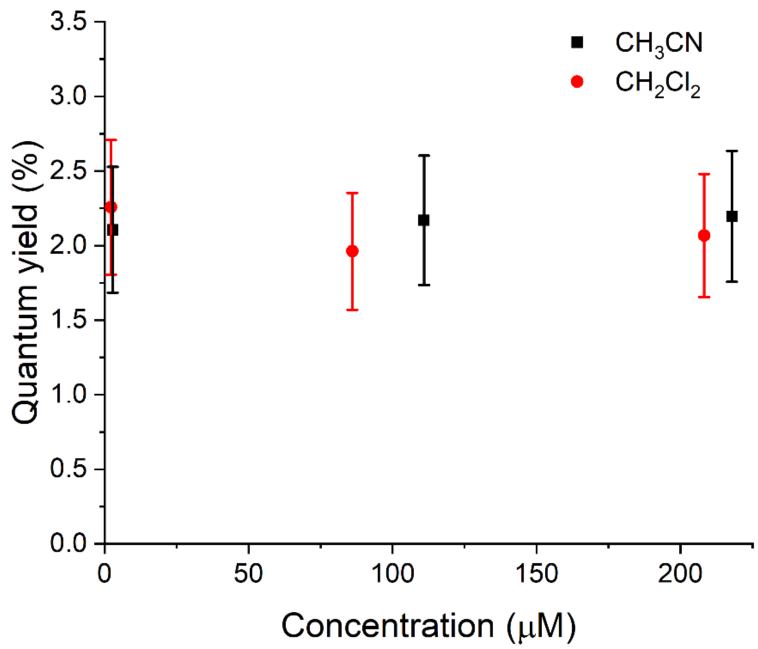

Supplementary Fig. 42. Concentration dependence of emission quantum yield of **3** in acetonitrile (black) and dichloromethane (red). Excitation wavelength, 456 nm.

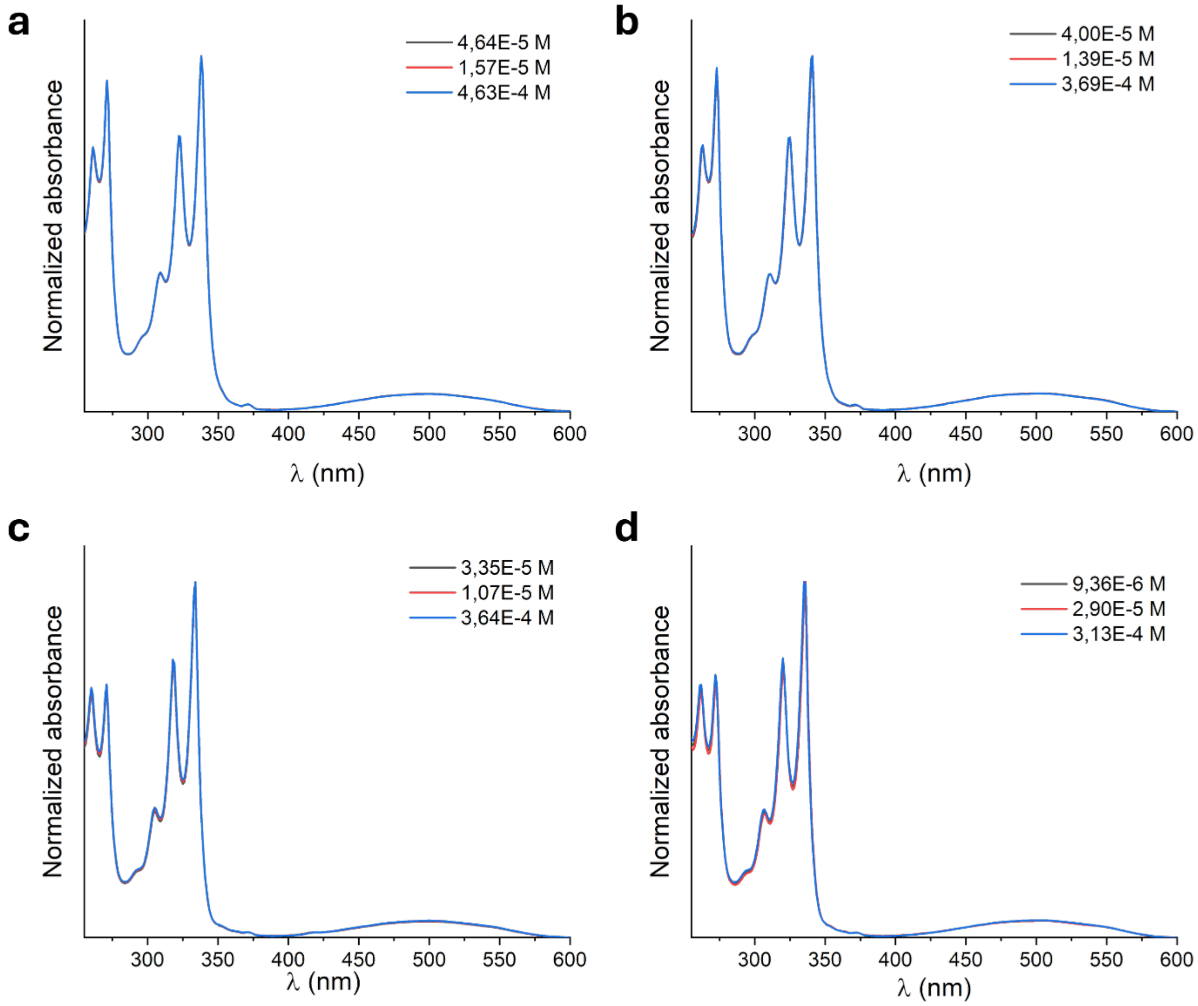

Supplementary Fig. 43. Concentration dependence of the absorption spectra of **2** in acetonitrile (panel a) and dichloromethane (panel b) and of **3** in acetonitrile (panel c) and dichloromethane (panel d). No changes in the absorption spectra with concentration is evidenced, even in the case of **2**, in which luminescence properties indicate static quenching and aggregation. Apparently, aggregation is not strong enough to modify the absorption properties.

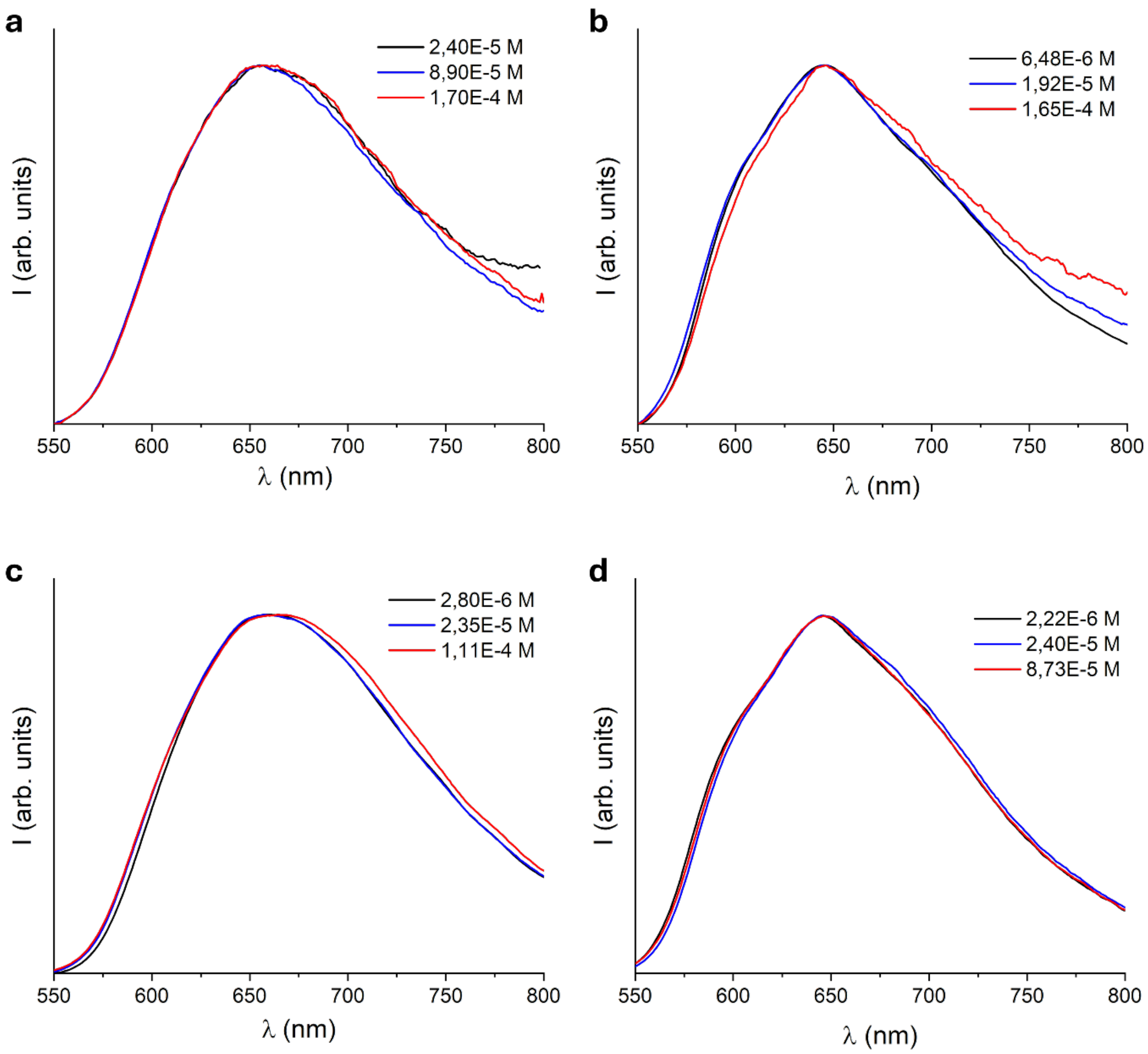

Supplementary Fig. 44. Concentration dependence of the normalized emission spectra of **2** in acetonitrile (panel a) and dichloromethane (panel b) and of **3** in acetonitrile (panel c) and dichloromethane (panel d).

Supplementary Table 1

Redox data of the new species **2** and **3** and of the model species **1** in acetonitrile (potential window: -1.2/+2.5 V). Reported data are vs SCE. Redox processes are reversible, unless otherwise stated. For reversible processes, $E_{1/2}$ is reported, whereas for irreversible processes the DPV peak is given. Attribution of the various processes to specific subunits are shown in **Supplementary Fig. 37**.

	E(red), V	E(ox), V		
**2**	-0.77	+0.64	+1.35[a,b]	+2.28[a]
**3**	-0.79	+0.64	+1.32[a,b]	+2.28[a]
**1**	-0.77	+0.64		+2.28[a]

(a) Irreversible process. (b) A further irreversible process follows this peak, probably involving the second pyrene subunit.

Supplementary Table 2

Redox data of the new species **2** and **3** in 1,2-dichloroethane (DCE). Reported data are vs SCE (potential window: -1.2/+1.7 V). Redox processes are reversible, unless otherwise stated. For reversible processes, $E_{1/2}$ is reported, whereas for irreversible processes the DPV peak is given.

	E(red), V	E(ox), V		
**2**	-0.77	+0.73	+1.33[a,b]	
**3**	-0.80	+0.71	+1.23[a,b]	

(a) Irreversible process. (b) A further irreversible process follows this peak, probably involving the second pyrene subunit.

Supplementary Discussion – Part 1: Luminescence and Photophysical Data

Calculation of the rate constants of ^{2}LMCT emission quenching reported in the paper
The quenching rate constant k_q is calculated according to the classical equation S1.

$$k_q = (1/\tau') - (1/\tau_0) \qquad (S1)$$

As far as **2** in acetonitrile is concerned, in eq. S1 τ' and τ_0 are the emission lifetimes of the shorter-lived emission component of **2**, 0.3 ns, and the emission lifetime of **1**, 2.0 ns, this latter assumed as the model for the emission lifetime of the ^{2}LMCT state of **2** in absence of quenching, respectively. In particular:

$$k_q = 1/(0.3\text{x}10^{-9}\ \text{s}) - 1/(2\text{x}10^{-9}\ \text{s}) = (3.3\text{x}10^9 - 0.5\text{x}10^9)\ \text{s}^{-1} = 2.8\text{x}10^9\ \text{s}^{-1}$$

As far as **3** in dichloromethane is concerned, in eq. S1 τ' and τ_0 are the emission lifetimes of the shorter-lived emission component of **3** (1.5 ns) and the emission lifetime of **1**, 2.4 ns, this latter assumed as the model for the emission lifetime of the ^{2}LMCT state of **3** in absence of quenching in dichloromethane, respectively. In particular:

$$k_q = 1/(1.5\text{x}10^{-9}\ \text{s}) - 1/(2.4\text{x}10^{-9}\ \text{s}) = (0.7\text{x}10^9 - 0.4\text{x}10^9)\ \text{s}^{-1} = 3\text{x}10^8\ \text{s}^{-1}$$

For **2** in dichloromethane, calculation yields:
$$k_q = 1/(0.8\text{x}10^{-9}\ \text{s}) - 1/(2.4\text{x}10^{-9}\ \text{s}) = (1.2\text{x}10^9 - 0.4\text{x}10^9)\ \text{s}^{-1} = 8\text{x}10^8\ \text{s}^{-1}$$

Excited-state equilibration and biexponential emission decay
Assuming k_a is the deactivation rate constant of a luminophore **P**, with k_a including both the radiative and radiationless decay rate constants of the isolated luminophore, when this luminophore is part of a multicomponent species including a subunit **Q** having an excited state lower-lying (or close) than the excited state of **P**, inter-component energy transfer from **P** to **Q** can take place. If the rate constant of such energy transfer, k_{et}, is much larger than k_a ($k_{et} >> k_a$), emission from **P** is totally quenched. If k_{et} is comparable with k_a, emission of **P** is only partially quenched and emission from **P** still occurs, with a reduced lifetime and quantum yield.

If the excited state of **Q** (the acceptor subunit of the energy transfer process) is close in energy to the emissive excited state of **P**, and the intrinsic decay of the excited state of **Q**, k_b, is slower than k_a and k_{et}, back energy transfer (with a rate constant k_{bet}) can take place. In these conditions, equilibration between the excited states of **P** and **Q** is obtained and the equilibrated state decays with a single lifetime, which depends on the population ratio between excited states of **P** and **Q** at the equilibrium, on its turn depending on k_a, k_b, and temperature (according to Boltzmann distribution). The luminescence output of the equilibrated state, neglecting the (eventual) luminescence of **Q**, is identical to the emission of **P**, but with a lifetime that is longer compared to the emission of the isolated **P**. As a consequence, the emission lifetime of **P** in the multicomponent **P-Q** species becomes biexponential (when k_a cannot be neglected in comparison to k_{et}), with a short component which is the partially quenched **P** emission and the long component corresponding to the lifetime of the equilibrated state.

The deactivation rate of the equilibrated state k_{eq} can be calculated according to the following equation: $k_{eq} = (1-\alpha)k_a + (\alpha)k_b$, in which α is the percentage of the excited state of **Q** and (1-α) is the percentage of the excited state of **P** at the equilibrium.

Summarizing, when $k_{et} >> k_a$, the short component is not present and the emission decay is monoexponential and corresponds to the lifetime of the equilibrated state. When k_a cannot be neglected in comparison to k_{et}, biexponential emission decay takes place.
As far as emission quantum yield is concerned, if k_b is much smaller than k_a, the emission quantum yield of the equilibrated state is not far from that of the isolated **P** (but it cannot be larger).

A more detailed discussion is found in ref 1.

A clear experimental example of biexponential emission decays dealing with excited-state equilibration between a Ru(II) complex and an anthracene derivative subunit can be found in ref. 2.

The above discussion, usually assumed for equilibration driven by energy transfer, is also valid, with the same formalism, if **Q** quenches **P** by electron transfer, which is the case reported in the present article. In this case, k_b is charge recombination to the ground state, α is the percentage of the charge-separated state $\mathbf{P^+}$-$\mathbf{Q^-}$ (or $\mathbf{P^-}$ - $\mathbf{Q^+}$, as in the present case) and (1- α) is the percentage of the excited state of **P** at the equilibrium.

Calculation of the electronic matrix element for the pyrene-to-*Fe(III) electron transfer occurring in 3 in dichloromethane

Reorganization energy λ is obtained by eq. S2, assuming negligible the inner contribution λ_{in} (this is acceptable considering that large aromatic molecules like pyrene have small inner reorganization energies for electron transfer, and the same can be assumed valid for the Fe(III)-to-Fe(II) reduction, in which the added electron is located on a t_{2g} orbital)[3].

$$\lambda = \lambda_{out} = \frac{e^2}{4\pi\varepsilon_0}\left(\frac{1}{\epsilon_{op}} - \frac{1}{\epsilon_s}\right)\left(\frac{1}{2r_a} + \frac{1}{2r_b} - \frac{1}{r_{ab}}\right) \quad \text{(S2)}$$

In eq. S2, in which the reactants are considered spheres in a dielectric continuum, ϵ_{op} and ϵ_s are the optical and static dielectric constants of the solvent, r_a and r_b are the radii of the reactants, r_{ab} is the interreactant center-to-center distance, and ε_0 is the dielectric constant of vacuum. Assuming, by CPK models, 6.4 Å, 7.0 Å, and 7.7 Å for $2r_a$, $2r_b$, and r_{ab}, respectively, a value of 0.92 eV is obtained for λ.

According to the Marcus quadratic equation (eq. S3), in which the driving force ΔG^0 of the pyrene-to-*Fe(III) electron transfer is -0.13 eV (from electrochemical and photophysical data, see main text), an activation energy $\Delta G^{\neq}$ = 0.17 eV is calculated.

$$\Delta G^{\neq} = \left(\frac{\lambda}{4}\right)\left(1 + \frac{\Delta G^0}{\lambda}\right)^2 \quad \text{(S3)}$$

Within the framework of the Landau-Zener treatment of avoided crossing,[4] the rate constant of the electron transfer k_{el} can be expressed as in equation S4, in which k is the Boltzmann constant, with the hypothesis that the electronic matrix element H_{AB} is small (i.e. within the nonadiabatic limit). For k_{el} we here assumed to value of the experimentally measured quenching rate constant, neglecting the back reaction.

$$k_{el} = \nu_{el} \exp(-\Delta G^{\neq}/kT) \quad \text{(S4)}$$

$$\nu_{el} = \frac{2H_{AB}^2}{h}\left(\frac{\pi^3}{\lambda kT}\right)^{1/2} \qquad \text{(S5)}$$

From eq. S4, the value of the electronic frequency ν_{el} is calculated to be 2.07 x 10^{11} s^{-1}. This value is used in equation S5 [3,4], that finally yields the electronic matrix element H_{AB} driving the electron transfer in **3**, which is estimated to be 28 cm^{-1}.

Supplementary Discussion - Part 2. Computational Study

Computational Methods

All DFT and TD-DFT calculations were performed using the Gaussian16 program.[5] The long-range-corrected LC-PBE[6] functional was applied in conjunction with Pople 6-311+G(d) basis set,[7,8] adding d polarization functions nitrogen and boron heteroatoms. Iron atoms were described using the LANL2TZ+ basis set and corresponding effective core potential (ECP).[9,10] Implicit solvent effects (here dichoromethane) were included using IEFPCM (default in Gaussian16), the integral equation formalism variant of the polarizable continuum model.[11,12] Analytical second derivatives of the energy were calculated to verify the nature of each stationary point, to determine the harmonic vibrational frequencies, and to provide zero-point vibrational energy corrections. The thermal and entropic contributions to the free energies were also obtained using unscaled frequencies. Spectra were simulated by Gaussian convolution of the computed transitions using a FWHM of 0.333 eV.

Computational Insights in the excited-state equilibration

Compounds **1** to **3** were optimized following the methodology previously described, and vertical electronic excitations were computed. Absorption spectra for the phtmeimb and ligands **A** and **B** were also computed for comparison. Relative energies regarding the optimised excited states of ligands and compound **2** are collected in **Supplementary Table 3.** Results regarding the absorption energy, predicted oscillator strengths, and molecular orbital (MO) contribution are collected in **Supplementary Tables 4-11**, while the graphical representations of the absorption spectra of compounds **1-3** compared with their corresponding ligands are shown in **Supplementary Fig. 50**. Except for phtmeimb, all other ligands show a pyrene-centred $^1\pi$-π^* transition around 285 nm and lower forbidden $^3\pi$-π^* excitations, around 800 nm, with the same character.

For compounds **1**-**3**, due to the high symmetry of the systems, many MOs are degenerated. In the following the transitions will be classed considering the orbital character. Results show that for compounds **2** and **3**, the most intense absorption bands are mainly ligand centred transitions, specifically $^2\pi$-π^* centred on the pyrene subunits, at almost the same wavelength of corresponding isolated ligands. Due to the lower conjugation of phtmeimb ligand, this transition is not even found inside the first 25 excitations computed for compound **1**. However, a low intensity band around 300 nm was found instead, corresponding to a ^{2}LMCT between the imidazole rings and the iron centre. This transition corresponds to the lowest excited doublet state of the molecule (discharging dark MC transitions). This same ^{2}LMCT was found to be the lowest excited doublet state for compounds **2** and **3** but hidden in the intense π- π^* pyrene-centred band.

Several forbidden doublet-to-quartet transitions were found below these bands for compounds **1-3**, mainly corresponding to two types of excited states: pyrene-centred $^4\pi$- π^* with the same character as their corresponding ligands, and ^{4}MLCT between the iron centre and the imidazole rings. This same photophysical behaviour was predicted using the PBE0 exchange correlation functional.[13,14] A summary containing all excitation energies and character for each transition for compounds **1-3** is depicted in **Supplementary Fig. 45**.

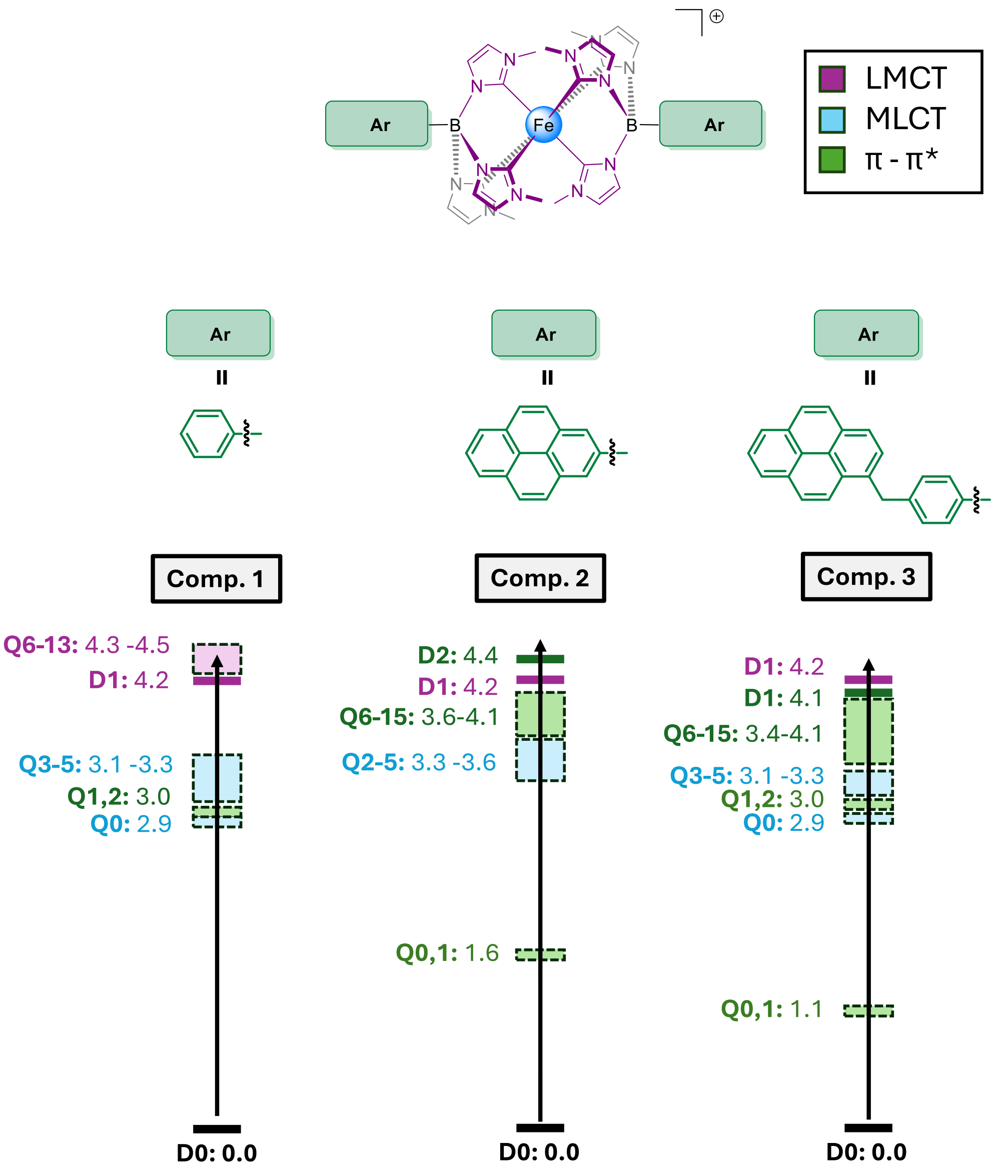

Supplementary Fig. 45. Energy diagram (in eV) for the first electronic transitions of compounds **1-3.** Full lines refer to doublet-to-doublet spin allowed transitions, while dashed lines refer to doublet to quartet excitations. States coloured according to the character of the transition.

Due to the size and complexity of the systems, compound **2** was selected to further investigate its photochemical behaviour. A molecular orbital diagram, containing the lowest electronic transitions, and the isocontour representation of each orbital is presented in **Supplementary Fig. 46**.

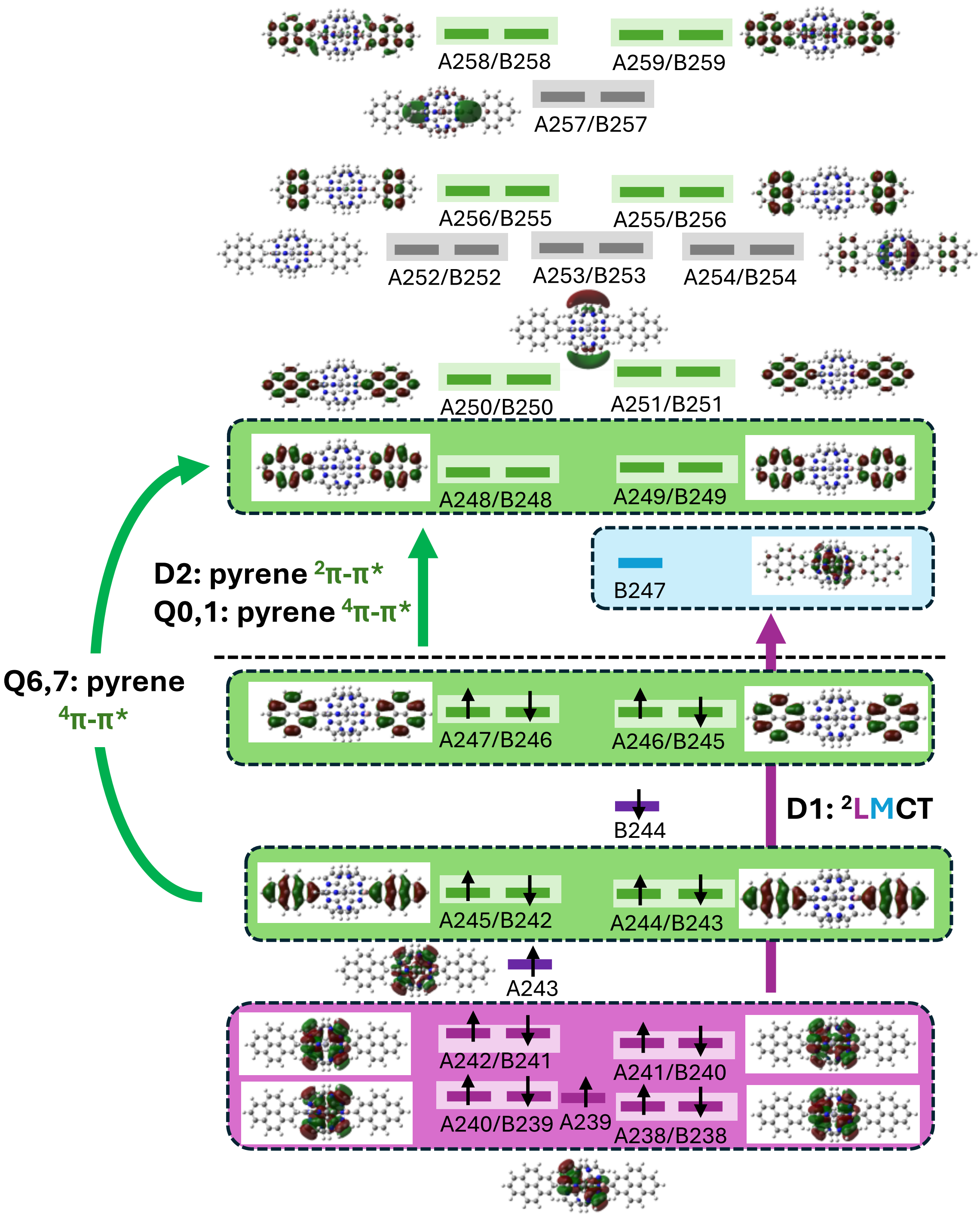

Supplementary Fig. 46. Frontier molecular orbitals for compound **2**, displaying the lowest doublet-to-doublet (**Dx**) and doublet to quartet (**Qx**) electronic transitions, along with the isocontour representation of the frontier orbitals (isoval.=0.02 a.u.). Colour code has been done according to **Supplementary Fig. 48.**

The optimization of the ^{2}LMCT state (so called **D1**) produced a stabilisation of 0.7 eV with respect to the ground state (GS) minimum. Comparing both geometries, only slightly smaller C-Fe distances and longer imidazole rings N-C bonds were found. In this new geometry, the GS, the $^2\pi$- π^* (**D2**) and the lowest $^4\pi$- π^* (**Q0**, **Q1**, **Q6** and **Q7**) were found to be 0.8 eV higher in energy. The same behaviour of these π-π^* states is consequence of their same photochemical character and underlines the complete decoupling of the ^{2}LMCT and the π- π^* transitions.

Although **Q0** and **Q1** correspond to the lower pyrene-centred quartets, these two states cannot take part in the excited-state equilibration due to their higher stability with respect to **D1**. Optimization of the higher pyrene-centred π- π* quartet **Q6** (isoenergetic with **Q7**) gave an energy gap of 3.3 eV with respect to the GS geometry, very close in energy to the optimised ^{2}LMCT state. Regarding these results, both Q6 and Q7 ^{4}π- π* pyrene centred excited states are proposed to be the ones taking part in the excited-state equilibration. Due to the different symmetry between these orbitals and those taking part in Q0 and Q1 transitions, the deactivation via these states is unlikely to happen. An energy diagram showing the lowest excited states of compound **2** at every given geometry, along with the nature of the transition is depicted in **Supplementary Fig. 47**. As depicted in **Supplementary Fig. 45**, both compounds **2** and **3** share a very close photochemical behaviour, therefore no major differences are expected regarding the excited state equilibration.

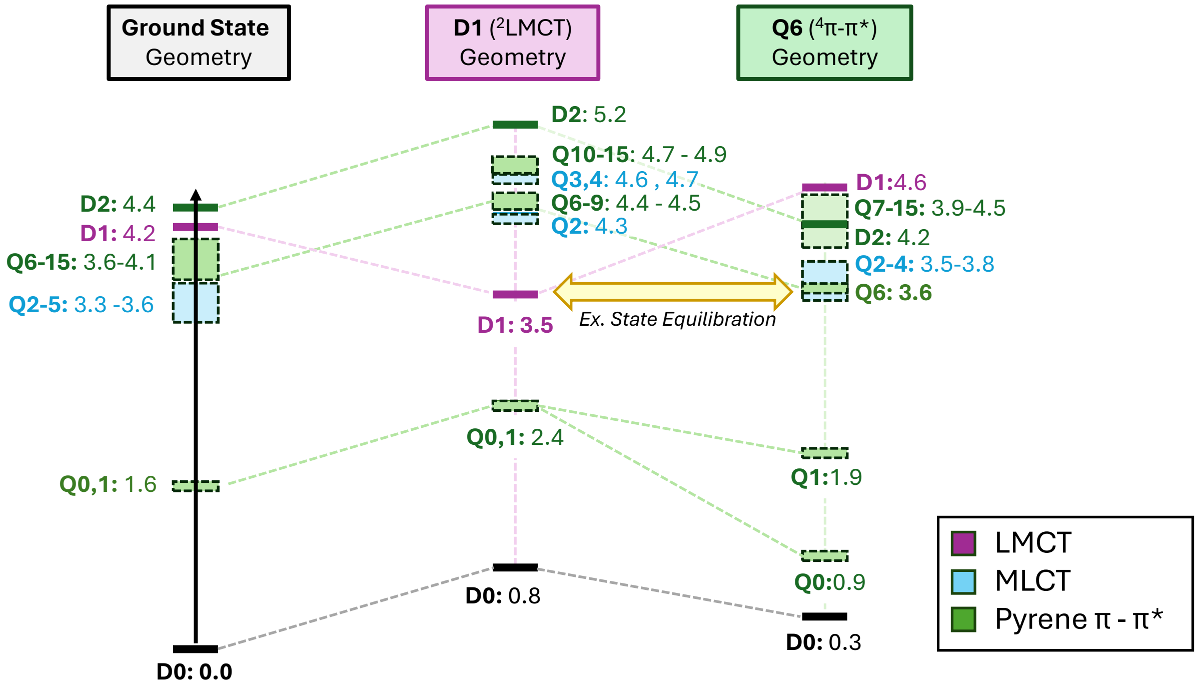

Supplementary Fig. 47. Energy diagram (energy units in eV) displaying the first excited states for compound **2** at the GS geometry, first doublet and sixth quartet excited states. Plane lines refer to doublet states while dashed ones refer to quartets. Each level has been coloured according to the nature of the transition.

Relative Energies

Relative energies regarding the optimised excited states of both ligands and compound **2** are collected in **Supplementary Table 3**.

Supplementary Table 3: Calculated relative energies (in kcal/mol and eV) and first vibrational frequencies for all located states of compound **2** and all ligands.

File Name	ΔE (kcal/mol)	ΔE_0 (kcal/mol)	ΔG (kcal/mol)	ΔE (eV)	Freq
**2**	0.0	0.0	0.0	0.00	11.0
**2-D1**	80.6	81.7	80.7	3.50	10.7
**2-Q6**	75.6	73.9	73.4	3.28	11.8

**2-Q7**	75.6	74.0	74.1	3.28	11.8
**phtmeimb**	0.0	0.0	0.0	0.0	41.7
**phtmeimb-T0**	82.8	79.5	77.8	3.6	18.3
**A**	0.0	0.0	0.0	0.0	21.4
**A-T0**	50.4	46.8	45.7	2.2	20.2
**B**	0.0	0.0	0.0	0.0	13.8
**B-T0**	48.9	45.3	43.8	2.1	10.5

TD-DFT Calculations

Predicted absorption energy, oscillator strengths, and orbitals involved in each transition, along with their corresponding contribution for each studied system are collected in **Supplementary Tables 4-11**. To simplify the analysis of the transitions, orbitals have been coloured considering their localization on the different moieties of the molecule following **Supplementary Fig. 48.**

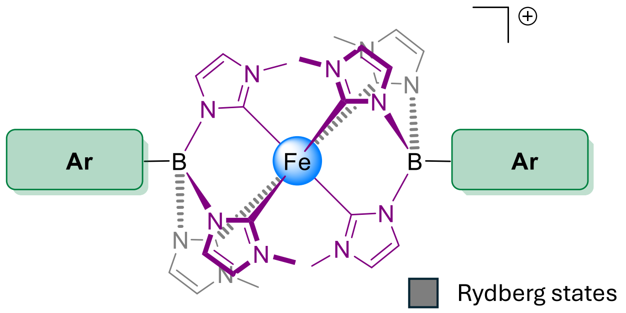

Supplementary Fig. 48. Colour code followed for the nomenclature of the orbitals involved in the electronic transitions.

Supplementary Table 4. Excitation energy (in nm), oscillator strength (f), total spin (S^2) and character in term of one electron excitations for the first 15 excitations of the **phtmeimb** ligand. Orbitals have been coloured according to their character as depicted in **Supplementary Fig. 48.** Spin allowed transitions are highlighted.

**phtmeimb**				
Ex. state	λ (nm)	f (a.u.)	S^2	Character
1	386	0.0	2.0	87A -> 93A<br>88A -> 92A<br>87B -> 93B<br>88B -> 89B<br>88B -> 92B
2	264	0.0	2.0	86A -> 89A<br>86B -> 89B
3	259	0.0	2.0	84A -> 90A<br>85A -> 90A<br>85A -> 99A<br>84B -> 90B<br>85B -> 90B

				85B -> 91B<br>85B -> 99B
4	258	0.0	2.0	84A -> 98A<br>86A -> 98A<br>84B -> 98B<br>86B -> 98B
5	255	0.0	2.0	85A -> 90A<br>85B -> 90B<br>85B -> 97B<br>85B -> 99B
6	255	0.0	2.0	84A -> 99A<br>86A -> 95A<br>86A -> 99A<br>84B -> 99B<br>86B -> 95B<br>86B -> 99B
7	254	0.0	2.0	84A -> 91A<br>86A -> 91A<br>84B -> 91B<br>86B -> 91B
8	252	0.0	2.0	87A -> 92A<br>87B -> 92B
9	249	0.0	2.0	87A -> 93A<br>88A -> 92A<br>87B -> 93B<br>88B -> 92B
10	236	0.0	2.0	88A -> 93A<br>88B -> 93B
11	218	0.0029	0.0	87A -> 92A<br>88A -> 93A<br>87B -> 92B<br>88B -> 93B
12	203	0.0	2.0	81A -> 89A<br>81B -> 89B
13	201	0.0	2.0	82A -> 91A<br>83A -> 90A<br>82B -> 91B<br>83B -> 90B
14	201	0.0	2.0	82A -> 90A<br>82B -> 90B
15	195	0.0245	0.0	87A -> 93A<br>88A -> 89A<br>88A -> 92A<br>87B -> 93B<br>88B -> 89B<br>88B -> 92B

Supplementary Table 5. Excitation energy (in nm), oscillator strength (f), total spin (S^2) and character in term of one electron excitations for the first 15 excitations of the ligand **A**. Orbitals have been coloured according to their character as depicted in **Supplementary Fig. 48**. Spin allowed transitions are highlighted.

**A**				
Ex. state	λ (nm)	f (a.u.)	S^2	Character
1	788	0.0000	2.000	120A -> 121A<br>120B -> 121B
2	343	0.0000	2.000	118A -> 121A<br>120A -> 126A<br>118B -> 121B<br>120B -> 126B
3	333	0.0000	2.000	117A -> 121A<br>117B -> 121B
4	322	0.0000	2.000	120A -> 122A<br>120B -> 122B
5	304	0.0000	2.000	119A -> 121A<br>119A -> 122A<br>119B -> 121B<br>119B -> 122B
6	300	0.0000	2.000	119A -> 121A<br>119A -> 122A<br>119B -> 121B<br>119B -> 122B
7	290	0.0418	0.000	119A -> 121A<br>120A -> 122A<br>119B -> 121B<br>120B -> 122B
8	284	0.6184	0.000	120A -> 121A<br>120B -> 121B
9	265	0.0000	2.000	115A -> 123A<br>116A -> 123A<br>115B -> 123B<br>116B -> 123B
10	259	0.0000	2.000	114A -> 124A<br>115A -> 124A<br>114B -> 124B<br>115B -> 124B
11	258	0.0000	2.000	116A -> 131A<br>114B -> 131A<br>116B -> 130B<br>116B -> 131B
12	255	0.0000	2.000	114A -> 124A<br>115A -> 123A<br>115A - > 124A<br>115A - > 131A<br>115A -> 124A

				114B -> 124B<br>115B -> 123B<br>115B -> 124B<br>115B -> 131B<br>115B - > 133B
13	255	0.0000	2.000	114A -> 133A<br>115A -> 128A<br>115A -> 132A<br>115A -> 134A<br>116A -> 128A<br>116A -> 132A<br>116A -> 133A<br>116A -> 134A<br>114B -> 133B<br>115B -> 128B<br>115B -> 132B<br>115B -> 134B<br>116B -> 128B<br>116B -> 132B<br>116B -> 133B<br>116B -> 134B
14	254	0.0000	2.000	114A -> 125A<br>116A -> 125A<br>114B -> 125B<br>116B -> 125B
15	247	0.0000	2.000	107A -> 121A<br>118A -> 126A<br>118A -> 127A<br>107B -> 121B<br>118B -> 126B

Supplementary Table 6. Excitation energy (in nm), oscillator strength (f), total spin (S^2) and character in term of one electron excitations for the first 15 excitations of the ligand **B**. Orbitals have been coloured according to their character as depicted in **Supplementary Fig. 48**. Spin allowed transitions are highlighted.

B				
Ex. state	λ (nm)	f (a.u.)	S^2	Character
1	810	0.0000	2.000	144A -> 145A<br>144B -> 145B
2	391	0.0000	2.000	140A -> 151A<br>142A -> 150A<br>140B -> 151B<br>142B -> 150B
3	344	0.0000	2.000	141A -> 145A<br>144A -> 153A<br>141B -> 145B<br>144B -> 153B

4	335	0.0000	2.000	139A -> 145A<br>144A -> 160A<br>139B -> 145B<br>144B -> 160B
5	315	0.0000	2.000	144A -> 147A<br>144B -> 147B
6	302	0.0000	2.000	143A -> 145A<br>143B -> 145B
7	297	0.0000	2.000	143A -> 147A<br>143B -> 147B
8	289	0.5651	0.000	144A -> 145A<br>144B -> 145B
9	286	0.1997	0.000	143A -> 145A<br>144A -> 145A<br>144A -> 147A<br>143B -> 145B<br>144B -> 145B<br>144B -> 147B
10	264	0.0000	2.000	136A -> 146A<br>137A -> 146A<br>138A -> 146A<br>136B -> 146B<br>137B -> 146B<br>138B -> 146B
11	259	0.0000	2.000	136A -> 148A<br>137A -> 148A<br>137A -> 149A<br>137A -> 155A<br>137A -> 156A<br>137A -> 158A<br>136B -> 148B<br>137B -> 148B<br>137B -> 149B<br>137B -> 155B<br>137B -> 156B<br>137B -> 158B
12	258	0.0000	2.000	136A -> 157A<br>138A -> 157A<br>136B -> 157B<br>138B -> 157B
13	255	0.0000	2.000	136A -> 148A<br>137A -> 148A<br>137A -> 156A<br>136B -> 148B<br>137B -> 148B<br>137B -> 156B
14	255	0.0000	2.000	136A -> 154A<br>136A -> 158A

				136A -> 159A<br>137A -> 154A<br>138A -> 154A<br>138A -> 158A<br>138A -> 159A<br>136B -> 154B<br>136B -> 158B<br>136B -> 159B<br>137B -> 154B<br>138B -> 154B<br>138B -> 158B<br>138B -> 159B
15	254	0.0000	2.000	136A -> 149A<br>138A -> 149A<br>136B -> 149B<br>138B -> 149B

Supplementary Table 7. Excitation energy (in nm), oscillator strength (f), total spin (S^2) and character in term of one electron excitations for the first 25 excitations of compound **1.** Orbitals have been coloured according to their character as depicted in **Supplementary Fig. 48**, mixed colour mean mixed character. Spin allowed transitions are highlighted.

1				
Ex. state	λ (nm)	f (a.u.)	S^2	Character
1	6670	0.0000	0.762	182B -> 183B
2	3881	0.0000	0.764	173B -> 183B<br>175B -> 183B
3	426	0.0000	2.728	182B -> 204B
4	408	0.0000	2.765	174A -> 187A<br>174A -> 188A<br>175A -> 187A<br>175A -> 188A<br>176A -> 185A<br>177A -> 185A<br>173B -> 188B<br>174B -> 187B<br>174B -> 188B<br>176B -> 185B<br>177B -> 185B
5	408	0.0000	2.765	174A -> 187A<br>174A -> 188A<br>175A -> 187A<br>175A -> 188A<br>176A -> 185A<br>177A -> 185A<br>173B -> 187B<br>173B -> 188B<br>174B -> 187B

				174B -> 188B
				176B -> 185B
6	401	0.0000	2.727	173A -> 200A
				173A -> 203A
				172B -> 204B
				173B -> 204B
				175B -> 204B
				182B -> 204B
7	400	0.0000	2.733	168A -> 204A
				183A -> 204A
				182B -> 208B
				182B -> 209B
8	376	0.0000	2.739	173A -> 204A
				173B -> 208B
				175B -> 208B
				175B -> 209B
9	316	0.0000	0.789	168A -> 204A
				173A -> 200A
				173A -> 203A
				183A -> 204A
				182B -> 204B
10	300	0.0000	0.786	168A -> 204A
				173A -> 204A
				183A -> 204A
				182B -> 208B
				182B -> 209B
11	293	0.0474	1.635	179B -> 183B
				180B -> 183B
12	290	0.0263	2.049	162B -> 183B
				178B -> 183B
				179B -> 183B
				180B -> 183B
13	289	0.0000	0.810	173A -> 193A
				173A -> 200A
				173A -> 203A
				175B -> 204B
				182B -> 204B
14	287	0.0000	2.512	181B -> 183B
15	286	0.0002	2.754	180A -> 199A
				180A -> 203A
				180A -> 209A
				182A -> 196A
				182A -> 205A
				183A -> 205A
				178B -> 201B
				179B -> 200B
				179B -> 201B
				179B -> 207B

				181B -> 202B<br>182B -> 202B
16	286	0.0000	2.727	180A -> 196A<br>180A -> 205A<br>180A -> 207A<br>182A -> 203A<br>183A -> 209A<br>179B -> 194B<br>179B -> 202B<br>181B -> 200B<br>182B -> 207B
17	282	0.0000	2.714	178A -> 192A<br>178A -> 201A<br>179A -> 205A<br>181A -> 198A<br>181A -> 208A<br>182A -> 209A<br>172B -> 200B<br>178B -> 202B<br>180B -> 197B<br>180B -> 206B<br>181B -> 207B
18	281	0.0053	2.547	178A -> 205A<br>179A -> 209A<br>181A -> 201A<br>182A -> 205A<br>179B -> 183B<br>180B -> 200B<br>180B -> 201B<br>181B -> 202B
19	279	0.0201	2.193	162B -> 183B<br>179B -> 183B<br>180B -> 183B
20	274	0.0000	2.270	181B -> 183B
21	268	0.0000	0.826	173A -> 204A
22	263	0.0000	1.002	156B -> 183B<br>158B -> 183B
23	260	0.0000	1.947	181A -> 202A<br>156B -> 183B<br>158B -> 183B<br>159B -> 183B<br>172B -> 183B<br>180B -> 199B
24	259	0.0004	1.722	162B -> 183B<br>168B -> 183B<br>178B -> 183B
25	259	0.0000	1.178	159B -> 183B

Supplementary Table 8. Excitation energy (in nm), oscillator strength (f), total spin (S^2) and character in term of one electron excitations for the first 25 excitations of compound **2.** Orbitals have been coloured according to their character as depicted in **Supplementary Fig. 48**, mixed colour mean mixed character. Spin allowed transitions are highlighted.

2				
Ex. state	λ (nm)	f (a.u.)	S^2	Character
1	6777	0.0000	0.761	244B -> 247B
2	4182	0.0000	0.762	237B -> 247B
3	787	0.0000	2.764	246A -> 248A<br>246A -> 249A<br>247A -> 248A<br>247A -> 249A<br>245B -> 248B<br>245B -> 249B<br>246B -> 248B<br>246B -> 249B
4	787	0.0000	2.764	246A -> 248A<br>246A -> 249A<br>247A -> 248A<br>247A -> 249A<br>245B -> 248B<br>245B -> 249B<br>246B -> 248B<br>246B -> 249B
5	378	0.0000	2.730	235A -> 273A<br>237B -> 275B<br>244B -> 275B<br>244B -> 283B
6	364	0.0000	2.722	228A -> 273A<br>235A -> 273A<br>243A -> 273A<br>237B -> 278B<br>237B -> 280B<br>244B -> 278B<br>244B -> 280B
7	358	0.0000	2.733	228A -> 274A<br>235A -> 273A<br>243A -> 274A<br>237B -> 275B<br>244B -> 278B<br>244B -> 283B
8	344	0.0000	2.738	235A -> 274A<br>237B -> 278B<br>237B -> 283B
9	343	0.0000	2.764	236A -> 248A<br>236A -> 249A<br>237A -> 248A

				237A -> 249A<br>246A -> 255A<br>246A -> 256A<br>247A -> 255A<br>247A -> 256A<br>234B -> 248B<br>234B -> 249B<br>235B -> 248B<br>235B -> 249B<br>245B -> 255B<br>245B -> 256B<br>246B -> 255B<br>246B -> 256B
10	343	0.0000	2.764	236A -> 248A<br>236A -> 249A<br>237A -> 248A<br>237A -> 249A<br>246A -> 255A<br>246A -> 256A<br>247A -> 255A<br>247A -> 256A<br>234B -> 248B<br>234B -> 249B<br>235B -> 248B<br>235B -> 249B<br>245B -> 255B<br>245B -> 256B<br>246B -> 255B<br>246B -> 256B
11	333	0.0000	2.764	233A -> 248A<br>233A -> 249A<br>234A -> 248A<br>234A -> 249A<br>246A -> 258A<br>246A -> 259A<br>247A -> 259A<br>232B -> 248B<br>232B -> 249B<br>233B -> 248B<br>233B -> 249B<br>245B -> 258B<br>245B -> 259B<br>246B -> 259B
12	333	0.0000	2.764	233A -> 248A<br>233A -> 249A<br>234A -> 248A<br>234A -> 249A<br>246A -> 259A

				247A	->	258A
				247A	->	259A
				232B	->	248B
				232B	->	249B
				233B	->	248B
				245B	->	259B
				246B	->	258B
				246B	->	259B
13	315	0.0000	2.764	246A	->	250A
				247A	->	251A
				245B	->	251B
				246B	->	250B
14	315	0.0000	2.764	246A	->	251A
				247A	->	250A
				245B	->	250B
				246B	->	251B
15	304	0.0000	2.764	244A	->	249A
				245A	->	248A
				242B	->	248B
				243B	->	249B
16	304	0.0000	2.764	244A	->	248A
				245A	->	249A
				242B	->	249B
				243B	->	248B
17	303	0.0000	2.763	244A	->	250A
				245A	->	251A
				242B	->	250B
				243B	->	251B
18	303	0.0000	2.764	244A	->	251A
				245A	->	250A
				242B	->	251B
				243B	->	250B
19	293	0.1386	1.308	240B	->	247B
20	292	0.0000	0.786	228A	->	274A
				235A	->	273A
				243A	->	274A
				244B	->	275B
				244B	->	283B
21	288	0.0050	0.766	244A	->	249A
				245A	->	248A
				246A	->	250A
				247A	->	251A
				242B	->	248B
				243B	->	249B
				245B	->	251B
				246B	->	250B
22	288	0.0000	0.764	244A	->	248A
				245A	->	249A

				246A -> 251A<br>247A -> 250A<br>242B -> 249B<br>243B -> 248B<br>245B -> 250B<br>246B -> 251B
23	287	0.0423	1.399	239B -> 247B
24	284	1.4976	0.771	246A -> 249A<br>247A -> 248A<br>245B -> 249B<br>246B -> 248B
25	283	0.0000	0.764	246A -> 248A<br>247A -> 249A<br>245B -> 248B<br>246B -> 249B

Supplementary Table 9. Excitation energy (in nm), oscillator strength (f), total spin (S^2) and character in term of one electron excitations for the first 30 excitations of compound **3.** Orbitals have been coloured according to their character as depicted in **Supplementary Fig. 48**, mixed colour mean mixed character. Spin allowed transitions are highlighted.

3				
Ex. state	λ (nm)	f (a.u.)	S^2	Character
1	6676	0.0000	0.762	292B -> 297B
2	3871	0.0000	0.764	283B -> 297B
3	1092	0.0000	2.765	294A -> 296A<br>294A -> 297A<br>295A -> 296A<br>295A -> 297A<br>293B -> 295B<br>293B -> 296B<br>294B -> 295B<br>294B -> 296B
4	1092	0.0000	2.765	294A -> 296A<br>294A -> 297A<br>295A -> 296A<br>295A -> 297A<br>293B -> 295B<br>293B -> 296B<br>294B -> 295B<br>294B -> 296B
5	426	0.0000	2.728	273B -> 325B<br>292B -> 325B
6	414	0.0000	2.765	282A -> 303A<br>282A -> 304A<br>283A -> 303A<br>283A -> 304A<br>284A -> 300A

				284A -> 301A<br>285A -> 300A<br>285A -> 301A<br>281B -> 303B<br>281B -> 304B<br>282B -> 303B<br>282B -> 304B<br>284B -> 300B<br>284B -> 301B<br>285B -> 300B<br>285B -> 301B
7	414	0.0000	2.765	282A -> 303A<br>282A -> 304A<br>283A -> 303A<br>283A -> 304A<br>284A -> 300A<br>284A -> 301A<br>285A -> 300A<br>285A -> 301A<br>281B -> 303B<br>281B -> 304B<br>282B -> 303B<br>282B -> 304B<br>284B -> 300B<br>284B -> 301B<br>285B -> 300B<br>285B -> 301B
8	402	0.0000	2.726	277A -> 320A<br>277A -> 323A<br>283B -> 325B<br>292B -> 325B<br>292B -> 328B<br>292B -> 329B
9	400	0.0000	2.733	272A -> 324A<br>291A -> 324A<br>283B -> 325B<br>292B -> 328B
10	377	0.0000	2.739	277A -> 324A<br>283B -> 328B
11	361	0.0000	2.765	280A -> 296A<br>280A -> 297A<br>281A -> 296A<br>281A -> 297A<br>294A -> 307A<br>294A -> 308A<br>295A -> 307A<br>295A -> 308A<br>279B -> 295B

				279B -> 296B<br>280B -> 295B<br>280B -> 296B<br>293B -> 307B<br>293B -> 308B<br>294B -> 307B<br>294B -> 308B
12	361	0.0000	2.765	280A -> 296A<br>280A -> 297A<br>281A -> 296A<br>281A -> 297A<br>294A -> 307A<br>294A -> 308A<br>295A -> 307A<br>295A -> 308A<br>279B -> 295B<br>279B -> 296B<br>280B -> 295B<br>280B -> 296B<br>293B -> 307B<br>293B -> 308B<br>294B -> 307B<br>294B -> 308B
13	348	0.0000	2.765	278A -> 296A<br>278A -> 297A<br>279A -> 296A<br>279A -> 297A<br>294A -> 315A<br>294A -> 316A<br>295A -> 315A<br>295A -> 316A<br>276B -> 295B<br>276B -> 296B<br>277B -> 295B<br>277B -> 296B<br>293B -> 313B<br>294B -> 313B
14	348	0.0000	2.765	278A -> 296A<br>278A -> 297A<br>279A -> 296A<br>279A -> 297A<br>294A -> 315A<br>294A -> 316A<br>295A -> 315A<br>295A -> 316A<br>276B -> 295B<br>276B -> 296B<br>277B -> 295B

				277B -> 296B<br>293B -> 313B<br>294B -> 313B
15	329	0.0000	2.765	294A -> 298A<br>294A -> 299A<br>295A -> 298A<br>295A -> 299A<br>293B -> 298B<br>293B -> 299B<br>294B -> 298B<br>294B -> 299B
16	329	0.0000	2.765	294A -> 298A<br>294A -> 299A<br>295A -> 298A<br>295A -> 299A<br>293B -> 298B<br>293B -> 299B<br>294B -> 298B<br>294B -> 299B
17	316	0.0000	0.789	277A -> 320A<br>277A -> 323A<br>291A -> 324A<br>283B -> 325B<br>292B -> 325B
18	315	0.0000	2.765	292A -> 296A<br>292A -> 297A<br>293A -> 296A<br>293A -> 297A<br>290B -> 295B<br>290B -> 296B<br>291B -> 295B<br>291B -> 296B
19	315	0.0000	2.765	292A -> 296A<br>292A -> 297A<br>293A -> 296A<br>293A -> 297A<br>290B -> 295B<br>290B -> 296B<br>291B -> 295B<br>291B -> 296B
20	306	0.0000	2.765	292A -> 298A<br>292A -> 299A<br>293A -> 298A<br>293A -> 299A<br>290B -> 298B<br>290B -> 299B<br>291B -> 298B<br>291B -> 299B

21	306	0.0000	2.765	292A -> 298A<br>292A -> 299A<br>293A -> 298A<br>293A -> 299A<br>290B -> 298B<br>290B -> 299B<br>291B -> 298B<br>291B -> 299B
22	302	1.4098	0.766	294A -> 297A<br>295A -> 296A<br>293B -> 295B<br>294B -> 296B
23	302	0.0000	0.765	294A -> 296A<br>295A -> 297A<br>293B -> 296B<br>294B -> 295B
24	300	0.0000	0.786	277A -> 324A<br>291A -> 324A
25	299	0.2582	0.766	292A -> 297A<br>293A -> 296A<br>294A -> 299A<br>295A -> 298A<br>290B -> 295B<br>291B -> 296B<br>293B -> 299B
26	299	0.0000	0.765	292A -> 296A<br>293A -> 297A<br>294A -> 298A<br>295A -> 299A<br>290B -> 296B<br>291B -> 295B<br>293B -> 298B<br>294B -> 299B
27	293	0.0351	1.632	288B -> 297B
28	290	0.0232	2.049	287B -> 297B<br>288B -> 297B
29	289	0.0000	0.800	277A -> 320A<br>277A -> 323A<br>283B -> 325B
30	287	0.0000	2.523	289B -> 297B

Supplementary Table 10. Excitation energy (in nm), oscillator strength (f), total spin (S^2) and character in term of one electron excitations for the first 25 excitations of compound **2** first optimized doublet (**2-D1**). Orbitals have been coloured according to their character as depicted in **Supplementary Fig. 48**, mixed colour mean mixed character. Spin allowed transitions are highlighted.

2-D1

Ex. state	λ (nm)	f (a.u.)	S^2	Character
1	9621	0.0000	0.775	243B -> 247B
2	3298	0.0000	0.782	237B -> 247B
3	787	0.0000	2.785	246A -> 248A<br>245B -> 248B
4	787	0.0000	2.785	247A -> 249A<br>246B -> 249B
5	459	0.0696	1.729	244B -> 247B
6	355	0.0000	2.760	242A -> 274A<br>243B -> 276B<br>243B -> 283B
7	343	0.0000	2.785	237A -> 249A<br>247A -> 255A<br>235B -> 249B<br>246B -> 255B<br>246B -> 256B
8	343	0.0000	2.785	236A -> 248A<br>246A -> 255A<br>234B -> 248B<br>245B -> 255B
9	333	0.0000	2.785	233A -> 248A<br>246A -> 261A<br>232B -> 248B<br>245B -> 259B
10	333	0.0000	2.785	234A -> 249A<br>247A -> 259A<br>233B -> 249B<br>246B -> 258B
11	326	0.0000	2.763	235A -> 274A<br>237B -> 276B<br>237B -> 283B
12	321	0.0002	2.730	243B -> 282B<br>243B -> 286B
13	315	0.0000	2.785	246A -> 250A<br>245B -> 250B
14	315	0.0000	2.785	247A -> 251A<br>246B -> 251B
15	313	0.0006	2.739	237B -> 282B<br>237B -> 286B
16	304	0.0000	2.785	243A -> 249A<br>244A -> 249A<br>242B -> 249B
17	304	0.0000	2.785	243A -> 248A<br>241B -> 248B
18	303	0.0000	2.784	244A -> 251A<br>242B -> 251B
19	303	0.0000	2.784	243A -> 250A

				241B -> 250B
20	298	0.1254	1.681	245A -> 257A<br>221B -> 247B<br>240B -> 247B
21	297	0.0159	1.100	239B -> 247B
22	288	0.0021	0.785	246A -> 250A<br>245B -> 250B
23	288	0.0020	0.785	247A -> 251A<br>246B -> 251B
24	285	0.5082	1.889	245A -> 268A<br>246A -> 248A<br>240B -> 247B<br>245B -> 248B
25	284	1.0487	1.103	247A -> 249A<br>246B -> 249B

Supplementary Table 11. Excitation energy (in nm), oscillator strength (f), total spin (S^2) and character in term of one electron excitations for the first 25 excitations of compound **2** sixth optimized quartet (**2-Q6**). Orbitals have been coloured according to their character as depicted in **Supplementary Fig. 48**, mixed colour mean mixed character. Spin allowed transitions are highlighted.

**2-Q6**				
Ex. state	λ (nm)	f (a.u.)	S^2	Character
1	7811	0.0000	0.761	244B -> 249B
2	4908	0.0000	0.762	237B -> 249B
3	2135	0.0000	2.763	247A -> 248A<br>246B -> 247B
4	786	0.0000	2.763	246A -> 249A<br>245B -> 248B
5	386	0.0000	2.728	245A -> 273A<br>244B -> 276B<br>244B -> 284B
6	371	0.0000	2.764	237A -> 248A<br>247A -> 253A<br>236B -> 247B<br>246B -> 253B
7	366	0.0000	2.731	235A -> 273A<br>237B -> 276B
8	365	0.0000	2.724	229A -> 273A<br>235A -> 273A<br>235A -> 275A<br>245A -> 273A<br>237B -> 279B<br>237B -> 282B<br>244B -> 279B<br>244B -> 282B
9	349	0.0000	2.737	235A -> 275A<br>237B -> 284B

10	348	0.0000	2.763	233A -> 248A<br>234A -> 248A<br>247A -> 258A<br>232B -> 247B<br>233B -> 247B<br>246B -> 258B
11	343	0.0000	2.764	236A -> 249A<br>246A -> 256A<br>234B -> 248B<br>245B -> 256B
12	333	0.0000	2.763	247A -> 251A<br>246B -> 251B
13	321	0.0000	2.763	233A -> 249A<br>234A -> 249A<br>246A -> 259A<br>232B -> 248B<br>233B -> 248B<br>245B -> 259B
14	319	0.0000	2.763	243A -> 248A<br>242B -> 247B
15	315	1.0298	0.764	247A -> 248A<br>246B -> 247B
16	315	0.0000	2.763	246A -> 250A<br>245B -> 250B
17	305	0.0017	0.764	243A -> 248A<br>247A -> 251A<br>242B -> 247B<br>246B -> 251B
18	304	0.0000	2.763	244A -> 249A<br>243B -> 248B
19	303	0.0000	2.763	244A -> 250A<br>243B -> 250B
20	296	0.0000	0.789	235A -> 273A<br>245A -> 273A<br>245A -> 275A<br>244B -> 276B<br>244B -> 284B
21	294	0.0000	2.763	243A -> 251A<br>242B -> 251B
22	290	0.0487	1.280	239B -> 249B
23	288	0.0020	0.764	244A -> 249A<br>246A -> 250A<br>243B -> 248B
24	286	0.1117	1.486	240B -> 249B
25	283	0.6697	0.785	246A -> 249A<br>245B -> 248B

Predicted absorption spectra coming from previous displayed transitions for each species are collected in **Supplementary Figs. 49-50**.

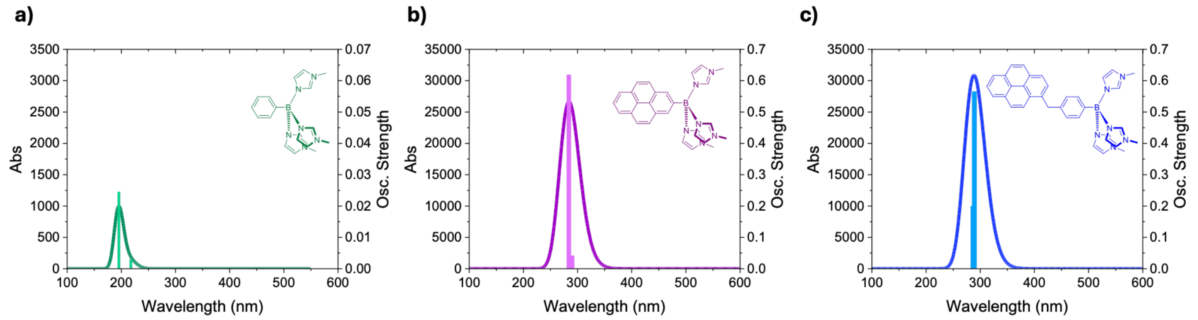

Supplementary Fig. 49: Predicted absorption spectra of ligands a) **phtmeimb**, b) **A**, c) **B**. Notice that for **phtmeimb** ligand (green trace) the units of both absorption and oscillator strength has been divided by a factor of 10 due to its low absorption rate.

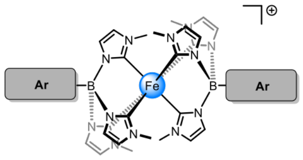

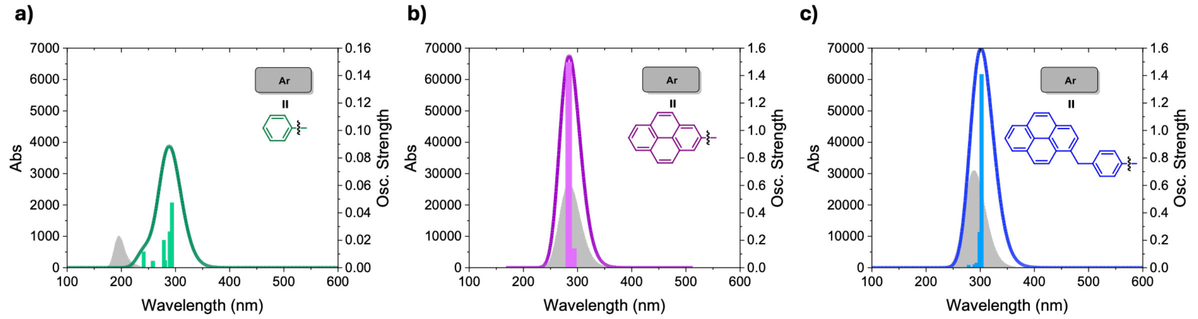

Supplementary Fig. 50. Predicted absorption spectra of compounds a) **1**, b) **2**, c) **3**, along with the comparison with their corresponding ligand (grey shadow). Notice that for compound **1** (green trace) the units of both absorption and oscillator strength have been divided by a factor of 10 due to its low absorption rate.

Molecular Orbital Representations

The isocontour representation of the molecular orbitals (MOs) most commonly involved in the electronic transitions described in **Supplementary Tables 4-11** for every studied system are depicted in **Supplementary Figs. 51-58**.

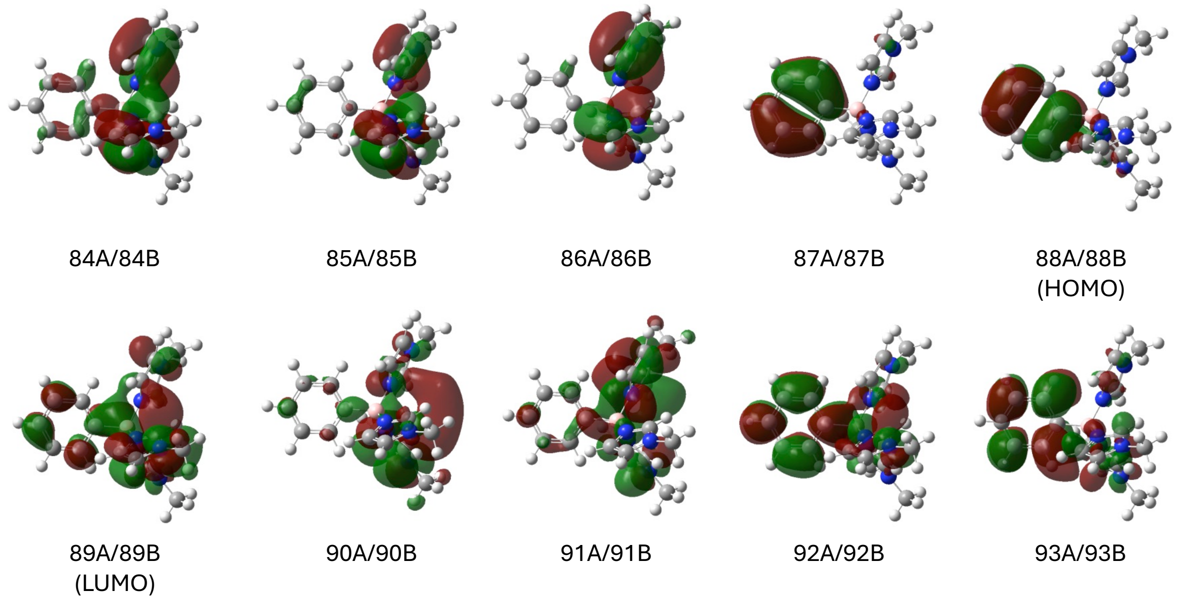

Supplementary Fig. 51. Isocontour representation (isovalue 0.02 a.u.) for the MOs mainly involved in the first 15 electronic excitations of ligand **phtmeimb**.

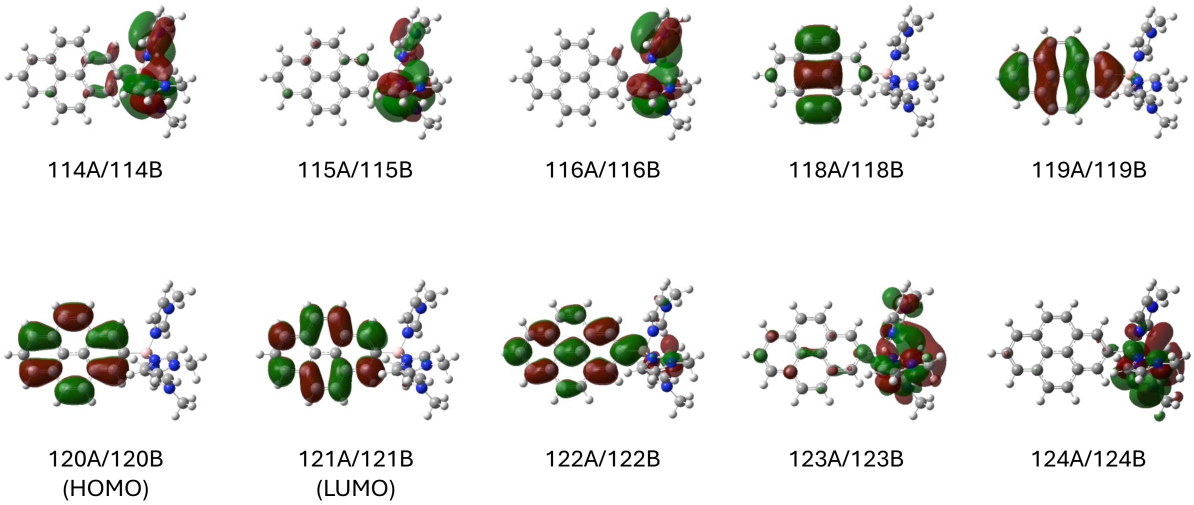

Supplementary Fig. 52. Isocontour representation (isovalue 0.02 a.u.) for the MOs mainly involved in the first 15 electronic excitations of ligand **A**.

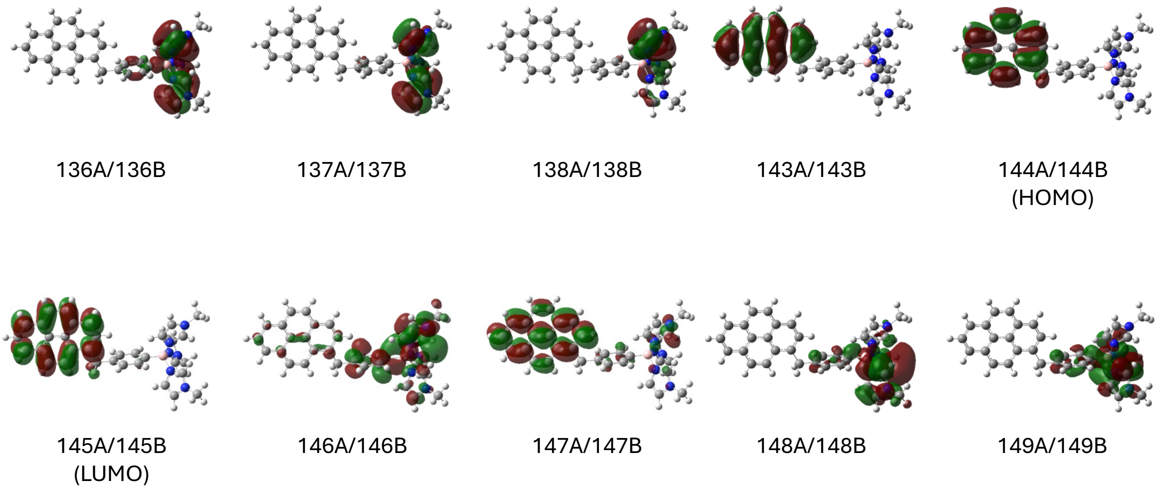

Supplementary Fig. 53. Isocontour representation (isovalue 0.02 a.u.) for the MOs mainly involved in the first 15 electronic excitations of ligand **B.**

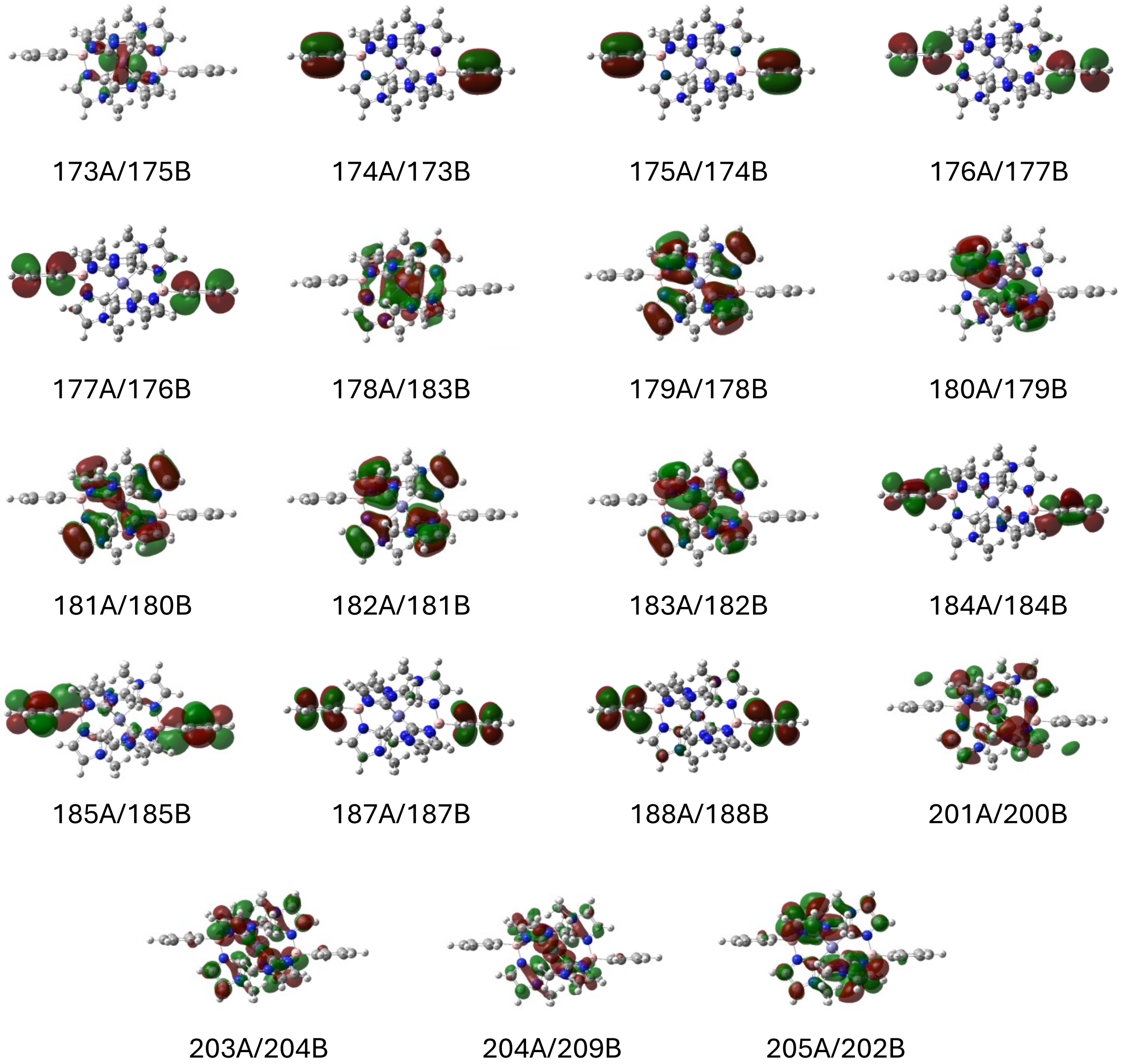

Supplementary Fig. 54. Isocontour representation (isovalue 0.02 a.u.) for the MOs mainly involved in the first 25 electronic excitations of compound **1**.

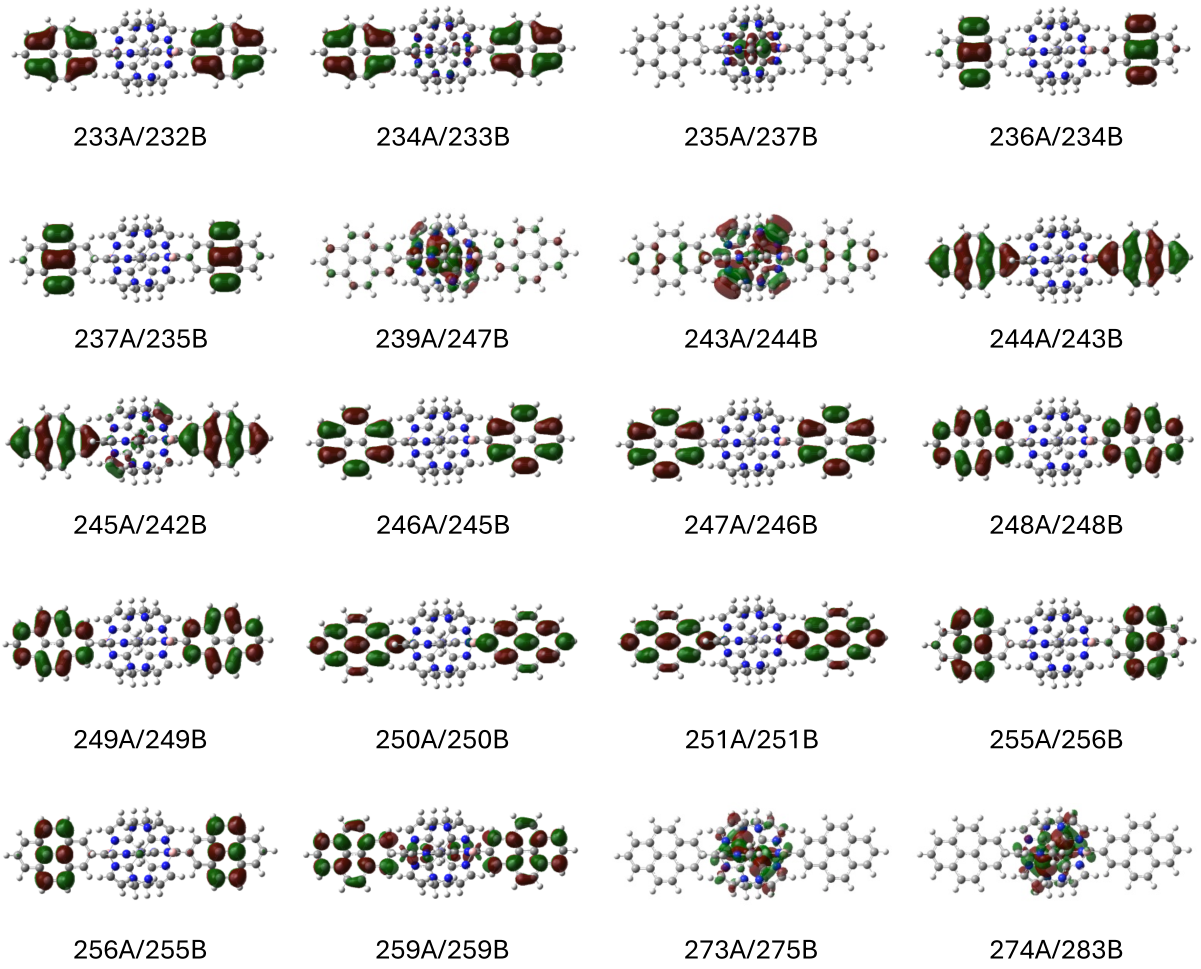

Supplementary Fig. 55. Isocontour representation (isovalue 0.02 a.u.) for the MOs mainly involved in the first 25 electronic excitations of compound **2**.

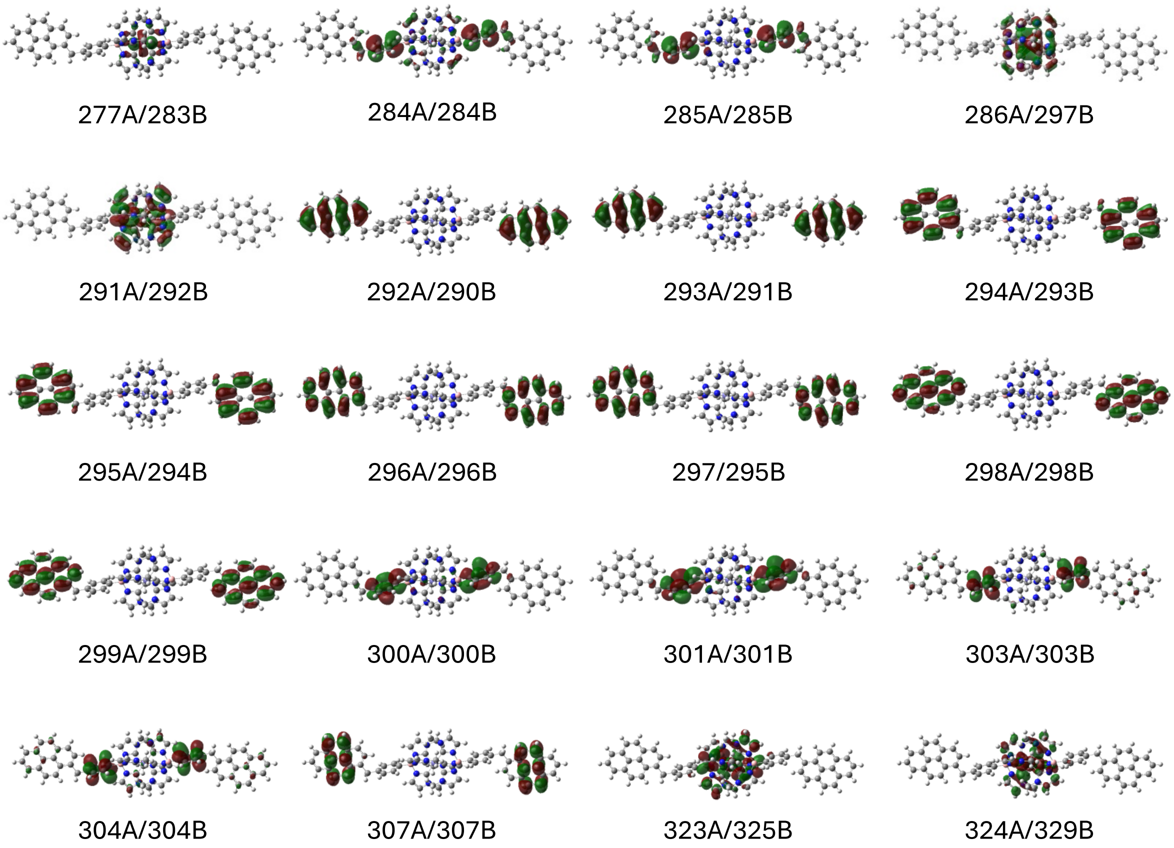

Supplementary Fig. 56. Isocontour representation (isovalue 0.02 a.u.) for the MOs mainly involved in the first 30 electronic excitations of compound **3**.

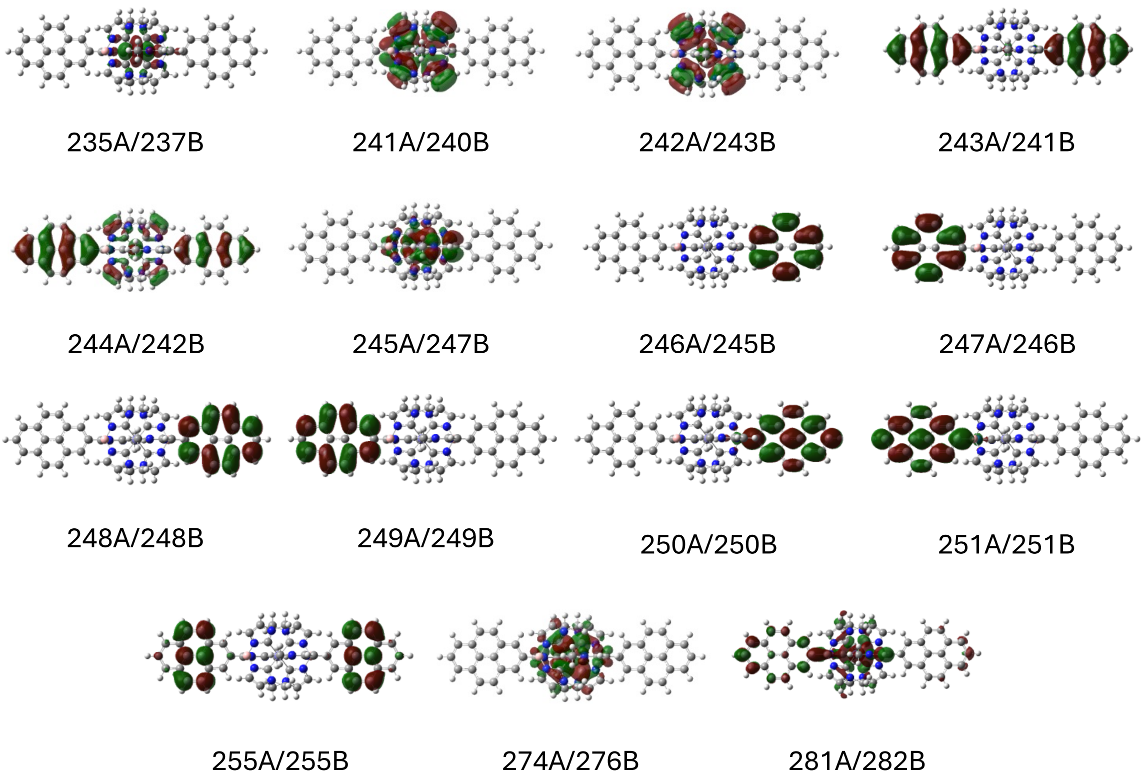

Supplementary Fig. 57: Isocontour representation (isovalue 0.02 a.u.) for the MOs mainly involved in the first 25 electronic excitations of the first optimised doublet of compound **2** (**2-D1**).

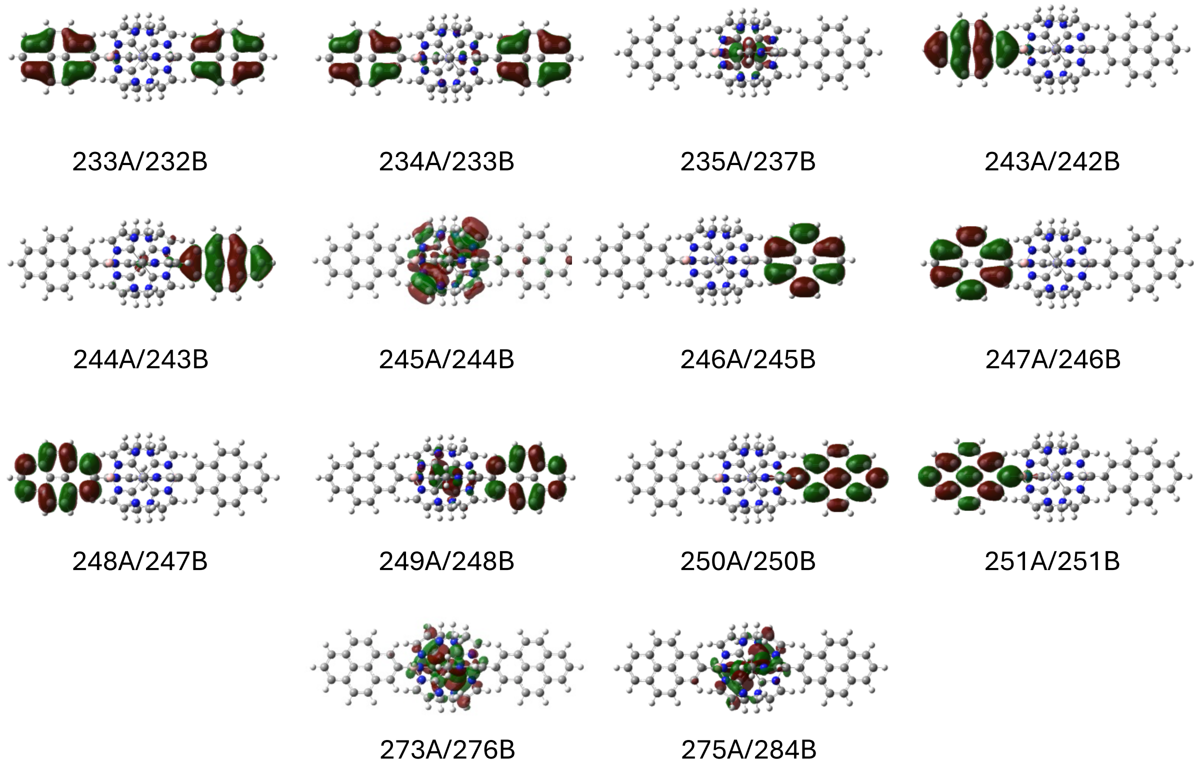

Supplementary Fig. 58. Isocontour representation (isovalue 0.02 a.u.) for the MOs mainly involved in the first 25 electronic excitations of the sixth optimised quartet of compound **2** (**2-Q6**).

Spin Density Isocontours

Spin density isocontour representations of all open-shell systems for both ligands and metal complexes are displayed in **Supplementary Figs. 59-61**. Relative energy with respect to their optimised ground state (GS) is depicted in eV.

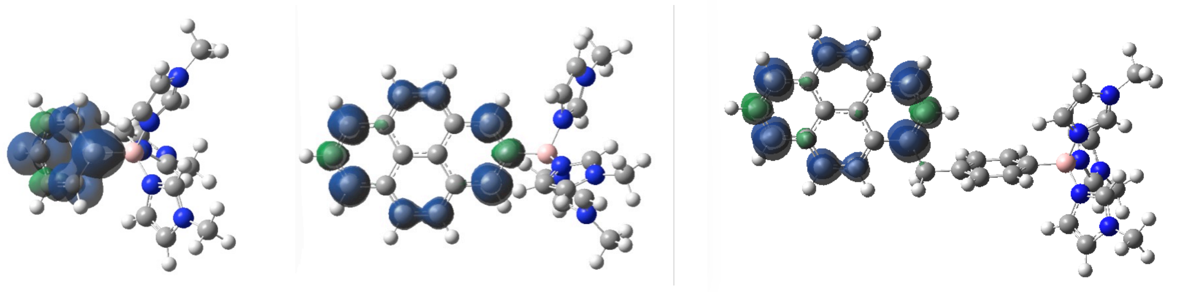

Phtmeimb-T0 (3.6) **A-T0** (2.2) **B-T0** (2.1)

Supplementary Fig. 59. Isocontour representation (isovalue 0.004) for the alpha (blue) and beta (green) spin density for all studied ligands. Relative energy difference respect to the GS optimised geometry is displayed in eV.

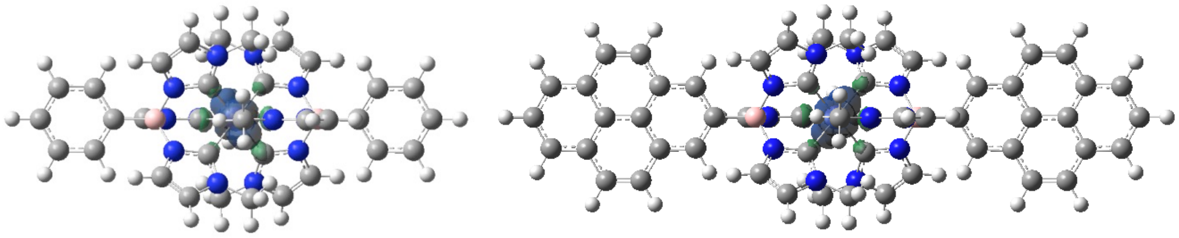

1 (0.0) **2** (0.0)

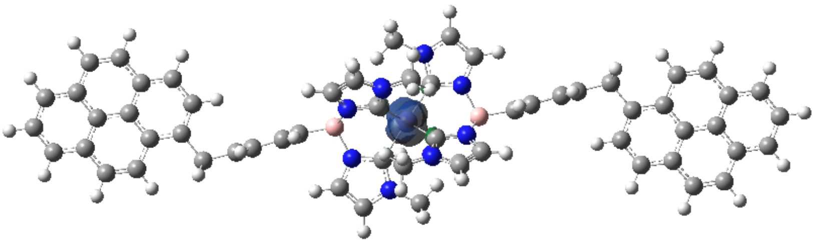

3 (0.0)

Supplementary Fig. 60. Isocontour representation (isovalue 0.004) for the alpha (blue) and beta (green) spin density for the ground state compounds **1-3**. Relative energy difference respect to the GS optimised geometry is displayed in eV.

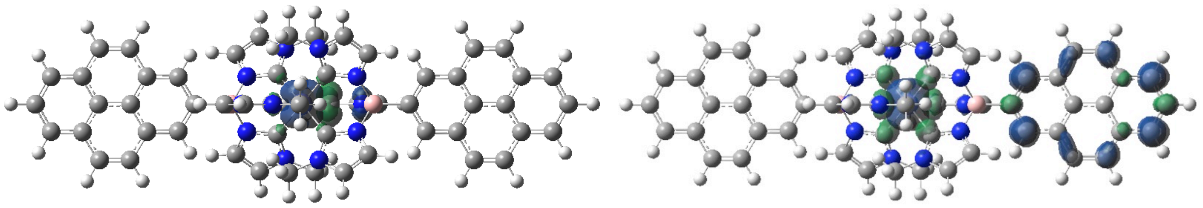

2-D1
(3.5)

2-Q6
(3.6)

Supplementary Fig. 61. Isocontour representation (isovalue 0.004) for the alpha (blue) and beta (green) spin density for the first doublet and sixth quartet of the compound **2**. Relative energy difference respect to the GS optimised geometry is displayed in eV.

Supplementary Transient Absorption Spectra

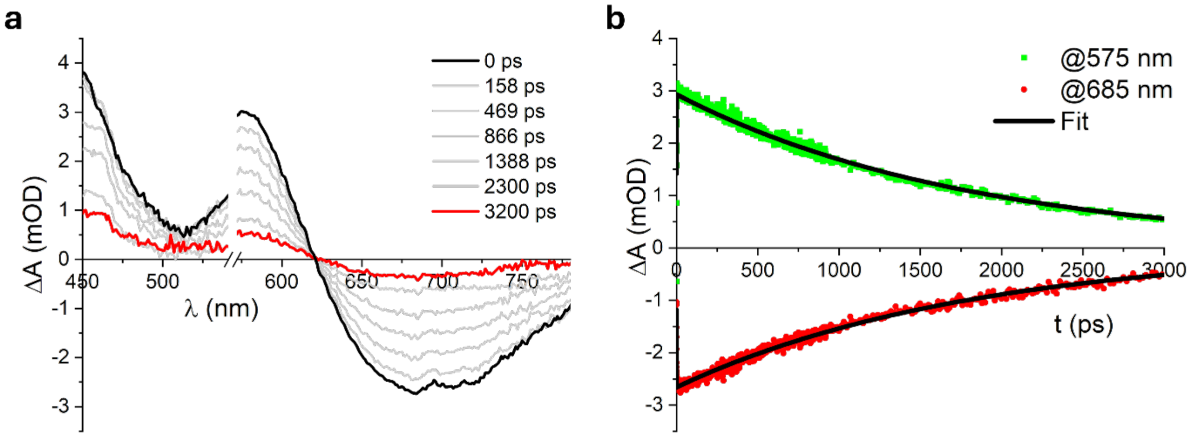

Supplementary Fig. 62. Panel a: Pump-probe transient absorption spectra (TAS) of **3** in deoxygenated acetonitrile. Panel b: Transient absorption kinetics of **3** in acetonitrile. Data are well fitted with a time constants of 1.80 ns.

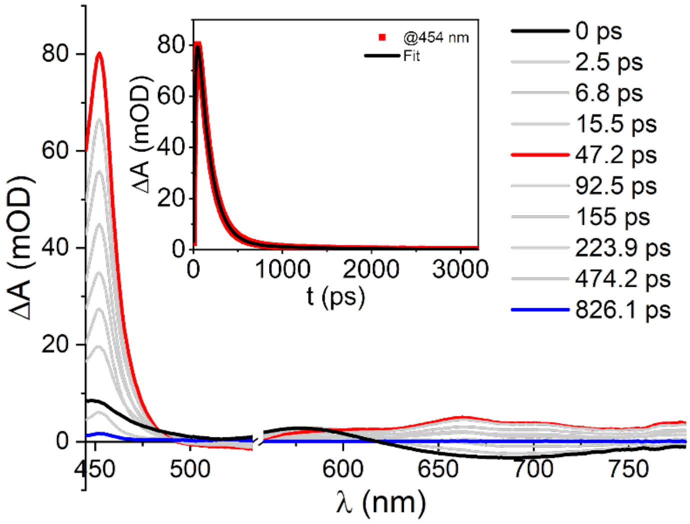

Supplementary Fig. 63. Pump-probe TAS of **2** in deoxygenated acetonitrile. Inset: Transient absorption kinetics of **2** in acetonitrile. The data are well fitted with time constants of 23 and 138 ps.

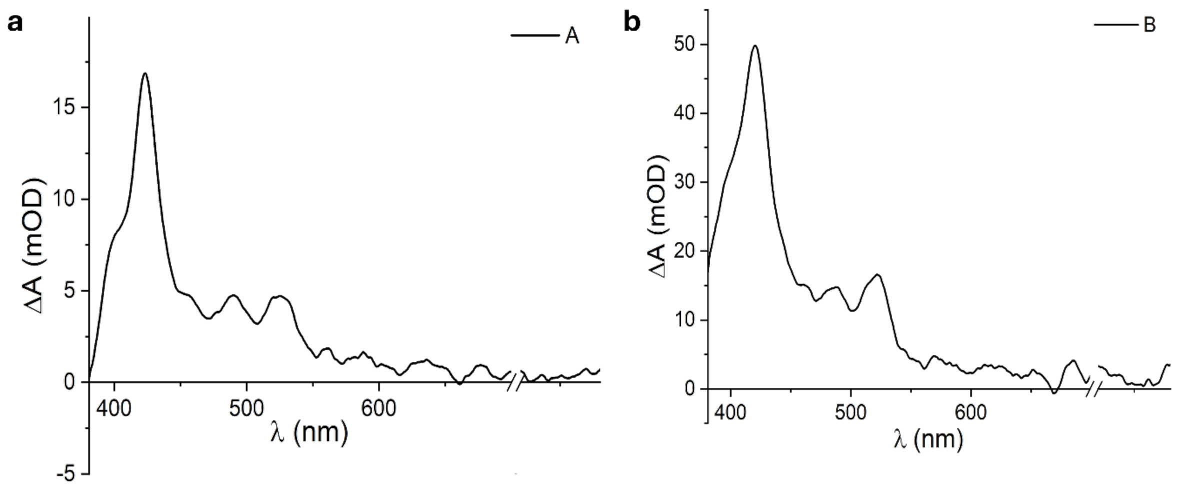

Supplementary Fig. 64. Nanosecond transient absorption spectra of ligands **A** (panel a) and **B** (panel b) in deoxygenated DCE, recorded after 200 ns from excitation at 355 nm. Iodomethane (10 %) was added to **A** and **B** solutions to enhance intersystem crossing. The spectra are considered as the *bonafide* transient absorption spectra of the pyrene triplet states in **2** and **3**, respectively.

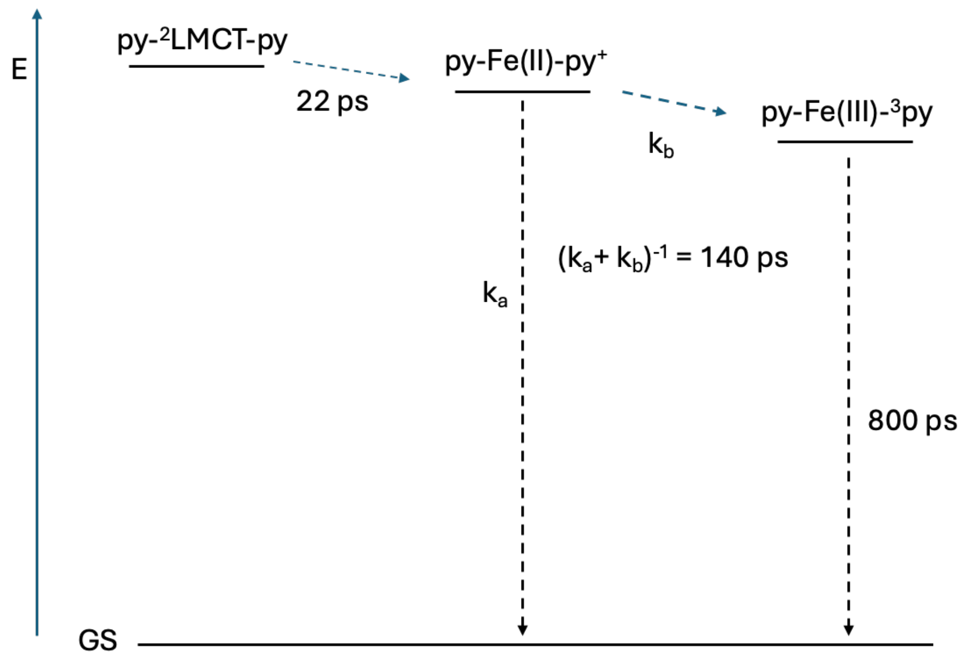

Supplementary Fig. 65. Representation of the excited state levels and decay processes occurring in **2** in AN solution, according to TAS experiments and global kinetic analysis. ^{2}LMCT is reported as py-^{2}LMCT-py to evidence the presence of the pyrene (py) groups. Details in the main text.

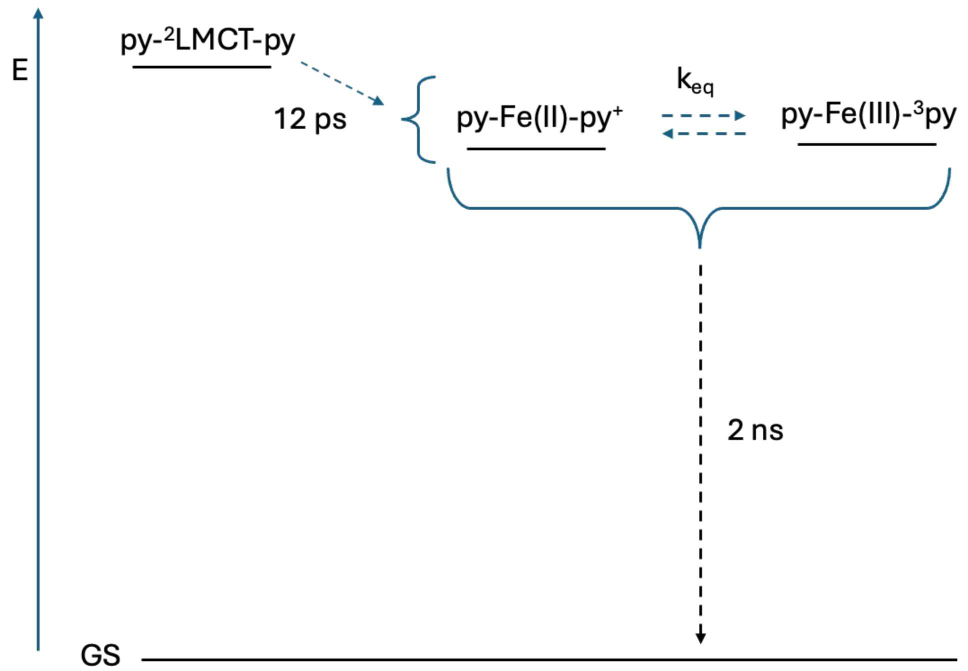

Supplementary Fig. 66. Representation of the excited state levels and decay processes occurring in **2** in DCE solution, according to TAS experiments and global kinetic analysis. ^{2}LMCT is reported as py-^{2}LMCT-py to evidence the presence of the pyrene (py) groups. Details in the main text.

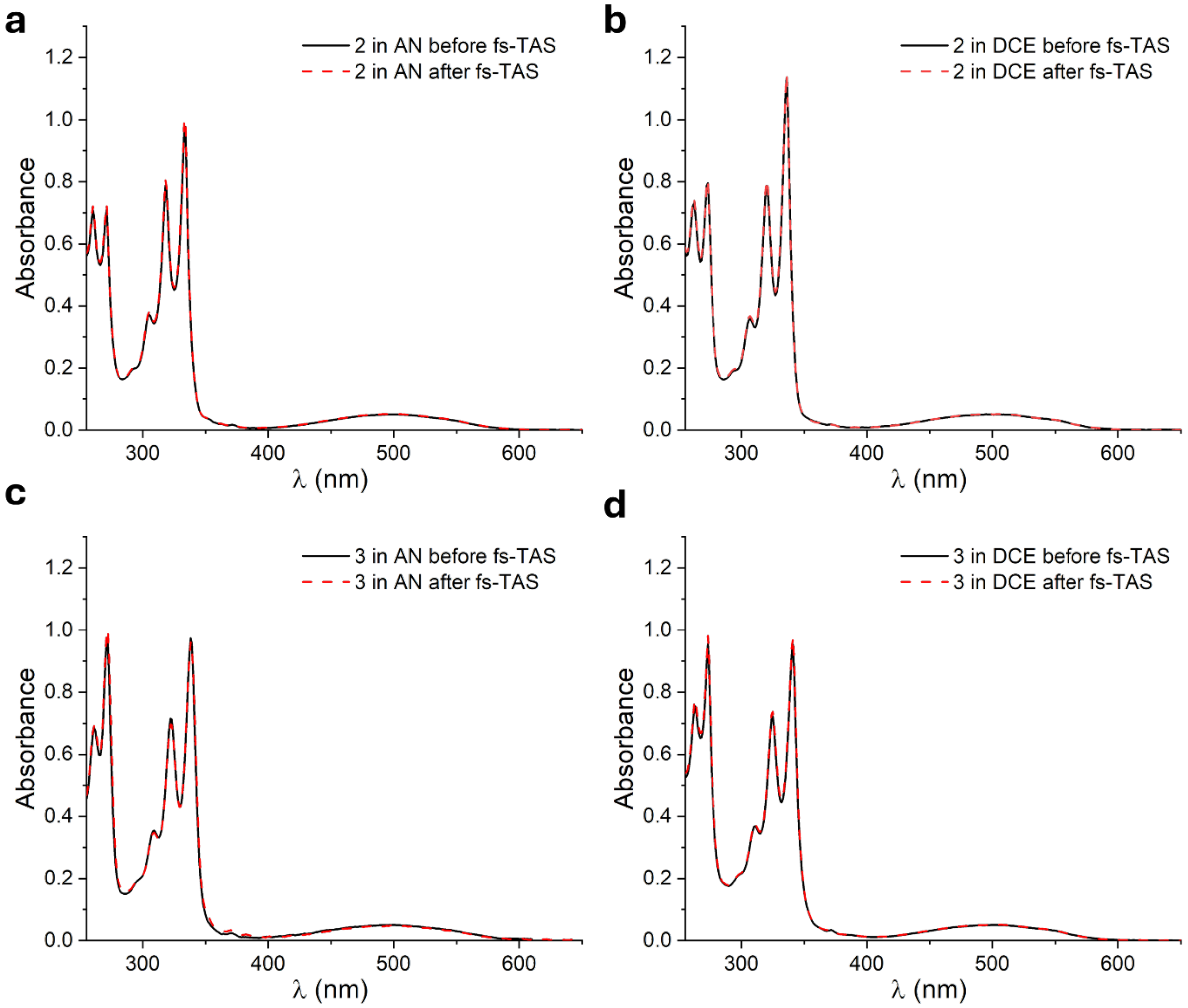

Supplementary Fig. 67. Absorption spectra of **2** and **3** in acetonitrile and dichloroethane before and after pump probe experiments (pump at 550 nm). The solutions are obtained by taking 30 μL of a non-irradiated concentrated solution and diluting it with 3 mL of the corresponding solvent in a 1 cm cuvette; the same was repeated using the irradiated 30 μL (formerly taken from the same concentrated solution). The comparison shows that the pump-probe experiments do not produce photodegradation.

Supplementary References

1. McClenaghan, N. D., Leydet, Y., Maubert, B., Indelli, M. T. & Campagna, S., *Coord. Chem. Rev.* **249**, 1336-1350 (2005)

2. Passalacqua, R., Loiseau, F., Campagna, S., Fang, Y.-Q. & Hanan, G. S., *Angew. Chem. Int. Ed.* **42**, 1608-1611 (2003)

3. Balzani, V. & Scandola, F. *Supramolecular Photochemistry*, Horwood, Chichester, 1991, Chapter 3.

4. Sutin, N. Theory of Electron Transfer Reactions: Insights and Hindsights, *Progr. Inorg. Chem.* **30**, 441 (1983). Doi:10.1002/9780470166314.CH9.

5. Frisch, M. J. et al. Gaussian 16 Rev. C.01. (2016).

6. Lange, A. W., Rohrdanz, M. A. & Herbert, J. M. Charge-Transfer Excited States in a π-Stacked Adenine Dimer, As Predicted Using Long-Range-Corrected Time-Dependent Density Functional Theory. *J. Phys. Chem. B* **112**, 6304–6308 (2008). Doi:10.1021/jp802058k.

7. McLean, A. D. & Chandler, G. S. Contracted Gaussian basis sets for molecular calculations. I. Second row atoms, Z=11–18. *J. Chem. Phys.* **72**, 5639–5648 (1980). Doi:10.1063/1.438980.

8. Krishnan, R., Binkley, J. S., Seeger, R. & Pople, J. A. Self-consistent molecular orbital methods. XX. A basis set for correlated wave functions. *J. Chem. Phys.* **72**, 650–654 (1980). Doi:10.1063/1.438955.

9. Hay, P. J. & Wadt, W. R. Ab initio effective core potentials for molecular calculations. Potentials for K to Au including the outermost core orbitals. *J. Chem. Phys.* **82**, 299–310 (1985). Doi:10.1063/1.448975.

10. Roy, L. E., Hay, P. J. & Martin, R. L. Revised Basis Sets for the LANL Effective Core Potentials. *J. Chem. Theory Comput.* **4**, 1029–1031 (2008). Doi:10.1021/ct8000409.

11. Miertuš, S., Scrocco, E. & Tomasi, J. Electrostatic interaction of a solute with a continuum. A direct utilizaion of AB initio molecular potentials for the prevision of solvent effects. *Chem Phys.* **55**, 117–129 (1981). Doi:10.1016/0301-0104(81)85090-2.

12. Cossi, M., Barone, V., Cammi, R. & Tomasi, J. Ab initio study of solvated molecules: a new implementation of the polarizable continuum model. *Chem. Phys. Lett.* **255**, 327–335 (1996). Doi:10.1016/0009-2614(96)00349-1.

13. Ernzerhof, M. & Scuseria, G. E., Assessment of the Perdew–Burke–Ernzerhof exchange-correlation functional. *J. Chem. Phys.* **110**, 5029–5036 (1999). Doi:10.1063/1.478401.

14. Adamo, C. & Barone, V. Toward reliable density functional methods without adjustable parameters: The PBE0 model. *J. Chem. Phys.* **110**, 6158–6170 (1999). Doi:10.1063/1.478522.
