# Supplementary material for: Fe(III) complexes with prolonged luminescence lifetimes via excited-state equilibration promoted by reversible intercomponent electron transfer
Source: Nat Commun. 2026 Apr 11;17:5090. doi: 10.1038/s41467-026-71767-4 (PMC13247219; doi:10.1038/s41467-026-71767-4)
Supplement: Supplementary file 2 — Description of Additional Supplementary Information [file 41467_2026_71767_MOESM2_ESM.pdf]

Description of Additional Supplementary Information

Supplementary Data 1

Cartesian coordinates of all optimized structures.

Supplementary Data 2

Supplementary Tables 4-11
