# Supplementary material for: Fe(III) complexes with prolonged luminescence lifetimes via excited-state equilibration promoted by reversible intercomponent electron transfer
Source: Nat Commun. 2026 Apr 11;17:5090. doi: 10.1038/s41467-026-71767-4 (PMC13247219; doi:10.1038/s41467-026-71767-4)
Supplement: Supplementary file 5 — Transparent Peer Review file [file 41467_2026_71767_MOESM5_ESM.pdf]

nature portfolio

Peer Review File

Fe(III) complexes with prolonged luminescence lifetimes via excited-state equilibration promoted by reversible intercomponent electron transfer

Corresponding Author: Professor Sebastiano Campagna

This file contains all reviewer reports in order by version, followed by all author rebuttals in order by version.

Version 0:

Reviewer comments:

Reviewer #1

(Remarks to the Author)
The manuscript describes new Fe complexes in which the lifetime of an excited state (for one of the compounds) reaches 6.5 ns at r.t. For an iron-based species, this is very long. The main interest here is in applying a strategy of prolonging the excited state lifetime via an equilibrium with a close-lying long-lived state. In this case, this is an equilibrium with a pyrene-based state. It is also interesting that the authors combine Fe photochemistry with this of boron-based dyes. The symmetry-breaking is very interesting and probably a first for Fe complexes in this context.
Those above are the key aspects of the novelty of the work.

The interest in excited state properties of iron complexes is very high, for the reasons of them being a potential replacement for the complexes of Ru(II) and other rare and expensive metals in photochemical applications.

The work will be of interest to a broad research community and is suitable for publication in Nature Comm, on the basis of the novelty and rigor of the work.

Several points that need to be addressed.

1. High-resolution MS is required.
2. A recurring issue with Fe complexes in general, as the authors of course appreciate, is the potential for changes in the oxidation state during the experiments. Is this possible to include excitation spectra (the authors do say that the emission is independent of the excitation wavelength, but including the spectra will be useful). Also, have there been any changes in the emission spectra/lifetimes after a long TA experiment?

3. The reasons for biexponential emission decay need to be explained in more detail (as a footnote or in the SI, if not in the main text). IRF needs to be overlapped with the emission kinetics.

4. The discussion centers on 6.5 ns lifetime for one of the compounds. It is part of a biexponential decay, which only occurs in dichloromethane. The emission decay trace in acetonitrile also has a (small) long-lived component it appears. If the 2 components are due to an equilibrium between a CT and a pyrene-based state, it will be important to include the emission data when direct excitation of pyrene is performed.

5. If symmetry-breaking takes place, it may also be reflected in the concentration dependence of the emission properties. Are the emission lifetimes affected by concentration?

6. The TA data as presented in the main text are difficult to follow. It may be simpler to show the spectra as they are shown in the SI, and add a 2D-picture of the spectra. The data fit is shown for selected kinetic traces in the TA. Global analysis of the data is required, to show much clearer the spectral and kinetic changes.

7. The symmetry-breaking proposed includes Fe(II) and Fe(IV). It could also in principle include pyrene species. The evidence for Fe(II) and Fe(IV) need to be clarified.

The manuscript presents interesting and important results. Its publication in Nature Communications is supported, subject to revisions suggested above.

Reviewer #2

(Remarks to the Author)
The authors present two pyrene-appended derivatives 2, 3 of the known iron(III) fluorophore 1. The aim was to exploit the so-called reservoir effect for iron(III) fluorophores to increase the excited state lifetime. The authors claim an excited state lifetime increase from 2.4 ns (1) to 6.5 ns (3) in CH2Cl2, which suggests an increase by a factor of 2.7.
However, for 2 and 3 the decays are biexponential and hence the value comparison is not fair. The authors should report the amplitudes for both lifetimes and calculate a weighted average value. This value might be compared with the lifetime of 1. With these values, the increase in lifetime is even less pronounced as proposed by the authors. In this reviewer's opinion, this rather weak effect of a known concept is not sufficiently new and ground-breaking for publication in Nature Communications. Similarly, the proposed symmetry-breaking charge-separation for 3 has been reported before for 1.

Furthermore, I have several major and minor issues that the authors should consider in any case:
1) As said above, the authors should report the amplitudes of the two lifetimes and calculate the weighted average lifetimes. With these values a fair comparison might be achieved.
2) In this reviewer's opinion, a different acceptor with somewhat lower triplet energy might have resulted in a more pronounced effect.
3) The authors measured the complexes 1-3 in different solvents at different concentrations. None of these experiments covers all aspects and hence the data are not reliably comparable as the authors mention themselves ("… so direct comparison between luminescence and pump-probe experiments could not be straightforward."). A full concentration dependent study might better substantiate the assignments of the two lifetime components (aggregation, static quenching). At the present stage, the assignments are not entirely convincing.
4) The TA experiments are somewhat out of context as different concentrations were used in emission and TA experiments resulting in variable aggregation effects.
5) The authors should mention that the emission from iron(III) is fluorescence (in contrast to the phosphorescence of typical triplet emitters). Hence, doublet-triplet energy transfer (DTET) excites the appended organic chromophores. DTET to organic chromophores has been observed before for chromium(III) and recently even for iron(III) doublet excited states (e.g. ACIE 2022, e202202238; JACS 2024, 11299).
6) The emission spectra in Fig. S18 belong to the pro-ligands, i.e. the imidazolium salts, and not to the NHC ligands. Hence, the carbenes and imidazoliums should possess very different properties.
7) Given that the emission of 1-3 is spin-allowed fluorescence, this reviewer is does not understand why the authors measured the phosphorescence of the pro-ligands A and B.
8) Chromophore-appended 3d metal complexes have been reported before: e.g. JACS 2021, 15800 (ref. 14); Inorg. Chem. 2020, 14746. This should be mentioned.
9) The characterization of the materials and the proof of purity is insufficient. Mass spectrometric data, full absorption spectroscopic data and elemental analyses are missing.
10) "Emission is slightly blue shifted on passing from CH3CN to CH2Cl2 solution for all the compounds, as usually expected for CT emissions".
Solvatochromism is typical for CT transitions. But it is not a priori clear, whether it is negative or positive solvatochromism. Literature references should be given.
11) "Although the different spin multiplicity of the two states could slow down the intercomponent energy transfer process, …" and "in spite of the different spin multiplicity of the involved excited states": In this reviewers opinion, the total spin has to be considered and does probably not change due to magnetic interactions: 2GS -> 2LMCT-> 2(2GS antiferromagnetically coupled with 3(pi-pi*(pyrene)). The DTET process can be easily described by a Dexter double electron exchange keeping the overall spin (see point 5).
12) The entire discussion and tentative assignments and TA interpretation might strongly profit form quantum chemical calculations, in particular TDDFT.
13) The TA spectra should be simulated from the spectroelectrochemical measurements by depicting the respective difference spectra.
14) "Therefore, in the transient absorption spectrum of 3 recorded after 3 ps from pulse (Figs. 4b and S23), the band at about 500 nm can be due to triplet-triplet pyrene-based transitions, the band at about 560 nm can receive main contribution from the 2LMCT state, and the intense band at about 460 nm can receive contributions from transitions involving both pyrene triplet and LMCT states. This tends to suggest that the pyrene excimer state deactivates to the lower-lying pyrene-based triplet and LMCT states."
This section is really hard to understand. It is not clear to this reviewer, what the authors want to explain here.
15) The formal positive charges for 1-3 (Fig. 1) are wrong.
16) Ref. 35 "and ref. cited therein" If these references are of significant importance, then they should be cited individually.
17) Ru(bpy)32+: brackets are missing => [Ru(bpy)3]2+
18) phtmeimb = {phenyl[tris(3-methylimidazol-1-ylidene)]borate} anion -> minus missing phtmeimb-
19) In the conclusion, the authors estimate (on the basis of a 7 ns lifetime, 100% !) a Stern-Volmer quenching rate increase. However, this has not been shown experimentally and correspond quenching experiments need to be conducted to prove such an effect.

Reviewer #3

(Remarks to the Author)
The manuscript by Campagna and co-workers reports long-lived luminescence from iron(III) complexes with pyrene moieties in fluid solution at room temperature. Various pyrene-bound luminescent metal complexes exhibiting long-lived luminescence have been reported in the past 30 years. For example, the emission lifetime of a ruthenium(II) complex with three pyrene moieties is as long as 9.0 μs as reported by Tyson and Castellano. In these systems, the pyrene moiety acts as an energy reservoir, and the emitting excited state is generated via the intramolecular energy transfer from the long-lived triplet excited state of the pyrene moiety. The authors introduced the pyrene moieties into a luminescent iron(III) N-heterocarbene complex and succeeded in obtaining long-lived luminescence up to 6.5 ns at room temperature. The luminescence lifetime is significantly long in comparison to other iron(III) complex. The excited states with a >5-ns lifetime is utilizable as a photosensitizer in a variety of solar–energy conversion systems and, therefore, this work may open the door to use earth-abundant iron(III) complexes. However, the lifetime being 6.5 ns for complex 3 in dichloromethane is not necessarily surprising and very long since that of the reported complex without the pyrene moiety (complex 1) is 2.4 ns under the identical conditions. Furthermore, the experimental evidence for the excited-state equilibration is not sufficient. Transient absorption data for both complexes 2 and 3, unfortunately, exhibited another photophysical processes due to aggregation arising from high sample concentrations. Temperature-dependent steady-state and time-resolved luminescence measurements were also not performed. Thus, the reviewer suggests that the manuscript is not suitable for a publication in Nature Communications at this stage.

Version 1:

Reviewer comments:

Reviewer #1

(Remarks to the Author)
The revised manuscript is of excellent quality and should be published as is.

The authors undertook a remarkable amount of extra work, and addressed every comment that was made in the original review. It is also notable that a "competing" work appeared in JACS in Nov 2025, which references the work currently presented as it was placed as an open source.

This is an excellent extension of the concept of a "reservoir state" which led to a remarkably long lifetime for Fe complexes.

It will be very beneficial to the community (as the above JACS paper already demonstrates...).

Reviewer #3

(Remarks to the Author)
The authors performed additional experiments such as nanosecond transient absorption and, as results, detailed excited-state dynamics, including nature of the biexponential emission decays, of iron(III) complexes with pyrene moieties were revealed. The long-lived excited state by utilizing an equilibrium between the emitting and charge-separated states is fascinating strategy toward future design of the luminescent complexes of redox-active iron and enhances applicability of earth-abundant metal complexes. The present manuscript is now publishable in Nature Communications. The reviewer concerns the presence (or contribution) of the spin quartet state(s) in the excited-state dynamics of the complexes. The theoretical calculations added in the revised manuscript suggest that there are lower-lying quartet states than the doublet excited states. As several JACS papers have recently reported that charge-separation process plays an important role in the spin-state modulation in organic compounds bearing a spin-doublet substituent(s), charge separation in the present system being spin-doublet iron(III) with aromatic hydrocarbon might possess significant contribution to the excited-state dynamics of the complexes. Including the descriptions of the presence and contribution of the quartet states is recommended (additional experiments are not required).

Open Access This Peer Review File is licensed under a Creative Commons Attribution 4.0 International License, which permits use, sharing, adaptation, distribution and reproduction in any medium or format, as long as you give appropriate credit to the original author(s) and the source, provide a link to the Creative Commons license, and indicate if changes were made.
In cases where reviewers are anonymous, credit should be given to 'Anonymous Referee' and the source.
The images or other third party material in this Peer Review File are included in the article's Creative Commons license, unless indicated otherwise in a credit line to the material. If material is not included in the article's Creative Commons license and your intended use is not permitted by statutory regulation or exceeds the permitted use, you will need to obtain permission directly from the copyright holder.
To view a copy of this license, visit https://creativecommons.org/licenses/by/4.0/

REVIEWER COMMENTS – point-by-point answers

Reviewer #1:

The manuscript describes new Fe complexes in which the lifetime of an excited state (for one of the compounds) reaches 6.5 ns at r.t. For an iron-based species, this is very long. The main interest here is in applying a strategy of prolonging the excited state lifetime via an equilibrium with a close-lying long-lived state. In this case, this is an equilibrium with a pyrene-based state. It is also interesting that the authors combine Fe photochemistry with this of boron-based dyes. The symmetry-breaking is very interesting and probably a first for Fe complexes in this context.

Those above are the key aspects of the novelty of the work.

The interest in excited state properties of iron complexes is very high, for the reasons of them being a potential replacement for the complexes of Ru(II) and other rare and expensive metals in photochemical applications.

The work will be of interest to a broad research community and is suitable for publication in Nature Comm, on the basis of the novelty and rigor of the work.

Several points that need to be addressed.

1. High-resolution MS is required.

Mass spectra, obtained by a Time of Flight – Secondary Ion Mass Spectrometer (TOF-SIMS) have been added to the Supplementary Information (Figs. S17-S24). A brief paragraph on the experimental equipment used is also given in the Materials and Method section of the main text (with more extended details in the Supplementary Information). Experts in TOF-SIMS are added to the coauthor list.

2. A recurring issue with Fe complexes in general, as the authors of course appreciate, is the potential for changes in the oxidation state during the experiments. Is this possible to include excitation spectra (the authors do say that the emission is independent of the excitation wavelength, but including the spectra will be useful). Also, have there been any changes in the emission spectra/lifetimes after a long TA experiment?

*Excitation spectra are now added to the Supplementary Information (**Fig. S27**). As far as the stability of the compounds is concerned, we do not note any changes in the emission spectra and lifetimes after the TA experiments (this fully agrees with the photostability of compound **1**, already*

*reported by other groups). Emission spectra after the TA experiments are also shown in the Supplementary Information data, to support this statement (**Fig. S57**).*

3. The reasons for biexponential emission decay need to be explained in more detail (as a footnote or in the SI, if not in the main text). IRF needs to be overlapped with the emission kinetics.

*We added a brief discussion to the main text (**page 2, lines 19-33)** and a short chapter in the SI ("Excited-state equilibration and biexponential emission decay", within the "Supplementary Discussion – Part 1" section) to explain in more detail the reasons for biexponential emission decay in the presence of an excited-state equilibration. IRF has been overlapped with the emission kinetics (see **Fig. 3**).*

4. The discussion centers on 6.5 ns lifetime for one of the compounds. It is part of a biexponential decay, which only occurs in dichloromethane. The emission decay trace in acetonitrile also has a (small) long-lived component it appears. If the 2 components are due to an equilibrium between a CT and a pyrene-based state, it will be important to include the emission data when direct excitation of pyrene is performed.

*The luminescence spectra and lifetimes of **2** and **3** when excited in the 280-350 nm region (that is, directly exciting the pyrene moieties) are identical to the data obtained by excitation in the LMCT state. In principle, the singlet states of pyrene (the triplet state is practically not absorbing, so it cannot be excited directly: in any case, its -very low- absorption would be obscured by the much stronger LMCT absorbance) is expected to deactivate with a relatively good efficiency to the low-lying doublet LMCT state, from which equilibration with charge-separated state occurs. However, a decrease in quantum yield occurs for excitation higher in energy than 350 nm, where pyrene absorption dominates, particularly for **3** in acetonitrile. Excitation spectra vs absorption spectra are shown in **Fig. S27**, and clearly show that part of the light directly absorbed by the pyrene units does not contribute to LMCT emission. A note has also been added to the main text to report this information (**ref 47**). This indicates that the pyrene singlet state partly decays to the ground state by a different, radiationless route. This route could be oxidative electron transfer from the singlet state of pyrene to Fe(III) subunit, with formation of the charge separated state (from which the "delayed" emission originates), and this would suppress a percentage of prompt emission. Direct decay of a anthracene-type singlet state to a Fe(III) subunit in a molecular dyad has been reported in a recently published paper (Glaser et al., ACS Cent. Sci.* https://doi.org/10.1021/acscentsci.5c01040). *Indeed, the percentage of "prompt" and "delayed" emission changes in favor of the delayed emission when excitation wavelength is moved from 456 to 296 nm (e.g., for emission of **3** in dichloromethane, when*

*excitation wavelength is 456 nm the percentages of prompt and delayed emission are 79% and 21%, respectively (see **Table 1**), while when excitation wavelength is 296 nm the percentages of prompt and delayed emission become 61% and 39%, respectively). However, this aspect is not studied here in detail, since we believe the paper contains already a lot of information, and to add some more issues would lead to lose focus on the main objective of the work, that is to introduce the possibility of increasing the emission lifetime of Fe(III) compounds by reversible intramolecular electron transfer.*

5. If symmetry-breaking takes place, it may also be reflected in the concentration dependence of the emission properties. Are the emission lifetimes affected by concentration?

*We performed concentration-dependent luminescence experiments. The effect of concentration on the emission quantum yields of **2** and **3** are reported in **Figs. S32** and **S33.** From these experiments, now it appears clearly that in **2** aggregation occurs already in dilute conditions (around 1 x 10^{-5} M), with the emission only derived from the non-aggregated species, since luminescence lifetimes do not change with concentration, opposite to what happens for emission quantum yields (i.e., the quenching of emission is due to static quenching). We corrected our former statement, showing that pyrene-to-*Fe(III) electron transfer, and not symmetry-breaking, occurs in **2** (as well as in **3**).*

6. The TA data as presented in the main text are difficult to follow. It may be simpler to show the spectra as they are shown in the SI and add a 2D-picture of the spectra. The data fit is shown for selected kinetic traces in the TA. Global analysis of the data is required, to show much clearer the spectral and kinetic changes.

*We agree with the referee. Now the TA spectra shown in the main text are of the same kind formerly reported in SI. Moreover, more TA experiments are now showed (also including flash photolysis ns experiments). Global analysis has also been performed, and indeed some features that were difficult to disentangle on single wavelength kinetics have been revealed, in particular the involvement of pyrene triplet states as a contributed decay route for the pyrene^{+}-Fe(II) charge separated state, which is the excited state involved in the equilibration responsible for the prolonged, long-lived emission component. The global kinetic analysis results are discussed at **Page** 7, and illustrated in the new **Figs. 6** and **8**. The schematic representation of excited state levels and processes shown in **Fig.** 7 (as well as in **Fig. S55** and **S56**) also relies on global kinetic analysis information.*

7. The symmetry-breaking proposed includes Fe(II) and Fe(IV). It could also in principle include pyrene species. The evidence for Fe(II) and Fe(IV) needs to be clarified.

We made many more transient absorption spectroscopy experiments, also using flash photolysis ns apparatus that allowed us to explore the UV region of the spectrum, where Fe(II) is expected to be seen. Moreover, we used a different setput for the pump-probe fs experiments, that allowed to obtain much better resolved spectra. These new experiments made clear that the charge-separated pyrene^{+}-Fe(II) species is formed from the initially prepared ^{2}LMCT state, and not symmetry-breaking product. The fact that symmetry-breaking for the model species **1** *was already reported, although in quite different experimental conditions, whereas excited-state equilibration involving charge-separated states was totally unknown for earth-abundant metal complexes (and very rarely reported for transition metal complexes in general), was the reason for our initial misunderstanding. We thank the reviewer to push us to make more experiments that allowed us to correct our initial statement and to reveal the unexpected, although thermodynamically allowed, behavior.*

The manuscript presents interesting and important results. Its publication in Nature Communications is supported, subject to revisions suggested above.

Reviewer #2:

The authors present two pyrene-appended derivatives 2, 3 of the known iron(III) fluorophore 1. The aim was to exploit the so-called reservoir effect for iron(III) fluorophores to increase the excited state lifetime. The authors claim an excited state lifetime increase from 2.4 ns (1) to 6.5 ns (3) in CH2Cl2, which suggests an increase by a factor of 2.7.

However, for 2 and 3 the decays are biexponential and hence the value comparison is not fair. The authors should report the amplitudes for both lifetimes and calculate a weighted average value. This value might be compared with the lifetime of 1. With these values, the increase in lifetime is even less pronounced as proposed by the authors. In this reviewer's opinion, this rather weak effect of a known concept is not sufficiently new and ground-breaking for publication in Nature Communications. Similarly, the proposed symmetry-breaking charge-separation for 3 has been reported before for 1.

One of the main points of the paper is the application of the excited-state equilibration approach to prolong lifetime of Fe(III) compounds. Despite the relatively small – however significative, as pointed out by the other reviewers – effect, our data clearly indicate that the approach is valid and can be successfully used to this scope, a result that was unprecedented – at the time of

*the initial submission - for early-transition metal complexes (indeed, during the revision of the present paper, a work has been published, see Wellauer at al., J. Am. Chem. Soc. 2025, 147, 8760-8768 (now **ref. 43**), in which excited-state equilibration involving organic triplet states is used to prolong the lifetime of Fe(III) compounds). Former attempts in this direction had previously failed (see Inorg. Chem. 2020, 14746, **ref. 41**).*

*The weighted average value of lifetimes is shorter than the lifetime of the equilibrated state since the prompt luminescence lifetime is obviously reduced. However, we believe that the weighted average value is less significant in the present case, where the aim is to demonstrate that extension of luminescence lifetime can be obtained via excited state equilibration. To eliminate misunderstanding, we have now clarified the meaning of "prolongation" we used here, which is not related to the weighted average value (see **ref. 50**).*

Furthermore, I have several major and minor issues that the authors should consider in any case:
1) As said above, the authors should report the amplitudes of the two lifetimes and calculate the weighted average lifetimes. With these values a fair comparison might be achieved.

*Relative amplitudes of lifetimes are now inserted in **Table 1**, but – as pointed out above – these data do not affect the experimental demonstration that excited-state equilibration is an effective manner to extend Fe(III) luminescence lifetime. Moreover, it is now shown that such an effect is obtained by the unusual involvement of a charge-separated state and not from the triplet state of pyrene (or similar aromatics), the "classical" way in which excited-state equilibration is obtained in transition metal complexes.*

2) In this reviewer's opinion, a different acceptor with somewhat lower triplet energy might have resulted in a more pronounced effect.

*We totally agree with referee. Indeed, this was already mentioned in the Conclusions section of the originally submitted paper (The Conclusions section reported: "*A careful design of coupled earth-abundant metal complexes and suitable organic chromophores – for example, increasing the energy of the metal-based CT excited state and lowering that of the organic-based triplet states, therefore increasing the percentage of the long-lived organic chromophore-based triplet state in the equilibrated state – could lead to a further increase in luminescence lifetimes so boosting the potential of such sustainable metal complexes for photochemical applications"*).*

However, the new results obtained by extensive transient absorption spectroscopy uncovered that electron transfer, and not energy transfer, promotes excited-state equilibration. Even in this case, the key to optimize the long-lived emission due to the equilibrated state over the "prompt" emission would be to increase the driving force for electron transfer quenching. This anyway leads to face with

the direct charge recombination to the ground state from the charge-separated state, and the balance between the various rate constants requires extreme care. This point is explicitly reported in the note contained in ***ref. 50****.*

3) The authors measured the complexes 1-3 in different solvents at different concentrations. None of these experiments covers all aspects and hence the data are not reliably comparable as the authors mention themselves ("… so direct comparison between luminescence and pump-probe experiments could not be straightforward."). A full concentration dependent study might better substantiate the assignments of the two lifetime components (aggregation, static quenching). At the present stage, the assignments are not entirely convincing.

The effect of concentration on the luminescence quantum yields of ***2*** *and* ***3*** *are now discussed in some more details at* ***page 5, lines 11-20****, and shown in* ***Figs. S32*** *and* ***S33****. This section also reports the information that luminescence spectra and lifetimes are not affected by concentration changes (these experiments were already made, but we believed they were not so essential to be reported, for reason of space and conciseness). These results (suggesting static quenching) further supports the aggregation occurring in* ***2****, as already discussed (see also answer to referee 1, point 5), with the aggregation form being not emissive. In the concentration range we used for the luminescence experiments there is no effect for the emission properties of* ***3****, which does not aggregate (until in the explored concentration range). As far as the two lifetimes are concerned, they are nothing to do with aggregation, but belong to the "isolated" species, as now better exemplified by a brief discussion added to the main text* ***(page 2, lines 19-33)*** *and a short chapter in the SI ("Excited-state equilibration and biexponential emission decay", within the "Supplementary Discussion – Part 1" section), see also the answer to referee 1, point 3.*

4) The TA experiments are somewhat out of context as different concentrations were used in emission and TA experiments resulting in variable aggregation effects.

The TA experiments are essential to characterize the processes governing the formation of the excited state equilibration in ***3****. They confirm the excited-state equilibration, since the decay of the delayed LMCT emission (the longer-lived component of emission) is in fair agreement with the decay of the charge-separated state evidenced by TA experiments of* ***3*** *in apolar solvents, whereas the formation of the charge-separated state is in good agreement with the "prompt" emission lifetime (the shorter-lived emission component). TA experiments indicate qualitatively the same behavior for the excited state decay of* ***2****, although a similar, direct parallelism between charge separation formation and decay rates obtained by TA with prompt and delayed (prolonged) emission is not*

possible, since TA experiments essentially involve the non-emissive aggregate species (by far the dominant species in solution), while the emission comes from the "isolated" species. Comparison between luminescence and TA data suggests that the corresponding processes are faster in aggregate species than in isolated, monomeric species, not an unexpected behavior when dependence of photoinduced electron transfer on aggregation is considered (see ***Page 6, lines 26-34, refs 58,59)****.*

5) The authors should mention that the emission from iron(III) is fluorescence (in contrast to the phosphorescence of typical triplet emitters). Hence, doublet-triplet energy transfer (DTET) excites the appended organic chromophores. DTET to organic chromophores has been observed before for chromium(III) and recently even for iron(III) doublet excited states (e.g. ACIE 2022, e202202238; JACS 2024, 11299).

The (formally) fluorescent nature of the LMCT emission is now clearly written (see ***page 2, line 4 from the bottom)****; we thought it was obvious, but we agree with the referee that it is better to state it.*

As mentioned above, extensive transient absorption spectroscopy experiments now clearly show that electron transfer and not energy transfer drives the excited-state equilibration formation, although energy transfer would be possible, as now also supported by the calculation section added ***(from page 4, last two lines, to page 5, line 10****; more computational data are also reported in the* ***Supplementary Discussion: Part 2****, also including* ***Figs. S36-S51*** *and* ***Tables S4-S11)*** *and indeed the pyrene triplet state has a role in deactivating the charge-separated state (see global kinetic analysis,* ***page*** *7 and* ***Fig.*** *7). The information that DTET was already reported in bimolecular systems involving Cr(III) and anthracene derivatives and Fe(III) and anthracene derivatives paper) has been added* ***(page 5, lines 9-11, refs. 51,52)****. In the original manuscript, we explicitly referred to multichomophoric species, for this reason the bimolecular energy transfer examples were not quoted. However, we agree that this information is useful. After the submission of the original manuscript, a paper has been published in which DTET in multichromophoric species involving Fe(III) is reported. This latter paper is here also quoted (****ref. 65****). That paper also quotes our originally submitted manuscript as reported in Research Square.*

6) The emission spectra in Fig. S18 belong to the pro-ligands, i.e. the imidazolium salts, and not to the NHC ligands. Hence, the carbenes and imidazoliums should possess very different properties.

It is not easy to study the emission spectra of the NHC ligands: the imidazolium salts are the best models we can think about. Similar approximation (e.g., to study the 2-phenylpyridine, ppyH, instead of the phenylpyridine anion, ppy) was successfully largely used in the past to get information

on the photophysical properties of phenylpyridine anions and similar ligands, when coordinated to transition metal complexes like Ir(III), Rh(III) or Pt(II) (for a review reporting some data and discussion, see: M. Maestri, V. Balzani, C. Deuschel-Cornioley, A. von Zelewsky, Photochemistry and Luminescence of Cyclometallated Complexes, Advances in Photochemistry, 1992, pp. 1-68). Moreover, the emission spectra at 77 K of A and B are useful to gain information on the energy of the appended pyrene-based triplet states, and these should not be significantly affected by difference between the NHC ligands and their imidazolium salts. Please note that in the revised version, Fig. S18 is now Fig. S26.

7) Given that the emission of 1-3 is spin-allowed fluorescence, this reviewer is does not understand why the authors measured the phosphorescence of the pro-ligands A and B.

*The excited states involved in the equilibration process if governed by energy transfer would be the closely lying ^{2}LMCT and pyrene-based $^{3}\pi-\pi*$ levels. To have information on these pyrene-based $^{3}\pi-\pi*$ states in **2** and **3**, including their energies, the phosphorescence spectra of A and B are needed.*

8) Chromophore-appended 3d metal complexes have been reported before: e.g. JACS 2021, 15800 (ref. 14); Inorg. Chem. 2020, 14746. This should be mentioned.

*We thank the referee for having attracted our attention to these papers. Indeed the IC paper reports a previous attempt to take advantage of the excited-state equilibration approach for Fe(II) – not Fe(III) – compounds, but unsuccessful. In the IC paper, excited-state equilibration between the ^{3}MLCT state of the iron(II) complex and the $^{3}\pi-\pi*$ state of the appended anthracene failed to occur, probably since the lifetime of the MLCT state was too short (ca 13 ps) and energy transfer to anthracene could not compete. This is now mentioned and briefly discussed in the paper (**note 38, refs. 38,41)**. As far as the JACS 2021 paper is concerned, the pyrene subunits there reported have the role of increasing the delocalization of the excited electron in the photoactive MLCT state (the "electron delocalization" effect originally reported, among others, by T. J. Meyer - Inorg. Chem. 2001, 40, 4508 - and investigated by several groups, including some of us several years ago – see for example J. Am. Chem. Soc., **2002**, 124, 791 or Chem. Commun., **2006**, 1301), which increase lifetimes by reducing the Franck-Condon factor for radiationless decay. Reference to this behavior is now also reported in **note 38**.*

9) The characterization of the materials and the proof of purity is insufficient. Mass spectrometric data, full absorption spectroscopic data and elemental analyses are missing.

High-resolution Mass spectra obtained by ToF-SIMS, elemental analysis and full absorption spectroscopic data have been added to the Supplementary Material. Mass spectra are shown in ***Figs. S17-S24****, and the equipment used is reported in the Materials and Methods section of the main text. Elemental analysis and full absorption data are added to the synthesis and characterization section.*

10) "Emission is slightly blue shifted on passing from CH3CN to CH2Cl2 solution for all the compounds, as usually expected for CT emissions".
Solvatochromism is typical for CT transitions. But it is not a priori clear, whether it is negative or positive solvatochromism. Literature references should be given.

When not specified, "solvatochromism" is intended as "positive" solvatochromism, experimentally translating into a red shift of the transition (and absorption/emission band) on passing from less polar to more polar solvents, indicative that the polarity of the excited state is larger than the ground state. "Negative" solvatochromism" is anyway found also for CT states, like is the case for Re(bpy)(CO)$_3$Cl and similar compounds. When negative solvatochromism occurs, this is clearly mentioned, but it is not strictly needed for positive solvatochromism, since the word solvatochromism implies positive solvatochromism. However, the sentence "positive solvatochromism" is now reported in the caption of ***Fig. S25****, to eliminate any doubt. This is quite established a behavior, we do not believe literature references are really needed here (any good photochemistry book reports this effect).*

11) "Although the different spin multiplicity of the two states could slow down the intercomponent energy transfer process, …" and "in spite of the different spin multiplicity of the involved excited states": In this reviewers opinion, the total spin has to be considered and does probably not change due to magnetic interactions: 2GS -> 2LMCT-> 2(2GS antiferromagnetically coupled with 3(pi-pi*(pyrene)). The DTET process can be easily described by a Dexter double electron exchange keeping the overall spin (see point 5).

The referee is right. The DTET process would be spin allowed when the total spin is considered. We removed the questioned sentences from the text.

12) The entire discussion and tentative assignments and TA interpretation might strongly profit form quantum chemical calculations, in particular TDDFT.

TDDFT computational studies have been performed on ***1-3*** *and on the free ligands phtmeimb,* ***A****, and* ***B*** *and are reported in* ***Supplementary Discussion - Part 2 (Figs. S36-S51*** *and* ***Tables S4-S11)****, see also answer to point 5. The computational study was quite time-consuming, since the*

*number of excited states to be computed and analyzed for these revealed to be much larger than expected (and, to our knowledge, never reported before in detail, in spite of the large interest in these Fe(III) species). Due to the complexity of the systems, more detailed TDDFT computational studies have focused only on compound **2** and in the identification of a possible equilibration between a ^{2}LMCT state and a triplet pyrene-centered state. It was possible to identify two relaxed excited states corresponding respectively to a ^{2}LMCT state and a triplet pyrene differing by only 0.1 eV. Though the energy of both these states is overestimated with respect to experimental emission energies (consistently with the overestimation of the absorption and emission energies normally obtained with the level of theory chosen), the calculations support that an equilibration between these two excited states is possible and could be correlated with the observed prolonged luminescence (transient absorption experiments, anyway, demonstrated that electron transfer dominates the equilibration process). Part of this discussion is reported in the main text (**from page 4, last two lines, to page 5, line 10,** see also answer to point 5).*

13) The TA spectra should be simulated from the spectroelectrochemical measurements by depicting the respective difference spectra.

Spectroelectrochemical measurements (experimental and simulated) would have been useful in the case of symmetry-breaking. In the present case, in which a pyrene$^+$-Fe(II) charge separated state is formed, are useless. In fact, the first oxidation of the complexes leads to Fe(IV), not to pyrene radical cation, and the spectra of the Fe(II) species formed by reduction is well known (see ref. 16) and fits with the transient absorption produced in the < 400 nm region. So, spectroelectrochemistry is not reported.

14) "Therefore, in the transient absorption spectrum of 3 recorded after 3 ps from pulse (Figs. 4b and S23), the band at about 500 nm can be due to triplet-triplet pyrene-based transitions, the band at about 560 nm can receive main contribution from the 2LMCT state, and the intense band at about 460 nm can receive contributions from transitions involving both pyrene triplet and LMCT states. This tends to suggest that the pyrene excimer state deactivates to the lower-lying pyrene-based triplet and LMCT states."
This section is really hard to understand. It is not clear to this reviewer, what the authors want to explain here.

As above mentioned, many more TA experiments have been made and the discussion of the results has been totally re-written. We thank the referee for his/her comments, that pushed us to largely implement our data and discussion.

15) The formal positive charges for 1-3 (Fig. 1) are wrong.

Figure 1 has been corrected.

16) Ref. 35 “and ref. cited therein” If these references are of significant importance, then they should be cited individually.

Ref. 35 is a review article in which data concerning many, mainly Ru(II), compounds exhibiting the excited-state equilibration due to reversible energy transfer are reported. The sentence “and cited therein” was added for completeness, but there is no need to individually report any single paper, since the main aspect of the process are already discussed, and the pioneering papers are already cited in refs. 27-34. To avoid any doubt, we erased the “and refs. cited therein” sentence.

17) Ru(bpy)32+: brackets are missing => [Ru(bpy)3]2+

This error has been corrected.

18) phtmeimb = {phenyl[tris(3-methylimidazol-1-ylidene)]borate} anion -> minus missing phtmeimb-

We define phtmeimb as the anion of the ligand, so minus is not needed. This also helps to simplify the formula of the compounds.

19) In the conclusion, the authors estimate (on the basis of a 7 ns lifetime, 100% !) a Stern-Volmer quenching rate increase. However, this has not been shown experimentally and correspond quenching experiments need to be conducted to prove such an effect.

In the original version of the paper, we mentioned the ***theoretical*** *increase in the efficiency of bimolecular reactions prolonging the excited-state lifetime from 2 ns to 7 ns, according Stern-Volmer equation (clearly, in this theoretical situation in both cases the quenching rate constant is assumed to be diffusion-controlled, for homogeneity). We did not claimed that the present compounds exhibit such an increase, but we wished to stress the practical importance of prolonging the excited-state lifetime, even when the prolongation is not very large (that is on passing from 2 to 7 ns). However, we removed this sentence from the paper, since it could originate misunderstanding.*

Reviewer #3:

The manuscript by Campagna and co-workers reports long-lived luminescence from iron(III) complexes with pyrene moieties in fluid solution at room temperature. Various pyrene-bound luminescent metal complexes exhibiting long-lived luminescence have been reported in the past 30 years. For example, the emission lifetime of a ruthenium(II) complex with three pyrene moieties is as long as 9.0 μs as reported by Tyson and Castellano. In these systems, the pyrene moiety acts as an energy reservoir, and the emitting excited state is generated via the intramolecular energy transfer from the long-lived triplet excited state of the pyrene moiety. The authors introduced the pyrene moieties into a luminescent iron(III) N-heterocarbene complex and succeeded in obtaining long-lived luminescence up to 6.5 ns at room temperature. The luminescence lifetime is significantly long in comparison to other iron(III) complex. The excited states with a >5-ns lifetime is utilizable as a photosensitizer in a variety of solar–energy conversion systems and, therefore, this work may open the door to use earth-abundant iron(III) complexes. However, the lifetime being 6.5 ns for complex 3 in dichloromethane is not necessarily surprising and very long since that of the reported complex without the pyrene moiety (complex 1) is 2.4 ns under the identical conditions. Furthermore, the experimental evidence for the excited-state equilibration is not sufficient. Transient absorption data for both complexes 2 and 3, unfortunately, exhibited another photophysical processes due to aggregation arising from high sample concentrations. Temperature-dependent steady-state and time-resolved luminescence measurements were also not performed. Thus, the reviewer suggests that the manuscript is not suitable for a publication in Nature Communications at this stage.

The referee agrees that we obtained long-lived luminescence for Fe(III) complexes in fluid solution by successfully employing for the first time (at the time of the initial submission) the excited-state equilibration approach to a transition metal complex made of an earth-abundant metal, and this finding can be relevant for applicative reasons.

We implemented transient absorption spectroscopy experiments to add more experimental evidence for the excited-state equilibration we propose. By performing such new experiments (also using different set-up for our fs experiments and using a recently purchased flash-photolysis ns apparatus that allowed us to explore a larger wavelength range and longer time ranges) we discovered that the excited-state equilibration in our complexes is driven by electron transfer and not by energy transfer (this latter being the usual process leading to excited-state equilibration, as also mentioned by the referee in his/her comments). In our opinion, this introduces in the paper an even more important novelty in comparison with the original submission, and we also believe that transient absorption data represent now solid experimental evidence for complex ***3****, whose properties are not affected by aggregation. Indeed, aggregation (only occurring in* ***2****) makes difficult direct comparison*

*between luminescence data and transient spectroscopy of **2**, however the strong similitude between the properties of **2** and **3** (and the fact the same electron transfer process also occurs in the aggregate **2** species, as indicated by transient spectroscopy) supports the proposed hypothesis that a similar electron transfer-driven excited-state equilibration also occurs in the "isolated" **2** species and is responsible for its biphasic luminescence decay. Actually, compound **3** alone would be enough to support the main findings of the paper, but we believe that the results on **2** add many useful information – also in comparison with **3** - which can be useful to the community.*

We agree that the increase in lifetime is not surprisingly longer when compared to the model species, but as the same referee says, "the 6.5 ns lifetime is significantly long in comparison to other iron(III) complexes and that exceeding 5 ns can open the way to application to a variety of solar-energy conversion systems". According to this latter sentence by the referee, the relatively small increase in luminescence lifetime appears to be significative anyway. Moreover, we would like to stress that it derives from an unusual process involving the participation of a charge-separated state which is unprecedent in the field of earth-abundant metal complexes (with only a unique precedent for transition metal complexes in general).

As far as temperature-dependent steady-state and time-resolved luminescence experiments are concerned, we agree that such kind of experiments could add interesting details on the knowledge of the photophysical behavior of the studied complexes, but these experiments would require equipment that is not available in our laboratories. Moreover, they surely should expand the present work at a level that would be difficult to include in a single communication (experimental determination of activation energies, dependence of charge recombination on temperature, effect of solvent changes, etc). Therefore, these topics can be investigated only at a later stage.

We revised the manuscript by considering the comments of reviewer 3. Our answer is below:

Comment by Reviewer 3:

The authors performed additional experiments such as nanosecond transient absorption and, as results, detailed excited-state dynamics, including nature of the biexponential emission decays, of iron(III) complexes with pyrene moieties were revealed. The long-lived excited state by utilizing an equilibrium between the emitting and charge-separated states is fascinating strategy toward future design of the luminescent complexes of redox-active iron and enhances applicability of earth-abundant metal complexes. The present manuscript is now publishable in Nature Communications. The reviewer concerns the presence (or contribution) of the spin quartet state(s) in the excited-state dynamics of the complexes. The theoretical calculations added in the revised manuscript suggest that there are lower-lying quartet states than the doublet excited states. As several JACS papers have recently reported that charge-separation process plays an important role in the spin-state modulation in organic compounds bearing a spin-doublet substituent(s), charge separation in the present system being spin-doublet iron(III) with aromatic hydrocarbon might possess significant contribution to the excited-state dynamics of the complexes. Including the descriptions of the presence and contribution of the quartet states is recommended (additional experiments are not required).

Answer:

Indeed, our calculations show that low-lying quartet states are present both for the vertical excitation and at the relaxed geometries. Nonetheless, the energy difference between these low-lying quartets and first doublet is computed to be relatively high suggesting that the quartets contribution to the doublet excited state dynamic should be minor.

To clarify this point we have added the following sentence in the main text (**page 4, lines 14-16)**:

At TD-DFT level the presence of low lying quartet states is also evidenced but their contribution to the doublet excited state dynamics is expected to be small due to their significant energy gap.

We also followed the editorial requests in revising the manuscript.

With Kind Regards,

Sebastiano Campagna
